# Interactive analysis of single-cell trajectories in 3D space with Cell Journey

**DOI:** 10.1093/gigascience/giag021

**Published:** 2026-03-03

**Authors:** Damian Panas, Marcin Tabaka

**Affiliations:** International Centre for Translational Eye Research, Skierniewicka 10A, Warsaw 01-230, Poland; Institute of Physical Chemistry, Polish Academy of Sciences, Kasprzaka 44/52, Warsaw 01-224, Poland; International Centre for Translational Eye Research, Skierniewicka 10A, Warsaw 01-230, Poland; Institute of Physical Chemistry, Polish Academy of Sciences, Kasprzaka 44/52, Warsaw 01-224, Poland

**Keywords:** single-cell multiomics, developmental trajectory, RNA velocity

## Abstract

The integration of high-throughput single-cell profiling technologies with RNA velocity analysis has enabled the reconstruction of dynamic cellular differentiation trajectories at unprecedented resolution. Despite these advances, current visualization techniques for RNA velocity are predominantly confined to 2-dimensional representations, typically employing arrows or streamlines. While effective for depicting simple cellular trajectories, these approaches are insufficient for capturing the complex topologies of multipartite cellular transitions. This limitation highlights the need for advanced 3-dimensional visualization tools that can more accurately convey the structure and dynamics of velocity-inferred transitions in single-cell data. Here, we present Cell Journey, an interactive visualization platform specifically developed for 3-dimensional analysis and representation of RNA velocity trajectories derived from single-cell datasets. The platform features an intuitive graphical interface supporting both unimodal and multimodal data, accommodates multiple input formats, and provides extensive customization capabilities for trajectory visualization. Cell Journey computes RNA velocity vector fields on a user-defined 3-dimensional grid and constructs velocity trajectories using either Euler integration or the fourth-order Runge–Kutta method. The platform enables dynamic exploration of cellular dynamics through interactive visual elements, including streamlines, streamlets, cones, and volumetric plots. Furthermore, it allows users to investigate changes in feature activity along selected paths, facilitating deeper insights into cellular state transitions within complex multimodal single-cell datasets.

## Introduction

Single-cell RNA sequencing technologies have enabled the study of cellular differentiation trajectories at unprecedented resolution [1–4]. These methods capture static snapshots of cellular transcriptomic states, with dynamic transitions between states subsequently inferred through computational analysis. Notably, RNA velocity-based approaches have emerged as state-of-the-art tools for uncovering the directionality of cellular transitions [5–21]. In single-cell RNA sequencing data, newly transcribed (unspliced) transcripts retaining intronic sequences can be reliably distinguished from fully spliced, mature transcripts. RNA velocity analysis exploits the quantitative relationship between nascent and mature RNA molecules. By aggregating gene-specific transcriptional dynamics across the transcriptome, RNA velocity enables the prediction of future transcriptional states, effectively forecasting a cell’s trajectory in gene expression space over short time scales. RNA velocity has emerged as one of the most influential frameworks for inferring cellular differentiation pathways, disentangling subpopulation kinetics, elucidating lineage relationships, and visualizing dynamic developmental processes. Its introduction has spurred the development of numerous computational methods for trajectory inference [8, 22–27], as well as advanced visualization algorithms that incorporate RNA velocity information to represent cellular dynamics [28–31]. In recent years, we have witnessed the development of multimodal single-cell sequencing technologies that co-profile from the same cell various combinations of genome-wide features such as transcriptome, chromatin accessibility, histone modifications, and protein epitopes [32–46]. These methods offer an opportunity to understand the temporal relationship between different layers of gene expression regulation and increase the potential of determining cell states. Incorporation of chromatin state switch times to RNA velocity framework improves the accuracy of cell fate prediction compared to velocity estimates from RNA only [10].

**Figure 1:**
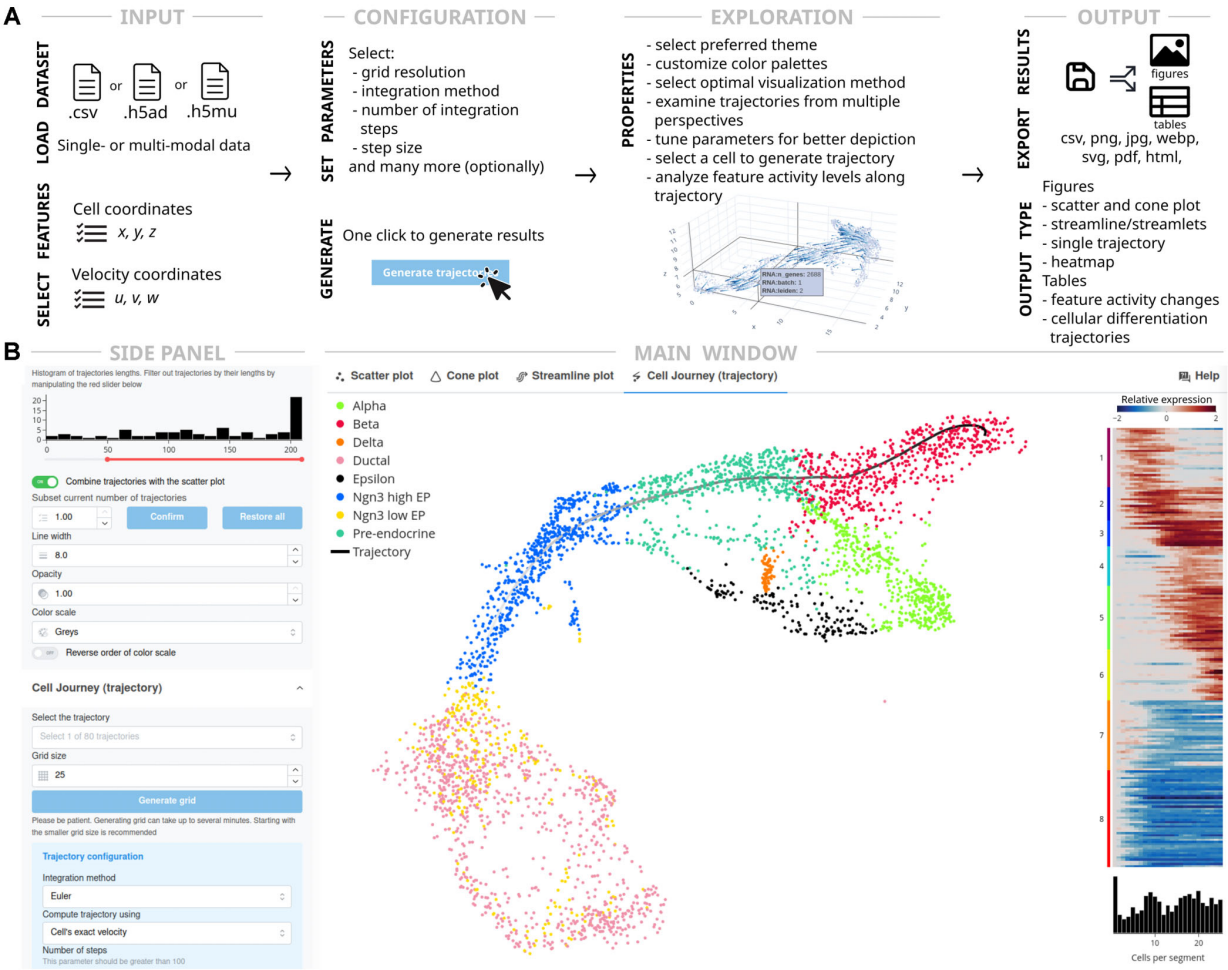
Cell Journey is an interactive tool for visualization and exploratory analysis of single-cell multiomics data and velocity-based trajectories. (A) Overview. Cell Journey accepts as input (left) single-cell datasets in text or hdf5 file format (h5ad or h5mu for uni- or multimodal data). Users in the first step load the data and select the variables of cell coordinates and the components of the velocity vectors. After defining the grid resolution and integration method, Cell Journey computes RNA velocity vector field lines in a 3D cell embedding. Then, the user can explore the visual depiction of the vector field, generate trajectories for a selected cell, and study the modality feature changes along the computed trajectory. Finally, generated figures can be exported in a high-resolution publication-ready format. (B) A part of the interface with a generated trajectory from a selected cell. The software has a main window and a drop-down panel on the left. The fully customizable heatmap shows activities of modality features (e.g., gene expression) along the trajectory.

The crucial analytical task in RNA velocity analysis is the calculation of cell’s transition probability in high-dimensional space and its subsequent projection onto a low-dimensional embedding. The transitions between the cellular states are often visualized as arrows or streamlines on a 2-dimensional (2D) cell embedding. However, 2D cell embeddings can result in significant topological misrepresentations [47]. In the context of complex developmental single-cell data, such 2D representations are often insufficient to capture the intricate topologies of multipartite continuous cellular transitions [31]. Cell Journey addresses these limitations by computing and interactively visualizing single-cell velocity-based trajectories in 3-dimensional (3D) space (Fig. 1A). Cell Journey computes RNA velocity vectors on a user-defined grid to capture spatially resolved transcriptional dynamics. It then constructs 3D field lines by numerically integrating these vectors, offering a choice between the Euler method and the fourth-order Runge–Kutta algorithm. The Euler method provides a straightforward, stepwise approximation of the cell trajectory, while the fourth-order Runge–Kutta approach delivers enhanced accuracy by accounting for intermediate evaluations within each integration step. This dual-method framework allows researchers to balance computational efficiency and precision when modeling the complex dynamics of cellular state transitions.

**Figure 2:**
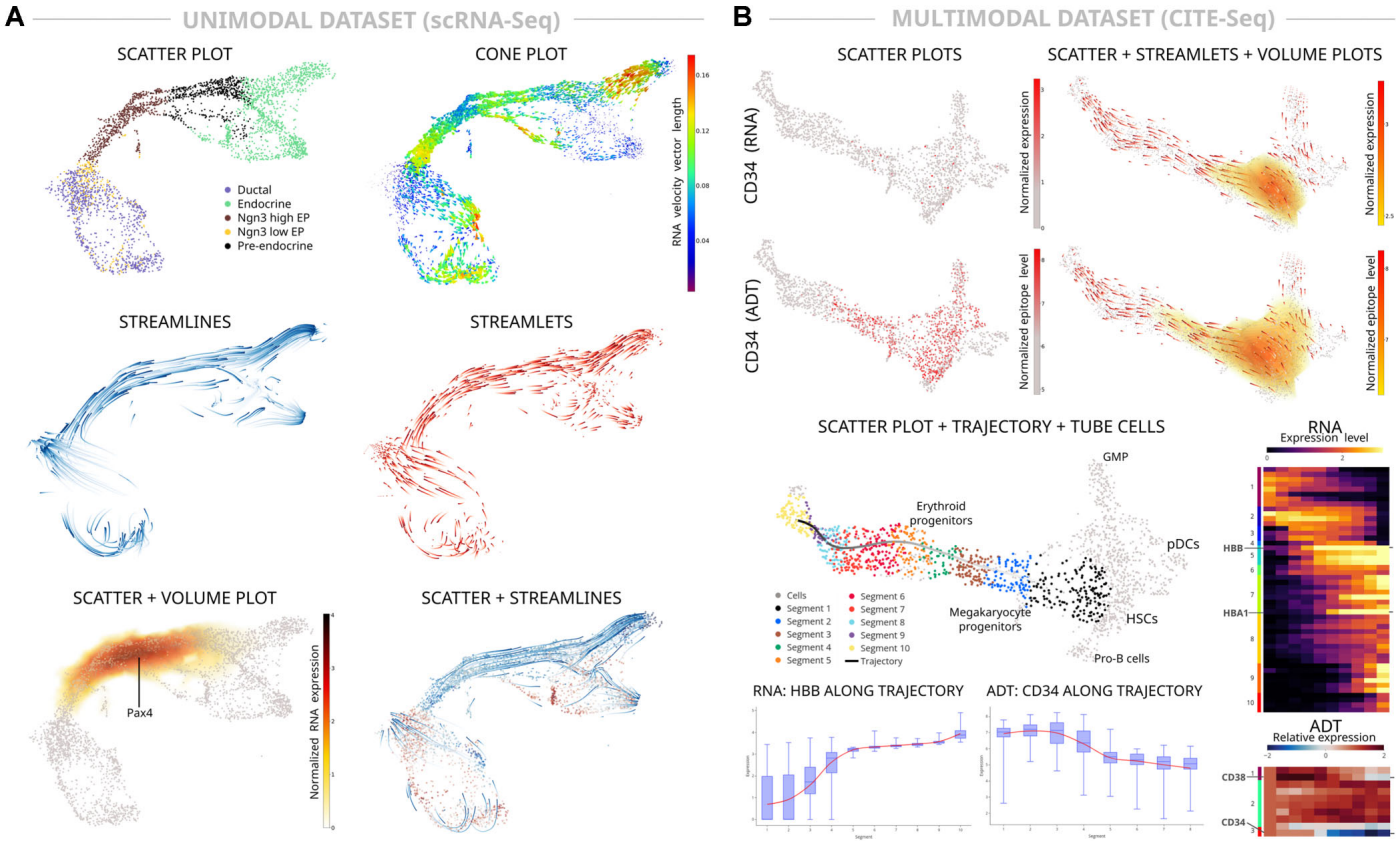
Cell Journey implemented visualizations of 3D single-cell embeddings and RNA velocity vector fields applied to (A) a unimodal scRNA-seq dataset of pancreatic endocrinogenesis [48], and (B) a multimodal CITE-seq dataset of human bone marrow mononuclear cell progenitors [49]. Cells in the neighborhood of the generated trajectory are grouped into user-specified segments to calculate the statistics of the feature activities. The interactive heatmap with grouped feature activities along the trajectory displays when clicking on a selected feature the trend of its activity in multiple formats.

Cell Journey is engineered to serve single-cell researchers, irrespective of their computational proficiency, by providing a platform that simplifies the exploration of single-cell datasets in 3D. Its intuitive and accessible graphical user interface promotes seamless interaction and efficient data analysis (Fig. 1B). The software employs a state-of-the-art visualization platform to render computed 3D trajectories using graphical aids such as streamlines, streamlets, and cones (Fig. 2A). Additionally, interactive 3D scatterplots enable the dynamic computation and visualization of differentiation trajectories, either initiated from a selected cell or generated across a user-defined regular grid to mitigate the single-cell data sparsity. This functionality enables researchers to interactively explore complex trajectories and efficiently assess multimodal feature activity changes along selected trajectories (Fig. 2B).

## Materials and methods

To demonstrate its utility, Cell Journey was applied to visualize RNA velocity-based inferred trajectories from 2 representative datasets: a unimodal scRNA-seq dataset of mouse pancreatic endocrinogenesis [48] (GSE132188, Fig. 2A) and a multimodal CITE-seq dataset of human bone marrow mononuclear cell (BMMC) progenitors [50] (GSE128639, Fig. 2B). Unimodal scRNA-seq pancreatic endocrinogenesis was processed with scVelo 0.2.5 [6]. The package was also used to preprocess the data and estimate RNA velocity components. Preprocessing consisted of applying filter_and_normalize function with min_shared_counts parameter equal to 20, and n_top_genes equal to 2,000. Next, the moment function was applied with n_pcs and n_neighbors parameters both equal to 30. The UMAP embedding was calculated using Scanpy 1.9.6 [51] with n_components parameter equal to 3. Finally, cell velocities were projected into the UMAP using scVelo’s velocity_embedding function. CITE-seq multimodal human bone marrow data were preprocessed with CITE-seq-Count v1.4.5 [45]. The obtained RNA count matrix was preprocessed with Scanpy. The following functions were applied: filter_cells with min_genes equal to 100, filter_genes with min_cells equal to 3, normalize_total and log1p with default parameters, highly_variable_genes with n_top_genes set to 5,000. Next, pca and neighbor functions were applied with the default parameters, and the 3D UMAP embedding was calculated using the umap function with n_components parameter equal to 3. Finally, RNA velocity was inferred using UnitVelo 0.2.5.2 [7] with N_TOP_GENES parameter equal to 1,000 and R2_ADJUST set to False.

**Figure 3:**
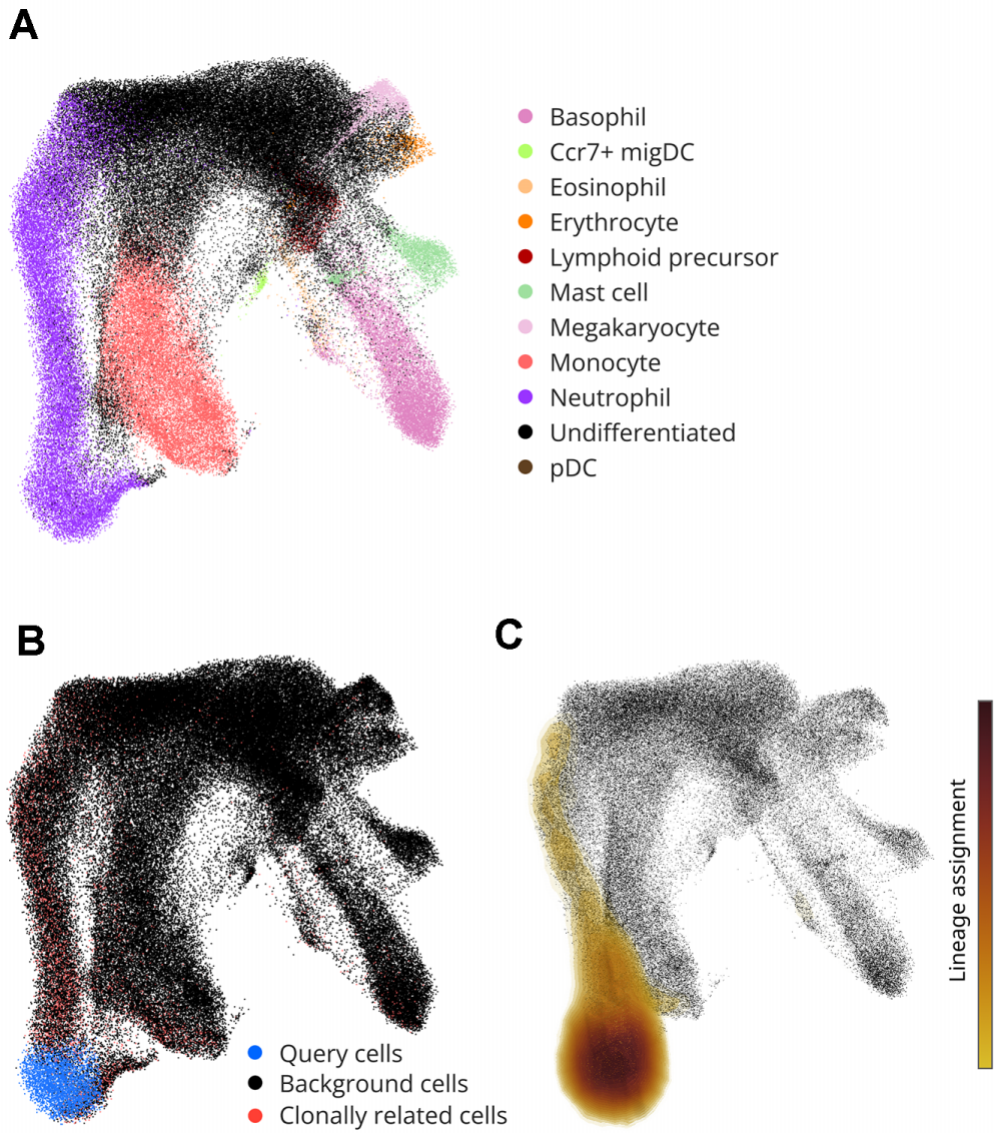
Example application of Cell Journey for lineage tracing analysis using clonal scRNA-seq data from Weinreb et al. [52]. (A) 3D UMAP visualization with cell-type annotations. (B) Identification of all cells assigned to the same clonal lineage as the cells in a user-defined region selected by a single click. To maintain full analytical control, Cell Journey highlights the query cells and their clonally related cells separately. (C) Visualization of the same data as a volume plot facilitates reconstruction of a developmental trajectory.

Furthermore, we demonstrated Cell Journey’s utility for clonal data analysis using the dataset from Weinreb et al. [52] (GSE140802, Fig. 3). Clonal gene expression data and cell annotations were retrieved from Allon Klein’s lab GitHub repository. The data were processed using a standard Scanpy pipeline: count matrix was log-transformed using log1p function. 2,000 highly variable genes were identified with highly_variable_genes function, and then principal component analysis was conducted using the pca function. Subsequently, the neighborhood graph was constructed with the neighbors function (n_neighbors = 20 and n_pcs = 30). Finally, a 3D UMAP embedding was generated using the umap function with the n_components parameter set to 3.

## Results

Cell Journey is implemented in Python 3.11.7 and leverages a robust ecosystem of libraries to ensure both functionality and user-friendliness. The core dashboard is constructed using Dash, with Dash Mantine Components and Dash Bootstrap Components enhancing the interactivity and aesthetic of the user interface. Interactive visualizations are generated with Plotly, while SciPy provides essential numerical routines, including linear and radial basis interpolation, linear smoothing, and nearest-neighbor lookups. Scikit-learn is utilized for *k*-means clustering of computed averaged feature activity trends, and Scanpy [51] supports comprehensive processing of single-cell data. The platform also integrates MuData [53] for handling multimodal datasets, with NumPy and Pandas managing array operations and data frames, respectively. Coloraide is employed to interpolate color palettes, ensuring visual consistency throughout the analyses. Cell Journey focuses on interactive 3D visualization and exploratory analysis of cellular transitions and therefore builds on widely used Scanpy/AnnData-based workflows for standard preprocessing. We note that Cell Journey is complementary to a broad ecosystem of upstream single-cell frameworks for representation learning and denoising [54, 55], whose outputs (e.g., embeddings and processed feature matrices) can be readily visualized within Cell Journey. This cohesive integration of Python-based computational tools enables Cell Journey to deliver scalable and precise analyses of single-cell developmental data.

The platform is engineered to address key tasks in the exploration and visualization of single-cell data (Fig. 1A). It accommodates the upload of diverse dataset formats—including h5ad for single-modality, h5mu for multimodal, and comma-separated CSV dataset files—thus ensuring broad compatibility with various data sources. It enables the visualization of cells embedded in 3D space, where representations can be based on either categorical or continuous feature activity values, such as gene expression, protein epitope levels, or cell cluster annotations. The 3D embeddings of single-cell data can be generated by an arbitrary method, including UMAP [56], FLE [57], or hyperbolic ones such as scPhere [58]. Furthermore, the platform addresses challenges in visualizing low-abundance features and compensates for dropout artifacts inherent in single-cell profiling by rendering feature activity values as partially transparent isosurfaces (volumetric/volume plots) within the 3D embedding (Figs 2 and 3). Furthermore, the volume plot provides a mechanism for interpolating any desired feature by means of a selection of radial basis functions (including Gaussian, linear, quadratic, or multiquadratic). The smoothing level of the resulting approximation can be accurately controlled via multiple independent parameters, thereby offering a high degree of flexibility in adjusting the level of precision and computational efficiency. These volume plots are fully customizable, allowing users to independently adjust the grid resolution—separate from the grid used for trajectory computation—select appropriate color palettes, and define the range of feature activity values displayed. This level of customization ensures that subtle variations in feature activities are effectively visualized, thereby enhancing the interpretability of low-abundant features like expression levels of transcription factors or surface proteins. Such volume plots become especially important in large-scale 3D visualizations, where small point sizes representing cells and low detection probabilities of features can impede the visualization of activity levels in conventional scatter plots. Extensive customization options for scatterplots are provided, allowing users to define specific scales or color palettes, or to select from an array of built-in options, including those optimized for colorblind accessibility. Additionally, figures can be exported either as static images in raster and vector formats or as interactive visualizations suitable for exploration in a web browser. Finally, Cell Journey offers a flexible interface that supports dynamic zooming of cells and trajectories, with real-time updates following adjustments to visual parameters.

To facilitate the visualization of cellular transitions in 3D single-cell data embeddings, Cell Journey calculates RNA velocity vectors on a regularly spaced grid with user-defined resolution. These vectors are then used to compute 3D field lines through numerical integration, employing either the Euler method or the fourth-order Runge–Kutta algorithm. Researchers can regulate both the step count and the step size of these algorithms to obtain an optimal trajectory length. In particular, implementing parameters such as the scale grid and the difference threshold enables a fine-grained balance between computational speed and the level of detail captured in the integration. Users can flexibly adjust the density of the streamlines, generate streamlets, and customize their attributes—including length, color, transparency, and color gradients depicting direction of trajectories. Users can select specific trajectories for detailed analysis. Moreover, trajectories can be dynamically generated from any selected cell or grid element within the scatterplot. The tool further quantifies changes in uni- or multimodal feature activity levels—such as gene expression profiles or epitope levels—along the selected or generated trajectory. These activity changes are clustered according to their trends and subsequently visualized in an interactive heatmap (Fig. 2B), providing a comprehensive overview of the dynamic cellular processes at genome-wide scale. The sequence of clusters is first determined by grouping the up- and down-regulated averaged profiles and then by ordering their extremal values. This approach yields well-defined temporal groupings of features that exhibit gradual transitions, enabling more precise characterization of dynamic patterns over time.

To evaluate the distinctive capabilities of Cell Journey within the landscape of interactive single-cell data analysis tools, we conducted a comprehensive comparison with a range of existing platforms (Table 1), including ASAP [59], cellxgene [60], Corvo [61], SCope [62], scSVA [63], singlecellVR [64], StarmapVis [65], the UCSC Cell Browser [66], and Vitessce [67]. While several of these tools offer limited support for 3D visualization of single-cell data, Cell Journey stands out as the most comprehensive platform in terms of functionality and analytical depth. It is the only tool capable of generating and rendering RNA velocity outputs directly in 3D space. Furthermore, it uniquely supports fully interactive computation, visualization, and exploration of cellular trajectories based on RNA velocity, offering an integrated platform for trajectory inference and dynamic state analysis.

**Table 1:** Comparison of the functionality of Cell Journey with other platforms for interactive exploration and analysis of single-cell data.

		Cell Journey	ASAP	CELLxGENE	Covro	SCope	scSVA	singlecellVR	StarmapVis	UCSC Cell Browser	Vitessce
RNA velocity	Cone plot	**+**									
	Volume plot	**+**									
	Streamlines/streamlets	**+**									
	Combining multiple types of plots	**+**									
	Trajectory from selected cell	**+**									
	Feature changes along the trajectory	**+**									
General	3D view	**+**	+		+		+	+	+		+
	Multimodality	**+**			+			+			+
	Docker	**+**	+	+		+	+	+			
	Example datasets	**+**	+	+		+		+	+	+	+
Input files	h5		+				+				
	h5ad	**+**		+	+		+	+		+	
	h5mu	**+**									
	csv/tsv/txt	**+**	+				+		+	+	+
	loom		+			+	+	+		+	
	Seurat/rds		+							+	
Processing	Clustering	**+**	+								+
	Data normalization	**+**	+			+					
	Scaling	**+**	+			+					
	Feature/cell filtering	**+**	+	+	+	+			+	+	

We next demonstrate the application of Cell Journey to single-cell transcriptomic and multi-omic datasets, illustrating its capability to resolve complex cellular dynamics through integrated 3D visualizations. Figure 2A illustrates the 3D visualization of pancreatic endocrinogenesis, where an example trajectory is generated from a selected endocrine progenitor cell progressing toward beta cells. Gene expression dynamics along this trajectory are clustered using *k*-means based on their temporal trends and subsequently visualized via an interactive heatmap. The cone plot, which projects the direction of cellular transitions within the 3D embedding, is highly dependent on the precision of detecting unspliced and spliced RNA forms of key genes and consequently displays heterogeneous transition probability patterns. In contrast, streamlines and streamlets, derived from the integration of individual cell velocity vectors, yield more consistent trajectories toward fully differentiated cells. Additionally, the volume plot effectively depicts the expression level of Pax4, a key transcription factor for endocrine development [68], which is transiently expressed in endocrine progenitors [69]. For the visualization of the BMMC CITE-seq dataset in 3D (Fig. 2B), 3 modes are employed simultaneously: a scatter plot to display cell positions, streamlets to indicate the direction of cellular transitions, and a volume plot to portray the epitope levels of the CD34 surface marker, which is specific to hematopoietic stem cells (HSCs). Although epitope levels are readily visualized in conventional scatterplots, RNA expression is substantially sparser. Nonetheless, the volume plot derived from RNA modality data accurately reflects its HSC specificity, highlighting the utility of volume plots in delineating low-abundance features. In a subsequent visualization, the same plot orientation is used to generate a trajectory from a selected cell to erythroid progenitor cells. Segments of trajectory-projected cells are marked to compute differentially expressed feature activity levels, which are then grouped according to their trends and visualized in interactive heatmaps. Users can select the modality for analysis—such as RNA or ADT (epitope) levels—and the heatmaps can display either normalized or relative feature activity levels. Notably, the RNA heatmap highlights a pronounced upregulation of hemoglobin subunit beta (HBB) and hemoglobin alpha 1 (HBA1), components of hemoglobin A, along the erythroid lineage. Conversely, the ADT heatmap reveals a systematic decrease in CD34 protein levels from HSCs, alongside a transient upregulation of CD38 levels along the erythroid trajectory. These observations underscore the platform’s robust capacity to elucidate dynamic cellular processes across multiple data modalities.

Finally, we demonstrate that Cell Journey can also be applied to clonal scRNA-seq lineage-tracing data, where “ground-truth” relationships between cells are provided by shared barcodes rather than inferred velocities. Using the DNA-barcoding dataset from Weinreb et al. [52] (Fig. 3A), Cell Journey enables interactive identification and visualization of clonally related cells directly within a 3D embedding (Fig. 3B–C). Specifically, a user can select a region of interest with a single click (Fig. 3B), after which the platform highlights the query cells and all cells assigned to the same clonal lineage (neutrophil lineage in Fig. 3B), while keeping background cells visible to preserve global context. Importantly, because barcode-based lineage tracing does not provide vector-valued directionality in transcriptional space, analysis focuses on the distribution of clone membership across cellular states. In this setting, representing clonal enrichment as a volume plot (Fig. 3C) provides a smooth, continuous depiction of clone-associated regions over the manifold, which can be difficult to interpret from sparse point overlays alone, particularly when clonally related cells are rare or dispersed. The number and spread of clonally related cells across states can provide constraints on likely state-to-fate relationships (e.g., clonal expansion and shared ancestry across compartments), and Cell Journey facilitates interactive exploration of these patterns in 3D.

**Figure 4:**
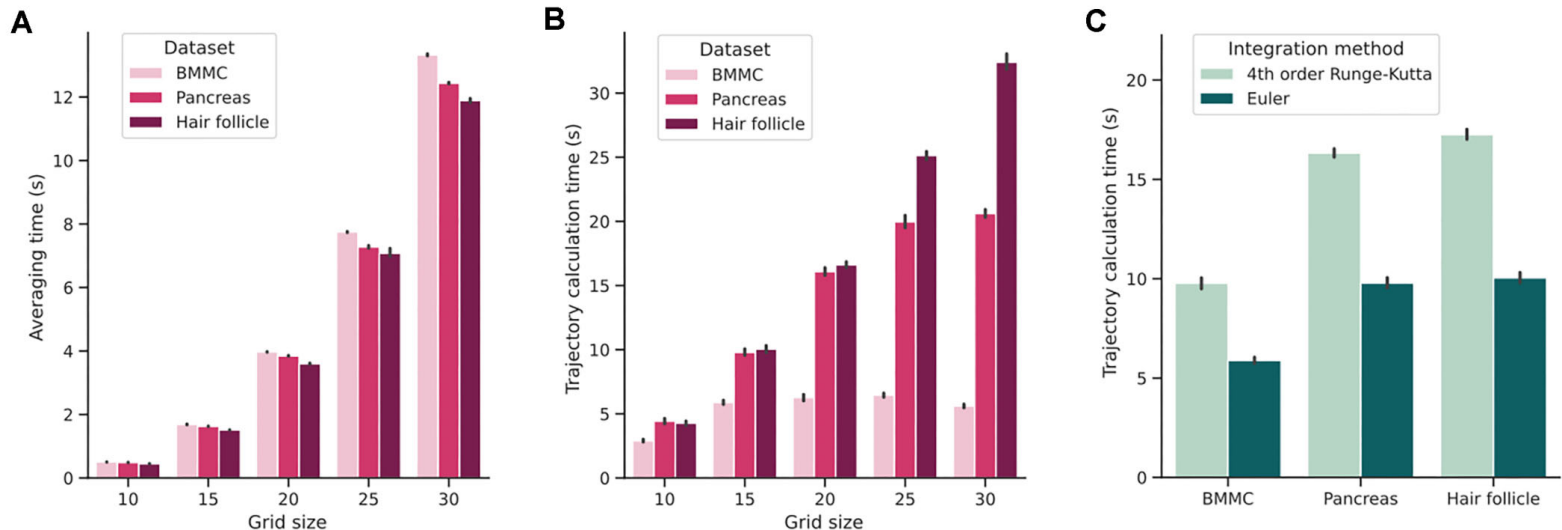
Computational efficiency of trajectory vector-field averaging and trajectory integration. (A) Benchmarking Euler integration across varying grid densities. (B) Comparative performance of different integration methods at a fixed grid size of 15. Bars represent the mean execution time over 10 independent runs, with whiskers denoting 95% confidence intervals (CIs). These CIs were estimated via the bootstrap method using the Seaborn Python package [70]. All benchmarks were conducted on a Dell Latitude X1201 (16 GB RAM) running the Debian 13 operating system. Datasets: BMMCs—human bone marrow mononuclear cell progenitors [49]; pancreas—pancreatic endocrinogenesis [48]; hair follicle—regenerative compartment of the hair follicle [34].

To assess the computational efficiency of Cell Journey during its most demanding tasks, we performed benchmarking across various datasets, integration methods, and grid sizes (Fig. 4). Our findings indicate that both the integration method and grid resolution influence computation time; however, both vector-field averaging and trajectory integration remain efficient (Fig. 4A–B). Even at higher grid resolutions, execution times stay within a range that allows for near-instantaneous analysis without prolonged waiting periods. While we observed a non-linear increase in processing time as the grid resolution increases, the absolute duration remains low across all tested datasets. Furthermore, comparing integration strategies revealed that the Euler method is significantly more efficient than the fourth-order Runge–Kutta, offering approximately a 40% reduction in processing time (Fig. 4C). Consequently, we recommend that users begin data exploration using the default parameters and subsequently adjust them according to their available computational resources to achieve the desired balance between detail and speed.

## Discussion

Our objective is to facilitate rapid and in-depth exploration of single-cell developmental data in 3D, thereby providing immediate insights and fostering a nuanced understanding of cellular trajectories. Cell Journey is accessible to researchers regardless of computational expertise, featuring a streamlined setup process that requires only a few simple steps and an intuitive, user-friendly interface. A comprehensive help panel, accompanied by FAQs and sample datasets, enables users to quickly familiarize themselves with the tool’s functionalities. Designed with extensibility in mind, Cell Journey accommodates the integration of additional analytical modules, thereby supporting increasingly comprehensive analyses. Moreover, the software is optimized for speed, efficiency, and robustness, ensuring reliable performance even when processing large-scale single-cell datasets. Cell Journey is under active development. We plan to extend its functionality by providing new modules for differential trajectory analysis and integration with workspaces for comprehensive single-cell multimodal data analysis. We actively invite user feedback and feature suggestions to drive the continuous enhancement of its functionality, and we are committed to providing regular updates and fostering active community engagement to ensure the tool’s ongoing relevance and innovation.

While Cell Journey was originally developed to visualize transitions between cellular states inferred from RNA velocity analyses [71], its framework is broadly applicable to any approach, experimental or computational, that delineates cellular state transitions. In particular, Cell Journey can be used to visualize ground-truth transitions and lineage relationships derived from lineage-tracing experiments. As demonstrated in Fig. 3, Cell Journey supports interactive exploration of DNA-barcoding clonal scRNA-seq data (e.g., Weinreb et al. [52]; see also related DNA-barcoding approaches [72]), enabling users to highlight clonally related cells and reconstruct lineage trajectories directly on transcriptional embeddings. More generally, Cell Journey is also compatible with other lineage-tracing modalities, including Cas9-based cellular ancestry recording [73]. These experimental strategies label cells with heritable genetic markers, allowing reconstruction of clonal dynamics and lineage relationships from shared barcodes or mutation patterns, which can then be visualized and interrogated within Cell Journey. Furthermore, Cell Journey can be potentially used to integrate and visualize trajectories from transition probabilities between cells computationally inferred using optimal transport algorithms applied to time-course single-cell transcriptomic data [3] or multimodal or spatial datasets [74]. Finally, we foresee that Cell Journey will be instrumental in visualizing putative cellular transitions within 3D spatial transcriptomics datasets [75], particularly when combined with emerging methodologies for spatial RNA velocity analysis [76–78]. This versatility underscores the potential of Cell Journey to facilitate a comprehensive understanding of cellular dynamics across diverse experimental and computational platforms.

## Availability of source code and requirements

- Project name: Cell Journey.
- Project homepage: https://github.com/TabakaLab/CellJourney.
- Documentation: https://tabakalab.github.io/CellJourney.
- Operating system(s): Platform independent.
- Programming languages: Python.
- Other requirements: Python 3.11.7, Dash 3.3.0, Plotly 6.5.2.
- License: MIT license.

## Supplementary Material

giag021_Authors_Response_To_Reviewer_Comments_original_submission

giag021_GIGA-D-25-00322_original_submission

giag021_GIGA-D-25-00322_Revision_1

giag021_Reviewer_1_Report_original_submission Reviewer 1 -- 9/23/2025

giag021_Reviewer_2_Report_original_submission Reviewer 2 -- 9/24/2025

giag021_Reviewer_3_Report_original_submission Reviewer 3 -- 10/14/2025

## Data Availability

Cell Journey is under the MIT License. The software, documentation, tutorials, example datasets, and animated demos can be found at GitHub [79]. All additional supporting data are available in the *GigaScience* repository, GigaDB [80].
