# Supplementary material for: Interactive analysis of single-cell trajectories in 3D space with Cell Journey
Source: Gigascience. 2026 Mar 3;15:giag021. doi: 10.1093/gigascience/giag021 (PMC13042281; doi:10.1093/gigascience/giag021)
Supplement: giag021_GIGA-D-25-00322_original_submission [file giag021_giga-d-25-00322_original_submission.pdf]

GigaScience

Interactive analysis of single-cell trajectories in 3D space with Cell Journey

--Manuscript Draft--

**Manuscript Number:**	GIGA-D-25-00322
**Full Title:**	Interactive analysis of single-cell trajectories in 3D space with Cell Journey
**Article Type:**	Technical Note
**Funding Information:**	Fundacja na rzecz Nauki Polskiej (FENG.02.01-IP.05-T005/23) — Dr Damian Panas<br>National Science Center (2022/46/E/NZ2/0037) — Dr Marcin Tabaka
**Abstract:**	The integration of high-throughput single-cell profiling technologies with RNA velocity analysis has enabled the reconstruction of dynamic cellular differentiation trajectories at unprecedented resolution. Despite these advances, current visualization techniques for RNA velocity are predominantly confined to two-dimensional representations, typically employing arrows or streamlines. While effective for depicting simple cellular trajectories, these approaches are insufficient for capturing the complex topologies of multipartite cellular transitions. This limitation highlights the need for advanced three-dimensional visualization tools that can more accurately convey the structure and dynamics of velocity-inferred transitions in single-cell data. Here, we present Cell Journey, an interactive visualization platform specifically developed for three-dimensional analysis and representation of RNA velocity trajectories derived from single-cell datasets. The platform features an intuitive graphical interface supporting both unimodal and multimodal data, accommodates multiple input formats, and provides extensive customization capabilities for trajectory visualization. Cell Journey computes RNA velocity vector fields on a user-defined three-dimensional grid and constructs velocity trajectories using either Euler integration or the fourth-order Runge-Kutta method. The platform enables dynamic exploration of cellular dynamics through interactive visual elements, including streamlines, streamlets, cones, and volumetric plots. Furthermore, it allows users to investigate changes in feature activity along selected paths, facilitating deeper insights into cellular state transitions within complex multimodal single-cell datasets.
**Corresponding Author:**	Marcin Tabaka<br>Institute of Physical Chemistry PAS: Polska Akademia Nauk Instytut Chemii Fizycznej<br>Warsaw, POLAND
**Corresponding Author Secondary Information:**	
**Corresponding Author's Institution:**	Institute of Physical Chemistry PAS: Polska Akademia Nauk Instytut Chemii Fizycznej
**Corresponding Author's Secondary Institution:**	
**First Author:**	Marcin Tabaka
**First Author Secondary Information:**	
**Order of Authors:**	Marcin Tabaka<br>Damian Panas
**Order of Authors Secondary Information:**	
**Additional Information:**	
**Question**	**Response**

Powered by Editorial Manager® and ProduXion Manager® from Aries Systems Corporation

Are you submitting this manuscript to a special series or article collection?	No
**Experimental design and statistics**<br><br>Full details of the experimental design and statistical methods used should be given in the Methods section, as detailed in our Minimum Standards Reporting Checklist. Information essential to interpreting the data presented should be made available in the figure legends.<br><br>Have you included all the information requested in your manuscript?	Yes
**Resources**<br><br>A description of all resources used, including antibodies, cell lines, animals and software tools, with enough information to allow them to be uniquely identified, should be included in the Methods section. Authors are strongly encouraged to cite Research Resource Identifiers (RRIDs) for antibodies, model organisms and tools, where possible.<br><br>Have you included the information requested as detailed in our Minimum Standards Reporting Checklist?	Yes
**Availability of data and materials**<br><br>All datasets and code on which the conclusions of the paper rely must be either included in your submission or deposited in publicly available repositories (where available and ethically appropriate), referencing such data using a unique identifier in the references and in the "Availability of Data and Materials" section of your manuscript.<br><br>Have you have met the above requirement as detailed in our Minimum	Yes

Standards Reporting Checklist?	
GigaScience has policies and guidelines in place for the use of generative AI-writing tools such as ChatGPT. If you have used such writing tools to assist with writing the manuscript this must be declared and cited in the text. Authors should not list AI-writing tools and other AI-assisted technologies as an author or co-author and should acknowledge that they are fully responsible for text generated or refined by AI-writing tools.<p><br><br>A summary of use (particularly in the introduction or among methods) needs to be included at the end of the paper, and the outputs should also be included as a supplementary file hosted in GigaDB or other open repositories. Please read our guidelines for more information. <p><br><br>By submitting to GigaScience, you are aware of the journal's AI-writing tools policy, and if you have declared use of such tools below, you have acknowledged this where appropriate in your manuscript and have made a summary of use and outputs available. <p><br><b>AI-assisted writing tools have been used in the preparation of this manuscript?	No

Manuscript (tex)
Click here to access/download;LaTeX - Main Document (TeX file);main.tex

Placeholder for OUP logo oup.pdf

GigaScience, 2025, 1–7

doi: xx.xxxx/xxxx
Manuscript in Preparation
Technical note

TECHNICAL NOTE

Interactive analysis of single-cell trajectories in 3D space with Cell Journey

Damian Panas[1,2] and Marcin Tabaka[1, 2, *]

[1]International Centre for Translational Eye Research, Skierniewicka 10A, Warsaw, 01-230, Poland and [2]Institute of Physical Chemistry, Polish Academy of Sciences, Kasprzaka 44/52, Warsaw, 01-224, Poland

[*]Corresponding author: mtabaka@ichf.edu.pl

Abstract

The integration of high-throughput single-cell profiling technologies with RNA velocity analysis has enabled the reconstruction of dynamic cellular differentiation trajectories at unprecedented resolution. Despite these advances, current visualization techniques for RNA velocity are predominantly confined to two-dimensional representations, typically employing arrows or streamlines. While effective for depicting simple cellular trajectories, these approaches are insufficient for capturing the complex topologies of multipartite cellular transitions. This limitation highlights the need for advanced three-dimensional visualization tools that can more accurately convey the structure and dynamics of velocity-inferred transitions in single-cell data. Here, we present Cell Journey, an interactive visualization platform specifically developed for three-dimensional analysis and representation of RNA velocity trajectories derived from single-cell datasets. The platform features an intuitive graphical interface supporting both unimodal and multimodal data, accommodates multiple input formats, and provides extensive customization capabilities for trajectory visualization. Cell Journey computes RNA velocity vector fields on a user-defined three-dimensional grid and constructs velocity trajectories using either Euler integration or the fourth-order Runge-Kutta method. The platform enables dynamic exploration of cellular dynamics through interactive visual elements, including streamlines, streamlets, cones, and volumetric plots. Furthermore, it allows users to investigate changes in feature activity along selected paths, facilitating deeper insights into cellular state transitions within complex multimodal single-cell datasets.

Key words: Single-cell multiomics; Developmental trajectory; RNA velocity

Introduction

Single-cell RNA sequencing technologies have enabled the study of cellular differentiation trajectories at unprecedented resolution [1–4]. These methods capture static snapshots of cellular transcriptomic states, with dynamic transitions between states subsequently inferred through computational analysis. Notably, RNA velocity–based approaches have emerged as state-of-the-art tools for uncovering the directionality of cellular transitions [5–21]. In single-cell RNA sequencing data, newly transcribed (unspliced) transcripts retaining intronic sequences can be reliably distinguished from fully spliced, mature transcripts. RNA velocity analysis exploits the quantitative relationship between nascent and mature RNA molecules. By aggregating gene-specific transcriptional dynamics across the transcriptome, RNA velocity enables the prediction of future transcriptional states, effectively forecasting a cell's trajectory in gene expression space over short time scales. RNA velocity has emerged as one of the most influential frameworks for inferring cellular differentiation pathways, disentangling subpopulation kinetics, elucidating lineage relationships, and visualizing dynamic developmental processes. Its introduction has spurred the development of numerous computational methods for trajectory inference [22–28], as well as advanced visualization algorithms that incorporate RNA velocity information to represent cellular dynamics [29–32]. In recent years, we have witnessed the development of multimodal single-cell sequencing technologies that co-profile from the same cell various combinations of genome-wide feature such as transcriptome, chromatin accessibility, histone modifications, and protein epitopes [33–47]. These methods offer an opportunity to understand the temporal relation-

Compiled on: August 12, 2025.
Draft manuscript prepared by the author.

ship between different layers of gene expression regulation and increase the potential of determining cell states. Incorporation of chromatin states switch times to RNA velocity framework improves the accuracy of cell fate prediction compared to velocity estimates from RNA only [10].

The crucial analytical task in RNA velocity analysis is the calculation of cell's transition probability in high-dimensional space and its subsequent projection onto a low-dimensional embedding. The transitions between the cellular states are often visualized as arrows or streamlines on a two-dimensional (2D) cell embedding. However, 2D cell embeddings can result in significant topological misrepresentations [48]. In the context of complex developmental single-cell data, such 2D representations are often insufficient to capture the intricate topologies of multipartite continuous cellular transitions [32]. Cell Journey addresses these limitations by computing and interactively visualizing single-cell velocity-based trajectories in three-dimensional (3D) space (Fig. 1A). Cell Journey computes RNA velocity vectors on a user-defined grid to capture spatially resolved transcriptional dynamics. It then constructs 3D field lines by numerically integrating these vectors, offering a choice between the Euler method and the fourth-order Runge-Kutta algorithm. The Euler method provides a straightforward, stepwise approximation of the cell trajectory, while the fourth-order Runge-Kutta approach delivers enhanced accuracy by accounting for intermediate evaluations within each integration step. This dual-method framework allows researchers to balance computational efficiency and precision when modeling the complex dynamics of cellular state transitions.

Cell Journey is engineered to serve single-cell researchers, irrespective of their computational proficiency, by providing a platform that simplifies the exploration of single-cell datasets in 3D. Its intuitive and accessible graphical user interface (GUI) promotes seamless interaction and efficient data analysis (Fig. 1B). The software employs a state-of-the-art visualization platform to render computed 3D trajectories using graphical aids such as streamlines, streamlets, and cones (Fig. 2A). Additionally, interactive 3D scatterplots enable the dynamic computation and visualization of differentiation trajectories, either initiated from a selected cell or generated across a user-defined regular grid to mitigate the single-cell data sparsity. This functionality enables researchers to interactively explore complex trajectories and efficiently assess multimodal feature activity changes along selected trajectories (Fig. 2B).

Materials and methods

To demonstrate its utility, Cell Journey was applied to visualize RNA velocity-based inferred trajectories from two representative datasets: a unimodal scRNA-seq dataset of mouse pancreatic endocrinogenesis [49] (GSE132188, Fig. 2A) and a multimodal CITE-Seq dataset of human bone marrow mononuclear cell (BMMC) progenitors [50] (GSE128639, Fig. 2B). Unimodal scRNA-seq pacreatic endocrinogenesis was processed with scVelo 0.2.5 [6]. The package was also used to preprocess the data and estimate RNA velocity components. Preprocessing consisted of applying filter_and_normalize function with min_shared_counts parameter equal to 20, and n_top_genes equal to 2000. Next, the moment function was applied with n_pcs and n_neighbors parameters both equal to 30. The UMAP embedding was calculated using Scanpy 1.9.6 [51] with n_components parameter equal to 3. Finally, cell velocities were projected into the UMAP using scVelo's velocity_embedding function. CITE-seq multimodal human bone marrow data was preprocessed with CITE-seq-Count v1.4.5 [46]. The obtained RNA count matrix was preprocessed with Scanpy. The following functions were applied: filter_cells with min_genes equal to 100, filter_genes with min_cells equal to 3, normalize_total and log1p with default parameters, highly_variable_genes with n_top_genes set to 5000. Next, pca and neighbors function were applied with the default parameters, and the three-dimensional UMAP embedding was calculated using the umap function with n_components parameter equal to 3. Finally, RNA velocity was inferred using UnitVelo 0.2.5.2 [7] with N_TOP_GENES parameter equal to 1000 and R2_ADJUST set to False.

Results

Cell Journey is implemented in Python 3.11.7 and leverages a robust ecosystem of libraries to ensure both functionality and user-friendliness. The core dashboard is constructed using Dash, with Dash Mantine Components and Dash Bootstrap Components enhancing the interactivity and aesthetic of the user interface. Interactive visualizations are generated with Plotly, while SciPy provides essential numerical routines, including linear and radial basis interpolation, linear smoothing, and nearest-neighbor lookups. Scikit-learn is utilized for k-means clustering of computed averaged feature activity trends, and Scanpy [51] supports comprehensive processing of single-cell data. The platform also integrates MuData [53] for handling multimodal datasets, with NumPy and Pandas managing array operations and data frames, respectively. Coloraide is employed to interpolate color palettes, ensuring visual consistency throughout the analyses. This cohesive integration of Python-based computational tools enables Cell Journey to deliver scalable and precise analyses of single-cell developmental data.

The platform is engineered to address key tasks in the exploration and visualization of single-cell data (Fig. 1A). It accommodates the upload of diverse dataset formats—including h5ad for single-modality, h5mu for multimodal, and comma-separated CSV dataset files—thus ensuring broad compatibility with various data sources. It enables the visualization of cells embedded in 3D space, where representations can be based on either categorical or continuous feature activity values, such as gene expression, protein epitope levels, or cell cluster annotations. The 3D embeddings of single-cell data can be generated by an arbitrary method, including UMAP [54], FLE [55], or hyperbolic ones such as scPhere [56]. Furthermore, the platform addresses challenges in visualizing low-abundance features and compensates for dropout artifacts inherent in single-cell profiling by rendering feature activity values as partially transparent isosurfaces (volumetric/volume plots) within the 3D embedding (Fig. 2). Furthermore, the volume plot provides a mechanism for interpolating any desired feature by means of a selection of radial basis functions (including Gaussian, linear, quadratic, or multi-quadratic). The smoothing level of the resulting approximation can be accurately controlled via multiple independent parameters, thereby offering a high degree of flexibility in adjusting the level of precision and computational efficiency. These volume plots are fully customizable, allowing users to independently adjust the grid resolution—separate from the grid used for trajectory computation—select appropriate color palettes, and define the range of feature activity values displayed. This level of customization ensures that subtle variations in feature activities are effectively visualized, thereby enhancing the interpretability of low-abundant features like expression levels of transcription factors or surface proteins. Such volume plots become especially important in large-scale 3D visualizations, where small point sizes representing cells and low detection probabilities of features can impede the visualization of activity levels in conventional scatter plots. Extensive customization options for scatterplots are provided, allowing users to define specific scales or color palettes, or to select from an array of built-in options, including those optimized for colorblind accessibility. Additionally, figures can be exported either as static images in raster and vector formats or as interactive visualizations suitable for exploration in a web browser. Finally, Cell Journey offers a flexible interface that supports dynamic zooming of cells and trajectories, with real-time updates following adjustments to visual parameters.

To facilitate the visualization of cellular transitions in three-

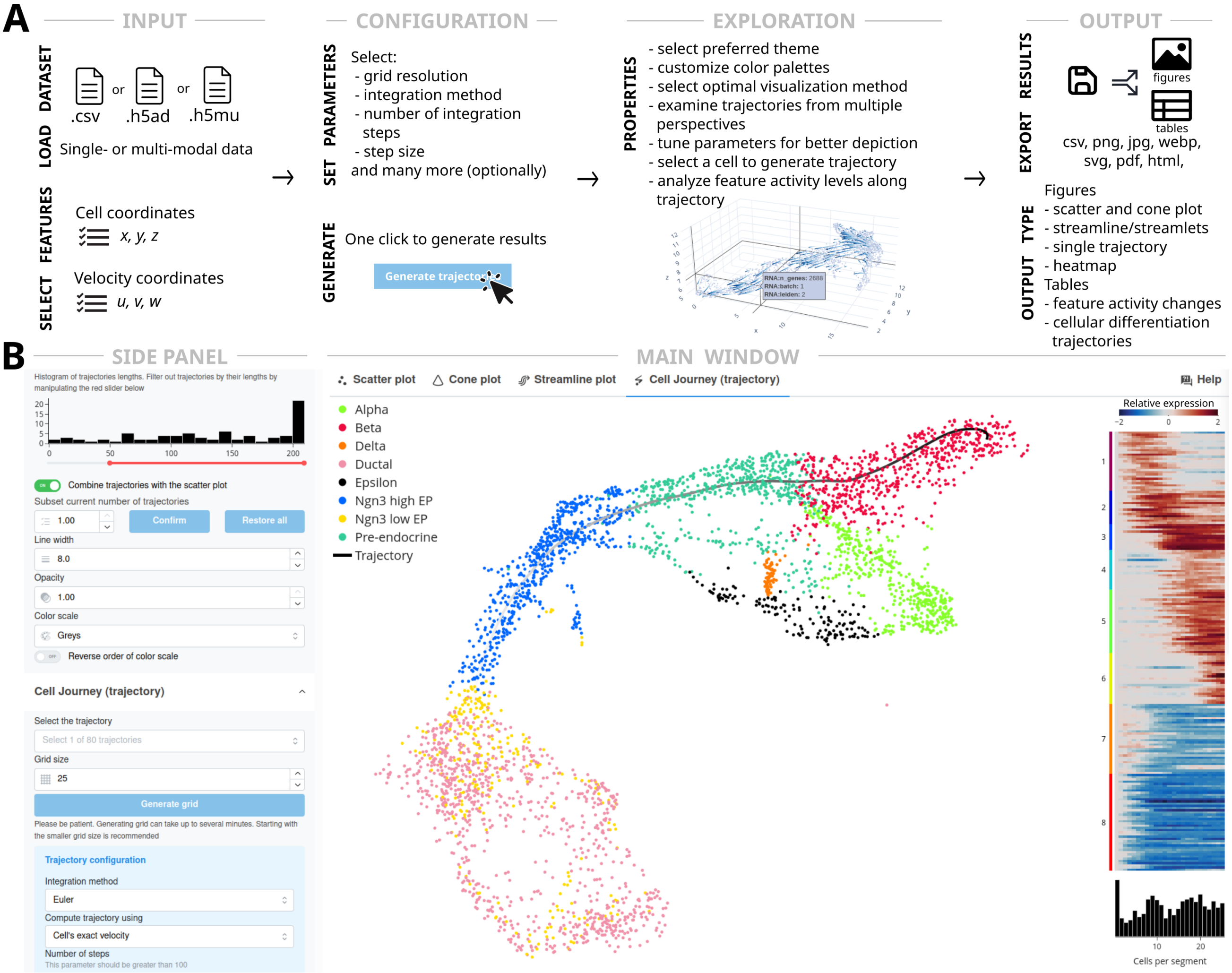

Figure 1. Cell Journey is an interactive tool for visualization and exploratory analysis of single-cell multiomics data and velocity-based trajectories. (A) Overview. Cell Journey accepts as input (left) single-cell datasets in text or hdf5 file format (h5ad or h5mu for uni- or multimodal data). Users in the first step load the data and select the variables of cell coordinates and the components of the velocity vectors. After defining the grid resolution and integration method, Cell Journey computes RNA velocity vector field lines in a 3D cell embedding. Then, the user can explore the visual depiction of the vector field, generate trajectories for a selected cell, and study the modality feature changes along the computed trajectory. Finally, generated figures can be exported in a high-resolution publication-ready format. (B), A part of the interface with a generated trajectory from a selected cell. The software has a main window and a drop-down panel on the left. The fully customizable heatmap shows activities of modality features (e.g. gene expression) along the trajectory.

dimensional single-cell data embeddings, Cell Journey calculates RNA velocity vectors on a regularly spaced grid with user-defined resolution. These vectors are then used to compute 3D field lines through numerical integration, employing either the Euler method or the fourth-order Runge-Kutta algorithm. Researchers can regulate both the step count and the step size of these algorithms to obtain an optimal trajectory length. In particular, implementing parameters such as the scale grid and the difference threshold enables a fine-grained balance between computational speed and the level of detail captured in the integration. Users can flexibly adjust the density of the streamlines, generate streamlets, and customize their attributes — including length, color, transparency, and color gradients depicting direction of trajectories. Users can select specific trajectories for detailed analysis. Moreover, trajectories can be dynamically generated from any selected cell or grid element within the scatterplot. The tool further quantifies changes in uni- or multimodal feature activity levels — such as gene expression profiles or epitope levels — along the selected or generated trajectory. These activity changes are clustered according to their trends and subsequently visualized in an interactive heatmap (Fig. 2B), providing a comprehensive overview of the dynamic cellular processes at genome-wide scale. The sequence of clusters is first determined by grouping the up- and down-regulated averaged profiles and then by ordering their extremal values. This approach yields well-defined temporal groupings of features that exhibit gradual transitions, enabling more precise characterization of dynamic patterns over time.

To evaluate the distinctive capabilities of Cell Journey within the landscape of interactive single-cell data analysis tools, we conducted a comprehensive comparison with a range of existing platforms (Table 1), including ASAP [57], cellxgene [58], Corvo [59], SCope [60], scSVA [61], singlecellVR [62], StarmapVis [63], the UCSC Cell Browser [64], and Vitessce [65]. While several of these tools offer limited support for three-dimensional (3D) visualization of single-cell data, Cell Journey stands out as the most comprehensive platform in terms of functionality and analytical depth. It is the only tool capable of generating and rendering RNA velocity outputs directly in 3D space. Furthermore, it uniquely supports fully interactive computation, visualization, and exploration of cellular trajectories based on RNA velocity, offering an integrated platform for trajectory inference and dynamic state analysis.

We next demonstrate the application of Cell Journey to single-cell transcriptomic and multi-omic datasets, illustrating its capability to resolve complex cellular dynamics through integrated 3D vi-

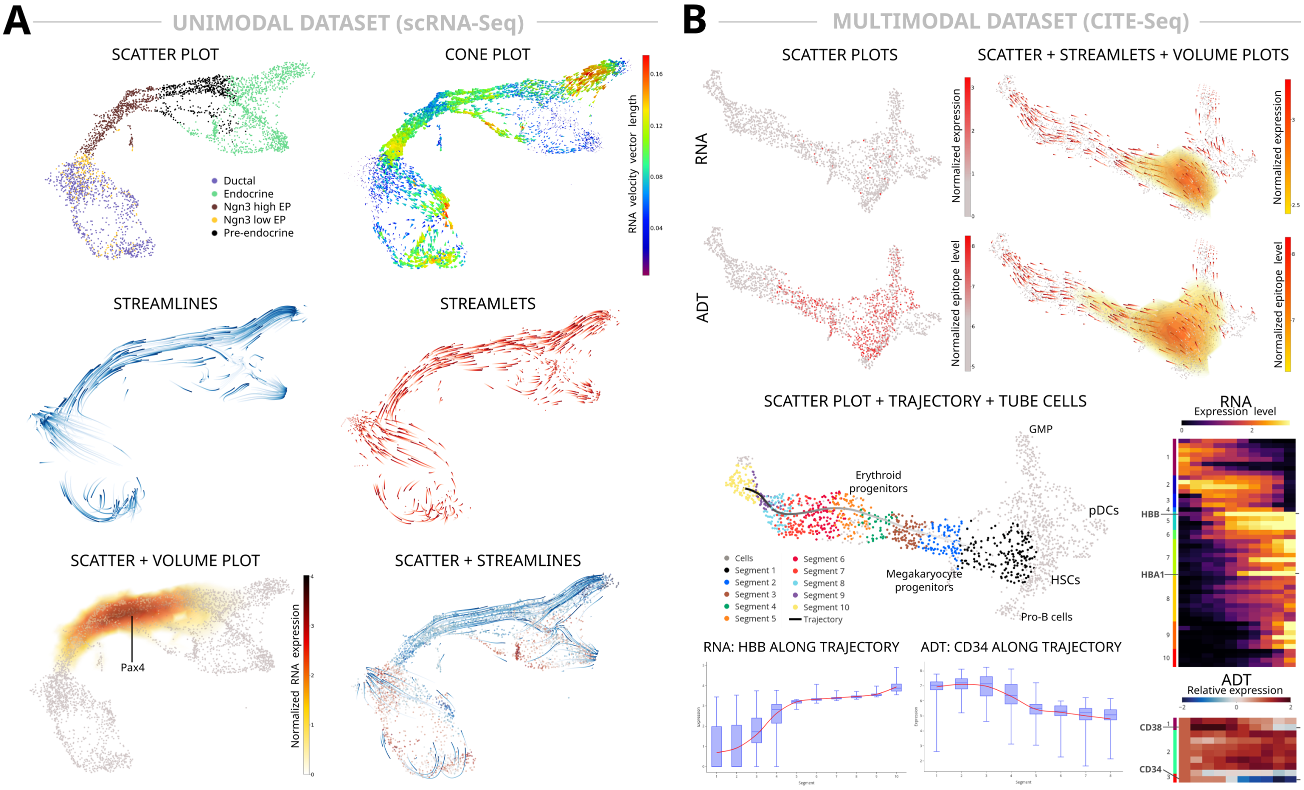

Figure 2. Cell Journey implemented visualizations of 3D single-cell embeddings and RNA velocity vector fields applied to (A) a unimodal scRNA-Seq dataset of pancreatic endocrinogenesis [49], and (B) a multimodal CITE-Seq dataset of human bone marrow mononuclear cell progenitors [52]. Cells in the neighborhood of the generated trajectory are grouped into user-specified segments to calculate the statistics of the feature activities. The interactive heatmap with grouped feature activities along the trajectory displays when clicking on a selected feature the trend of its activity in multiple formats.

sualizations. Figure 2A illustrates the 3D visualization of pancreatic endocrinogenesis, where an example trajectory is generated from a selected endocrine progenitor cell progressing toward Beta cells. Gene expression dynamics along this trajectory are clustered using k-means based on their temporal trends and subsequently visualized via an interactive heatmap. The cone plot, which projects the

Table 1. Comparison of the functionality of Cell Journey with other platforms for interactive exploration and analysis of single-cell data.

		**Cell Journey**	ASAP	cellxgene	Covro	SCope	scSVA	singlecellVR	StarmapVis	UCSC Cell Browser	vitessce
RNA Velocity	Cone plot	+									
	Volume plot	+									
	Streamlines/streamlets	+									
	Combining multiple types of plots	+									
	Trajectory from selected cell	+									
	Feature changes along the trajectory	+									
General	3D view	+	+		+		+	+	+		+
	Multimodality	+			+			+			+
	Docker	+	+	+		+	+	+			
	Example datasets	+	+	+		+		+	+	+	+
Input files	h5		+				+				
	h5ad	+		+	+		+	+		+	
	h5mu	+									
	csv/tsv/txt	+	+				+		+	+	+
	loom		+			+	+	+		+	
	Seurat/rds		+							+	
Processing	Clustering	+	+								+
	Data normalization	+	+			+					
	Scaling	+	+			+					
	Feature/cell filtering	+	+	+	+	+			+	+	

direction of cellular transitions within the 3D embedding, is highly dependent on the precision of detecting unspliced and spliced RNA forms of key genes and consequently displays heterogeneous transition probability patterns. In contrast, streamlines and streamlets, derived from the integration of individual cell velocity vectors, yield more consistent trajectories toward fully differentiated cells. Additionally, the volume plot effectively depicts the expression level of Pax4, a key transcription factor for endocrine development [66], which is transiently expressed in endocrine progenitors [67]. For the visualization of the BMMC CITE-seq dataset in 3D (2B), three modes are employed simultaneously: a scatter plot to display cell positions, streamlets to indicate the direction of cellular transitions, and a volume plot to portray the epitope levels of the CD34 surface marker, which is specific to hematopoietic stem cells (HSCs). Although epitope levels are readily visualized in conventional scatterplots, RNA expression is substantially sparser. Nonetheless, the volume plot derived from RNA modality data accurately reflects its HSC specificity, highlighting the utility of volume plots in delineating low-abundance features. In a subsequent visualization, the same plot orientation is used to generate a trajectory from a selected cell to erythroid progenitor cells. Segments of trajectory-projected cells are marked to compute differentially expressed feature activity levels, which are then grouped according to their trends and visualized in interactive heatmaps. Users can select the modality for analysis — such as RNA or ADT (epitope) levels — and the heatmaps can display either normalized or relative feature activity levels. Notably, the RNA heatmap highlights a pronounced upregulation of hemoglobin subunit beta (HBB) and hemoglobin alpha 1 (HBA1), components of hemoglobin A, along the erythroid lineage. Conversely, the ADT heatmap reveals a systematic decrease in CD34 protein levels from HSCs, alongside a transient upregulation of CD38 levels along the erythroid trajectory. These observations underscore the platform's robust capacity to elucidate dynamic cellular processes across multiple data modalities.

Discussion

Our objective is to facilitate rapid and in-depth exploration of single-cell developmental data in 3D, thereby providing immediate insights and fostering a nuanced understanding of cellular trajectories. Cell Journey is accessible to researchers regardless of computational expertise, featuring a streamlined setup process that requires only a few simple steps and an intuitive, user-friendly interface. A comprehensive help panel, accompanied by FAQs and sample datasets, enables users to quickly familiarize themselves with the tool's functionalities. Designed with extensibility in mind, Cell Journey accommodates the integration of additional analytical modules, thereby supporting increasingly comprehensive analyses. Moreover, the software is optimized for speed, efficiency, and robustness, ensuring reliable performance even when processing large-scale single-cell datasets. Cell Journey is under active development. We plan to extend its functionality by providing new modules for differential trajectory analysis and integration with workspaces for comprehensive single-cell multimodal data analysis. We actively invite user feedback and feature suggestions to drive the continuous enhancement of its functionality, and we are committed to providing regular updates and fostering active community engagement to ensure the tool's ongoing relevance and innovation.

While Cell Journey was originally developed to visualize transitions between cellular states as inferred from RNA velocity analyses [68], its framework is broadly applicable to any method — whether experimental or computational — that delineates cellular state transitions. For instance, the tool can be employed to visualize ground-truth cellular transitions obtained from lineage-tracing experiments, such as Cas9-based cellular ancestry recording [69] or DNA barcoding [70]. These techniques label cells with unique genetic markers, enabling the reconstruction of clonal dynamics and elucidation of lineage relationships through shared mutation patterns or random DNA sequences integrated into the genome. Furthermore, Cell Journey can be potentially used to integrate and visualize trajectories from transition probabilities between cells computationally inferred using optimal transport algorithms applied to time-course single-cell transcriptomic data [3] or multimodal or spatial datasets [71]. Finally, we foresee that Cell Journey will be instrumental in visualizing putative cellular transitions within 3D spatial transcriptomics datasets [72], particularly when combined with emerging methodologies for spatial RNA velocity analysis [73–75]. This versatility underscores the potential of Cell Journey to facilitate a comprehensive understanding of cellular dynamics across diverse experimental and computational platforms.

Availability of Source Code and Requirements

- Project name: Cell Journey
- Project homepage: https://github.com/TabakaLab/CellJourney
- Documentation: https://tabakalab.github.io/CellJourney
- Operating system(s): Platform independent
- Programming languages: Python
- Other requirements: Python 3.11.7, Dash 2.14, Plotly 5.18
- License: MIT license

Acknowledgements and funding

We thank members of the Computational Genomics Group for their discussions. D.P. and M.T. are supported by the International Centre for Translational Eye Research (FENG.02.01-IP.05-T005/23) project, which is carried out within the International Research Agendas Program of the Foundation for Polish Science, co-financed by the European Union under the European Regional Development Fund; and grant funded by National Science Center, Poland: the Sonata Bis 12 grant 2022/46/E/NZ2/0037 (M.T.).

Author's Contributions

M.T. conceived and supervised the project. M.T. and D.P. designed and developed Cell Journey. D.P. implemented Cell Journey and wrote its documentation. M.T. and D.P. wrote the manuscript.

Competing Interests

The authors declare no competing interests.

Code availability

Cell Journey is under the MIT License. The software, documentation, tutorials, example datasets, and animated demos can be found at https://TabakaLab.github.io/CellJourney.

References

1. Trapnell C, Cacchiarelli D, Grimsby J, Pokharel P, Li S, Morse M, et al. The dynamics and regulators of cell fate decisions are revealed by pseudotemporal ordering of single cells. Nature Biotechnology 2014;32(4):381–386.
2. Farrell JA, Wang Y, Riesenfeld SJ, Shekhar K, Regev A, Schier AF. Single-cell reconstruction of developmental trajectories during zebrafish embryogenesis. Science 2018;360(6392):eaar3131.
3. Schiebinger G, Shu J, Tabaka M, Cleary B, Subramanian V,

Solomon A, et al. Optimal-Transport Analysis of Single-Cell Gene Expression Identifies Developmental Trajectories in Reprogramming. Cell 2019;176(4):928–943.e22.
4. La Manno G, Siletti K, Furlan A, Gyllborg D, Vinsland E, Mossi Albiach A, et al. Molecular architecture of the developing mouse brain. Nature 2021;596(7870):92–96.
5. La Manno G, Soldatov R, Zeisel A, Braun E, Hochgerner H, Petukhov V, et al. RNA velocity of single cells. Nature 2018;560(7719):494–498.
6. Berker V, Lange M, Paidli S, Wolf FA, Theis FJ. Generalizing RNA velocity to transient cell states through dynamical modeling. Nature Biotechnology 2020;38(12):1408–1414.
7. Gao M, Qiao C, Huang Y. UniTVelo: temporally unified RNA velocity reinforces single-cell trajectory inference. Nature Communications 2022;13(1):6586.
8. Lange M, Bergen V, Klein M, Setty M, Reuter B, Bakhti M, et al. CellRank for directed single-cell fate mapping. Nature Methods 2022;19(2):159–170.
9. Qiu X, Zhang Y, Martin-Rufino JD, Weng C, Hosseinzadeh S, Yang D, et al. Mapping transcriptomic vector fields of single cells. Cell 2022;185(4):690–711.e45.
10. Li C, Virgilio MC, Collins KL, Welch JD. Multi-omic single-cell velocity models epigenome–transcriptome interactions and improves cell fate prediction. Nature Biotechnology 2023;41(3):387–398.
11. Cui H, Maan H, Vladoiu MC, Zhang J, Taylor MD, Wang B. DeepVelo: deep learning extends RNA velocity to multi-lineage systems with cell-specific kinetics. Genome Biology 2024;25(1):27.
12. Gayoso A, Weiler P, Lotfollahi M, Klein D, Hong J, Streets A, et al. Deep generative modeling of transcriptional dynamics for RNA velocity analysis in single cells. Nature Methods 2024;21(1):50–59.
13. Li J, Pan X, Yuan Y, Shen HB. TFvelo: gene regulation inspired RNA velocity estimation. Nature Communications 2024;15(1):1387.
14. Li S, Zhang P, Chen W, Ye L, Brannan KW, Le NT, et al. A relay velocity model infers cell-dependent RNA velocity. Nature Biotechnology 2024;42(1):99–108.
15. Peng Q, Qiu X, Li T. Storm: Incorporating transient stochastic dynamics to infer the RNA velocity with metabolic labeling information. PLOS Computational Biology 2024 11;20(11):1–38.
16. Wang K, Hou L, Wang X, Zhai X, Lu Z, Zi Z, et al. PhyloVelo enhances transcriptomic velocity field mapping using monotonically expressed genes. Nature Biotechnology 2024;42(5):778–789.
17. Ge M, Miao J, Qi J, Zhou X, Lin Z. TIVelo: RNA velocity estimation leveraging cluster-level trajectory inference. Nature Communications 2025;16(1):6258.
18. Aivazidis A, Memi F, Kleshchevnikov V, Er S, Clarke B, Stegle O, et al. Cell2fate infers RNA velocity modules to improve cell fate prediction. Nature Methods 2025;p. 1–10.
19. Wang W, Hu Z, Weiler P, Mayes S, Lange M, Wang J, et al. RegVelo: gene-regulatory-informed dynamics of single cells. bioRxiv 2024;.
20. Chen Y, Zhang Y, Gan J, Ni K, Chen M, Bahar I, et al. GraphVelo allows for accurate inference of multimodal velocities and molecular mechanisms for single cells. bioRxiv 2025;.
21. Li J, Wang Z, Shen HB, Yuan Y. TSvelo: Comprehensive RNA velocity inference by jointly modeling Transcription and Splicing. bioRxiv 2024;.
22. Wolf FA, Hamey FK, Plass M, Solana J, Dahlin JS, Göttgens B, et al. PAGA: graph abstraction reconciles clustering with trajectory inference through a topology preserving map of single cells. Genome Biology 2019;20(1):59.
23. Schwabe D, Formichetti S, Junker JP, Falcke M, Rajewsky N. The transcriptome dynamics of single cells during the cell cycle. Molecular Systems Biology 2020;16(11):e9946.
24. Weng G, Kim J, Won KJ. VeTra: a tool for trajectory inference based on RNA velocity. Bioinformatics 2021;37(20):3509–3513.
25. Zhang Z, Zhang X. Inference of high-resolution trajectories in single-cell RNA-seq data by using RNA velocity. Cell Reports Methods 2021;1(6).
26. Gupta R, Cerletti D, Gut G, Oxenius A, Claassen M. Simulation-based inference of differentiation trajectories from RNA velocity fields. Cell Reports Methods 2022;2(12).
27. Lange M, Bergen V, Klein M, Setty M, Reuter B, Bakhti M, et al. CellRank for directed single-cell fate mapping. Nature Methods 2022;19(2):159–170.
28. Weiler P, Lange M, Klein M, Pe'er D, Theis F. CellRank 2: unified fate mapping in multiview single-cell data. Nature Methods 2024;21(7):1196–1205.
29. Atta L, Sahoo A, Fan J. VeloViz: RNA velocity-informed embeddings for visualizing cellular trajectories. Bioinformatics 2022;38(2):391–396.
30. Xia L, Lee C, Li JJ. Statistical method scDEED for detecting dubious 2D single-cell embeddings and optimizing t-SNE and UMAP hyperparameters. Nature Communications 2024;15(1):1753.
31. Sun ED, Ma R, Zou J. Dynamic visualization of high-dimensional data. Nature Computational Science 2023;3(1):86–100.
32. Rutkowski P, Tabaka M. Ocelli: an open-source tool for the analysis and visualization of developmental multimodal single-cell data. NAR Genomics and Bioinformatics 2025;7(2):lqaf040.
33. Cao J, Cusanovich DA, Ramani V, Aghamirzaie D, Pliner HA, Hill AJ, et al. Joint profiling of chromatin accessibility and gene expression in thousands of single cells. Science 2018;361(6409):1380–1385.
34. Zhu C, Yu M, Huang H, Juric I, Abnousi A, Hu R, et al. An ultra high-throughput method for single-cell joint analysis of open chromatin and transcriptome. Nature Structural & Molecular Biology 2019;26(11):1063–1070.
35. Ma S, Zhang B, LaFave LM, Earl AS, Chiang Z, Hu Y, et al. Chromatin potential identified by shared single-cell profiling of RNA and chromatin. Cell 2020;183(4):1103–1116.
36. Chen S, Lake BB, Zhang K. High-throughput sequencing of the transcriptome and chromatin accessibility in the same cell. Nature Biotechnology 2019;37(12):1452–1457.
37. Hunt KV, Burnard SM, Roper EA, Bond DR, Dun MD, Verrills NM, et al. scTEM-seq: Single-cell analysis of transposable element methylation to link global epigenetic heterogeneity with transcriptional programs. Scientific Reports 2022;12(1):5776.
38. Zhu C, Zhang Y, Li YE, Lucero J, Behrens MM, Ren B. Joint profiling of histone modifications and transcriptome in single cells from mouse brain. Nature Methods 2021;18(3):283–292.
39. Pan L, Ku WL, Tang Q, Cao Y, Zhao K. scPCOR-seq enables co-profiling of chromatin occupancy and RNAs in single cells. Communications Biology 2022;5(1):678.
40. Tedesco M, Giannese F, Lazarević D, Giansanti V, Rosano D, Monzani S, et al. Chromatin Velocity reveals epigenetic dynamics by single-cell profiling of heterochromatin and euchromatin. Nature Biotechnology 2022;40(2):235–244.
41. Bartosovic M, Kabbe M, Castelo-Branco G. Single-cell CUT&Tag profiles histone modifications and transcription factors in complex tissues. Nature Biotechnology 2021;39(7):825–835.
42. Gopalan S, Wang Y, Harper NW, Garber M, Fazzio TG. Simultaneous profiling of multiple chromatin proteins in the same cells. Molecular Cell 2021;81(22):4736–4746.
43. Stuart T, Hao S, Zhang B, Mekerishvili L, Landau DA, Maniatis S, et al. Nanobody-tethered transposition enables multifactorial chromatin profiling at single-cell resolution. Nature Biotechnology 2022;p. 1–7.
44. Bartosovic M, Castelo-Branco G. Multimodal chromatin profiling using nanobody-based single-cell CUT&Tag. Nature Biotechnology 2022;p. 1–12.

45. Yeung J, Florescu M, Zeller P, de Barbanson BA, Wellenstein MD, van Oudenaarden A. scChIX-seq infers dynamic relationships between histone modifications in single cells. Nature Biotechnology 2023;p. 1–11.
46. Stoeckius M, Hafemeister C, Stephenson W, Houck-Loomis B, Chattopadhyay PK, Swerdlow H, et al. Simultaneous epitope and transcriptome measurement in single cells. Nature Methods 2017;14(9):865–868.
47. Mimitou EP, Lareau CA, Chen KY, Zorzetto-Fernandes AL, Hao Y, Takeshima Y, et al. Scalable, multimodal profiling of chromatin accessibility, gene expression and protein levels in single cells. Nature Biotechnology 2021;39(10):1246–1258.
48. Wang S, Sontag ED, Lauffenburger DA. What cannot be seen correctly in 2D visualizations of single-cell 'omics data? Cell Systems 2023;14(9):723–731.
49. Bastidas-Ponce A, Tritschler S, Dony L, Scheibner K, Tarquis-Medina M, Salinno C, et al. Comprehensive single cell mRNA profiling reveals a detailed roadmap for pancreatic endocrinogenesis. Development 2019 06;146(12):dev173849.
50. Stuart T, Butler A, Hoffman P, Hafemeister C, Papalexi E, Mauck WM, et al. Comprehensive integration of single-cell data. Cell 2019;177(7):1888–1902.
51. Wolf FA, Angerer P, Theis FJ. SCANPY: large-scale single-cell gene expression data analysis. Genome Biology 2018;19(1):15.
52. Hao Y, Hao S, Andersen-Nissen E, Mauck WMr, Zheng S, Butler A, et al. Integrated analysis of multimodal single-cell data. Cell 2021;184(13):3573–3587.e29.
53. Bredikhin D, Kats I, Stegle O. MUON: multimodal omics analysis framework. Genome Biology 2022;23(1):42.
54. McInnes L, Healy J, Melville J. Umap: Uniform manifold approximation and projection for dimension reduction. arXiv preprint arXiv:180203426 2018;.
55. Jacomy M, Venturini T, Heymann S, Bastian M. ForceAtlas2, a continuous graph layout algorithm for handy network visualization designed for the Gephi software. PLOS ONE 2014;9(6):e98679.
56. Ding J, Regev A. Deep generative model embedding of single-cell RNA-Seq profiles on hyperspheres and hyperbolic spaces. Nature Communications 2021;12(1):2554.
57. Gardeux V, David FPA, Shajkofci A, Schwalie PC, Deplancke B. ASAP: a web-based platform for the analysis and interactive visualization of single-cell RNA-seq data. Bioinformatics 2017;33(19):3123–3125.
58. Program CCS, Abdulla S, Aevermann B, Assis P, Badajoz S, Bell SM, et al. CZ CELLxGENE Discover: a single-cell data platform for scalable exploration, analysis and modeling of aggregated data. Nucleic Acids Research 2024 11;53(D1):D886–D900.
59. Hyman L, Sbalzarini IF, Quake S, Günther U, Corvo: Visualizing CellxGene Single-Cell Datasets in Virtual Reality; 2022.
60. Davie K, Janssens J, Koldere D, De Waegeneer M, Pech U, Kreft L, et al. A Single-Cell Transcriptome Atlas of the Aging Drosophila Brain. Cell 2018;174(4):982–998.e20.
61. Tabaka M, Gould J, Regev A. scSVA: an interactive tool for big data visualization and exploration in single-cell omics. bioRxiv 2019;.
62. Stein DF, Chen H, Vinyard ME, Qin Q, Combs RD, Zhang Q, et al. singlecellVR: Interactive Visualization of Single-Cell Data in Virtual Reality. Frontiers in Genetics 2021;12.
63. Ma S, Fang X, Yao Y, Li J, Morgan DC, Xia Y, et al. StarmapVis: An interactive and narrative visualisation tool for single-cell and spatial data. Computational and Structural Biotechnology Journal 2023;21:1598–1605.
64. Speir ML, Bhaduri A, Markov NS, Moreno P, Nowakowski TJ, Papatheodorou I, et al. UCSC Cell Browser: visualize your single-cell data. Bioinformatics 2021 07;37(23):4578–4580.
65. Keller MS, Gold I, McCallum C, Manz T, Kharchenko PV, Gehlenborg N. Vitessce: integrative visualization of multimodal and spatially resolved single-cell data. Nature Methods 2025;22(1):63–67.
66. Collombat P, Mansouri A, Hecksher-Sørensen J, Serup P, Krull J, Gradwohl G, et al. Opposing actions of Arx and Pax4 in endocrine pancreas development. Genes & Development 2003;17(20):2591–2603.
67. Yu XX, Qiu WL, Yang L, Wang YC, He MY, Wang D, et al. Sequential progenitor states mark the generation of pancreatic endocrine lineages in mice and humans. Cell Research 2021;31(8):886–903.
68. Wang Y, Li J, Zha H, Liu S, Huang D, Fu L, et al. Paradigms, innovations, and biological applications of RNA velocity: a comprehensive review. Briefings in Bioinformatics 2025;26(4):bbaf339.
69. McKenna A, Findlay GM, Gagnon JA, Horwitz MS, Schier AF, Shendure J. Whole-organism lineage tracing by combinatorial and cumulative genome editing. Science 2016;353(6298):aaf7907.
70. Gutierrez C, Al'Khafaji A, Brenner E, Johnson K, Gohil S, Lin Z, et al. Multifunctional barcoding with ClonMapper enables high-resolution study of clonal dynamics during tumor evolution and treatment. DOI: https://doi org/101038/s43018-021-00222-8 2021;2(7):758–772.
71. Klein D, Palla G, Lange M, Klein M, Piran Z, Gander M, et al. Mapping cells through time and space with moscot. Nature 2025;638(8052):1065–1075.
72. Schott M, León-Periñán D, Splendiani E, Strenger L, Licha JR, Pentimalli TM, et al. Open-ST: High-resolution spatial transcriptomics in 3D. Cell 2024;187(15):3953–3972.
73. Abdelaal T, Grossouw LM, Pasterkamp RJ, Lelieveldt BP, Reinders MJ, Mahfouz A. SIRV: spatial inference of RNA velocity at the single-cell resolution. NAR Genomics and Bioinformatics 2024;6(3):lqae100.
74. Zhou P, Bocci F, Li T, Nie Q. Spatial transition tensor of single cells. Nature Methods 2024;21(6):1053–1062.
75. Gu Y, Liu J, Lee KH, Li C, Lu L, Moline J, et al. Topological velocity inference from spatial transcriptomic data. Nature Biotechnology 2025;p. 1–12.

Manuscript (pdf format)
Click here to access/download;Manuscript;Cell_Journey_Manuscript.pdf

Placeholder for OUP logo oup.pdf

GigaScience, 2025, 1–7

doi: xx.xxxx/xxxx
Manuscript in Preparation
Technical note

TECHNICAL NOTE

Interactive analysis of single-cell trajectories in 3D space with Cell Journey

Damian Panas[1,2] and Marcin Tabaka[1, 2, *]

[1]International Centre for Translational Eye Research, Skierniewicka 10A, Warsaw, 01-230, Poland and [2]Institute of Physical Chemistry, Polish Academy of Sciences, Kasprzaka 44/52, Warsaw, 01-224, Poland

[*]Corresponding author: mtabaka@ichf.edu.pl

Abstract

The integration of high-throughput single-cell profiling technologies with RNA velocity analysis has enabled the reconstruction of dynamic cellular differentiation trajectories at unprecedented resolution. Despite these advances, current visualization techniques for RNA velocity are predominantly confined to two-dimensional representations, typically employing arrows or streamlines. While effective for depicting simple cellular trajectories, these approaches are insufficient for capturing the complex topologies of multipartite cellular transitions. This limitation highlights the need for advanced three-dimensional visualization tools that can more accurately convey the structure and dynamics of velocity-inferred transitions in single-cell data. Here, we present Cell Journey, an interactive visualization platform specifically developed for three-dimensional analysis and representation of RNA velocity trajectories derived from single-cell datasets. The platform features an intuitive graphical interface supporting both unimodal and multimodal data, accommodates multiple input formats, and provides extensive customization capabilities for trajectory visualization. Cell Journey computes RNA velocity vector fields on a user-defined three-dimensional grid and constructs velocity trajectories using either Euler integration or the fourth-order Runge-Kutta method. The platform enables dynamic exploration of cellular dynamics through interactive visual elements, including streamlines, streamlets, cones, and volumetric plots. Furthermore, it allows users to investigate changes in feature activity along selected paths, facilitating deeper insights into cellular state transitions within complex multimodal single-cell datasets.

Key words: Single-cell multiomics; Developmental trajectory; RNA velocity

Introduction

Single-cell RNA sequencing technologies have enabled the study of cellular differentiation trajectories at unprecedented resolution [1–4]. These methods capture static snapshots of cellular transcriptomic states, with dynamic transitions between states subsequently inferred through computational analysis. Notably, RNA velocity–based approaches have emerged as state-of-the-art tools for uncovering the directionality of cellular transitions [5–21]. In single-cell RNA sequencing data, newly transcribed (unspliced) transcripts retaining intronic sequences can be reliably distinguished from fully spliced, mature transcripts. RNA velocity analysis exploits the quantitative relationship between nascent and mature RNA molecules. By aggregating gene-specific transcriptional dynamics across the transcriptome, RNA velocity enables the prediction of future transcriptional states, effectively forecasting a cell's trajectory in gene expression space over short time scales. RNA velocity has emerged as one of the most influential frameworks for inferring cellular differentiation pathways, disentangling subpopulation kinetics, elucidating lineage relationships, and visualizing dynamic developmental processes. Its introduction has spurred the development of numerous computational methods for trajectory inference [22–28], as well as advanced visualization algorithms that incorporate RNA velocity information to represent cellular dynamics [29–32]. In recent years, we have witnessed the development of multimodal single-cell sequencing technologies that co-profile from the same cell various combinations of genome-wide feature such as transcriptome, chromatin accessibility, histone modifications, and protein epitopes [33–47]. These methods offer an opportunity to understand the temporal relation-

Compiled on: August 12, 2025.
Draft manuscript prepared by the author.

ship between different layers of gene expression regulation and increase the potential of determining cell states. Incorporation of chromatin states switch times to RNA velocity framework improves the accuracy of cell fate prediction compared to velocity estimates from RNA only [10].

The crucial analytical task in RNA velocity analysis is the calculation of cell's transition probability in high-dimensional space and its subsequent projection onto a low-dimensional embedding. The transitions between the cellular states are often visualized as arrows or streamlines on a two-dimensional (2D) cell embedding. However, 2D cell embeddings can result in significant topological misrepresentations [48]. In the context of complex developmental single-cell data, such 2D representations are often insufficient to capture the intricate topologies of multipartite continuous cellular transitions [32]. Cell Journey addresses these limitations by computing and interactively visualizing single-cell velocity-based trajectories in three-dimensional (3D) space (Fig. 1A). Cell Journey computes RNA velocity vectors on a user-defined grid to capture spatially resolved transcriptional dynamics. It then constructs 3D field lines by numerically integrating these vectors, offering a choice between the Euler method and the fourth-order Runge-Kutta algorithm. The Euler method provides a straightforward, stepwise approximation of the cell trajectory, while the fourth-order Runge-Kutta approach delivers enhanced accuracy by accounting for intermediate evaluations within each integration step. This dual-method framework allows researchers to balance computational efficiency and precision when modeling the complex dynamics of cellular state transitions.

Cell Journey is engineered to serve single-cell researchers, irrespective of their computational proficiency, by providing a platform that simplifies the exploration of single-cell datasets in 3D. Its intuitive and accessible graphical user interface (GUI) promotes seamless interaction and efficient data analysis (Fig. 1B). The software employs a state-of-the-art visualization platform to render computed 3D trajectories using graphical aids such as streamlines, streamlets, and cones (Fig. 2A). Additionally, interactive 3D scatterplots enable the dynamic computation and visualization of differentiation trajectories, either initiated from a selected cell or generated across a user-defined regular grid to mitigate the single-cell data sparsity. This functionality enables researchers to interactively explore complex trajectories and efficiently assess multimodal feature activity changes along selected trajectories (Fig. 2B).

Materials and methods

To demonstrate its utility, Cell Journey was applied to visualize RNA velocity-based inferred trajectories from two representative datasets: a unimodal scRNA-seq dataset of mouse pancreatic endocrinogenesis [49] (GSE132188, Fig. 2A) and a multimodal CITE-Seq dataset of human bone marrow mononuclear cell (BMMC) progenitors [50] (GSE128639, Fig. 2B). Unimodal scRNA-seq pacreatic endocrinogenesis was processed with scVelo 0.2.5 [6]. The package was also used to preprocess the data and estimate RNA velocity components. Preprocessing consisted of applying filter_and_normalize function with min_shared_counts parameter equal to 20, and n_top_genes equal to 2000. Next, the moment function was applied with n_pcs and n_neighbors parameters both equal to 30. The UMAP embedding was calculated using Scanpy 1.9.6 [51] with n_components parameter equal to 3. Finally, cell velocities were projected into the UMAP using scVelo's velocity_embedding function. CITE-seq multimodal human bone marrow data was preprocessed with CITE-seq-Count v1.4.5 [46]. The obtained RNA count matrix was preprocessed with Scanpy. The following functions were applied: filter_cells with min_genes equal to 100, filter_genes with min_cells equal to 3, normalize_total and log1p with default parameters, highly_variable_genes with n_top_genes set to 5000. Next, pca and neighbors function were applied with the default parameters, and the three-dimensional UMAP embedding was calculated using the umap function with n_components parameter equal to 3. Finally, RNA velocity was inferred using UnitVelo 0.2.5.2 [7] with N_TOP_GENES parameter equal to 1000 and R2_ADJUST set to False.

Results

Cell Journey is implemented in Python 3.11.7 and leverages a robust ecosystem of libraries to ensure both functionality and user-friendliness. The core dashboard is constructed using Dash, with Dash Mantine Components and Dash Bootstrap Components enhancing the interactivity and aesthetic of the user interface. Interactive visualizations are generated with Plotly, while SciPy provides essential numerical routines, including linear and radial basis interpolation, linear smoothing, and nearest-neighbor lookups. Scikit-learn is utilized for k-means clustering of computed averaged feature activity trends, and Scanpy [51] supports comprehensive processing of single-cell data. The platform also integrates MuData [53] for handling multimodal datasets, with NumPy and Pandas managing array operations and data frames, respectively. Coloraide is employed to interpolate color palettes, ensuring visual consistency throughout the analyses. This cohesive integration of Python-based computational tools enables Cell Journey to deliver scalable and precise analyses of single-cell developmental data.

The platform is engineered to address key tasks in the exploration and visualization of single-cell data (Fig. 1A). It accommodates the upload of diverse dataset formats—including h5ad for single-modality, h5mu for multimodal, and comma-separated CSV dataset files—thus ensuring broad compatibility with various data sources. It enables the visualization of cells embedded in 3D space, where representations can be based on either categorical or continuous feature activity values, such as gene expression, protein epitope levels, or cell cluster annotations. The 3D embeddings of single-cell data can be generated by an arbitrary method, including UMAP [54], FLE [55], or hyperbolic ones such as scPhere [56]. Furthermore, the platform addresses challenges in visualizing low-abundance features and compensates for dropout artifacts inherent in single-cell profiling by rendering feature activity values as partially transparent isosurfaces (volumetric/volume plots) within the 3D embedding (Fig. 2). Furthermore, the volume plot provides a mechanism for interpolating any desired feature by means of a selection of radial basis functions (including Gaussian, linear, quadratic, or multi-quadratic). The smoothing level of the resulting approximation can be accurately controlled via multiple independent parameters, thereby offering a high degree of flexibility in adjusting the level of precision and computational efficiency. These volume plots are fully customizable, allowing users to independently adjust the grid resolution—separate from the grid used for trajectory computation—select appropriate color palettes, and define the range of feature activity values displayed. This level of customization ensures that subtle variations in feature activities are effectively visualized, thereby enhancing the interpretability of low-abundant features like expression levels of transcription factors or surface proteins. Such volume plots become especially important in large-scale 3D visualizations, where small point sizes representing cells and low detection probabilities of features can impede the visualization of activity levels in conventional scatter plots. Extensive customization options for scatterplots are provided, allowing users to define specific scales or color palettes, or to select from an array of built-in options, including those optimized for colorblind accessibility. Additionally, figures can be exported either as static images in raster and vector formats or as interactive visualizations suitable for exploration in a web browser. Finally, Cell Journey offers a flexible interface that supports dynamic zooming of cells and trajectories, with real-time updates following adjustments to visual parameters.

To facilitate the visualization of cellular transitions in three-

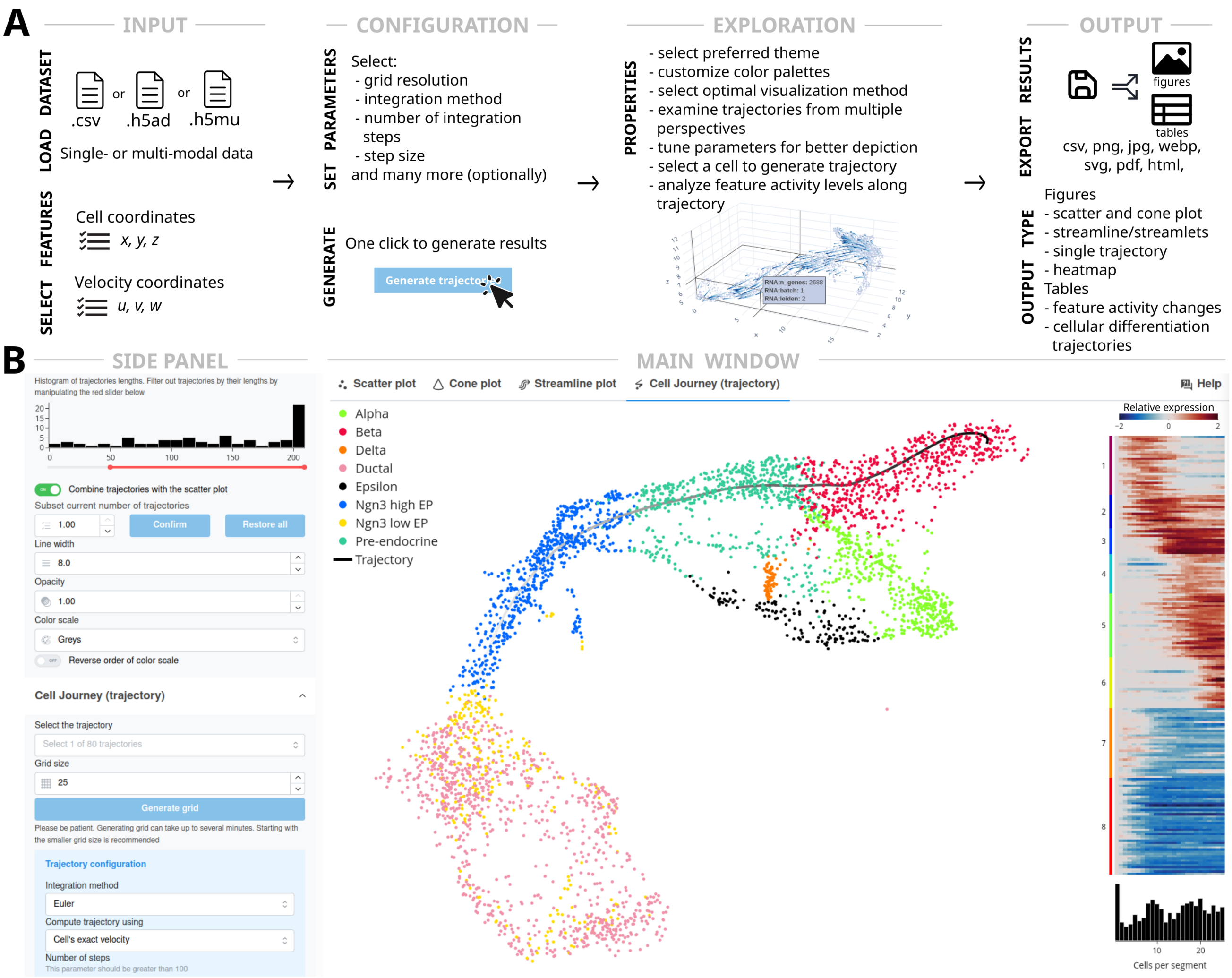

Figure 1. Cell Journey is an interactive tool for visualization and exploratory analysis of single-cell multiomics data and velocity-based trajectories. (A) Overview. Cell Journey accepts as input (left) single-cell datasets in text or hdf5 file format (h5ad or h5mu for uni- or multimodal data). Users in the first step load the data and select the variables of cell coordinates and the components of the velocity vectors. After defining the grid resolution and integration method, Cell Journey computes RNA velocity vector field lines in a 3D cell embedding. Then, the user can explore the visual depiction of the vector field, generate trajectories for a selected cell, and study the modality feature changes along the computed trajectory. Finally, generated figures can be exported in a high-resolution publication-ready format. (B), A part of the interface with a generated trajectory from a selected cell. The software has a main window and a drop-down panel on the left. The fully customizable heatmap shows activities of modality features (e.g. gene expression) along the trajectory.

dimensional single-cell data embeddings, Cell Journey calculates RNA velocity vectors on a regularly spaced grid with user-defined resolution. These vectors are then used to compute 3D field lines through numerical integration, employing either the Euler method or the fourth-order Runge-Kutta algorithm. Researchers can regulate both the step count and the step size of these algorithms to obtain an optimal trajectory length. In particular, implementing parameters such as the scale grid and the difference threshold enables a fine-grained balance between computational speed and the level of detail captured in the integration. Users can flexibly adjust the density of the streamlines, generate streamlets, and customize their attributes — including length, color, transparency, and color gradients depicting direction of trajectories. Users can select specific trajectories for detailed analysis. Moreover, trajectories can be dynamically generated from any selected cell or grid element within the scatterplot. The tool further quantifies changes in uni- or multimodal feature activity levels — such as gene expression profiles or epitope levels — along the selected or generated trajectory. These activity changes are clustered according to their trends and subsequently visualized in an interactive heatmap (Fig. 2B), providing a comprehensive overview of the dynamic cellular processes at genome-wide scale. The sequence of clusters is first determined by grouping the up- and down-regulated averaged profiles and then by ordering their extremal values. This approach yields well-defined temporal groupings of features that exhibit gradual transitions, enabling more precise characterization of dynamic patterns over time.

To evaluate the distinctive capabilities of Cell Journey within the landscape of interactive single-cell data analysis tools, we conducted a comprehensive comparison with a range of existing platforms (Table 1), including ASAP [57], cellxgene [58], Corvo [59], SCope [60], scSVA [61], singlecellVR [62], StarmapVis [63], the UCSC Cell Browser [64], and Vitessce [65]. While several of these tools offer limited support for three-dimensional (3D) visualization of single-cell data, Cell Journey stands out as the most comprehensive platform in terms of functionality and analytical depth. It is the only tool capable of generating and rendering RNA velocity outputs directly in 3D space. Furthermore, it uniquely supports fully interactive computation, visualization, and exploration of cellular trajectories based on RNA velocity, offering an integrated platform for trajectory inference and dynamic state analysis.

We next demonstrate the application of Cell Journey to single-cell transcriptomic and multi-omic datasets, illustrating its capability to resolve complex cellular dynamics through integrated 3D vi-

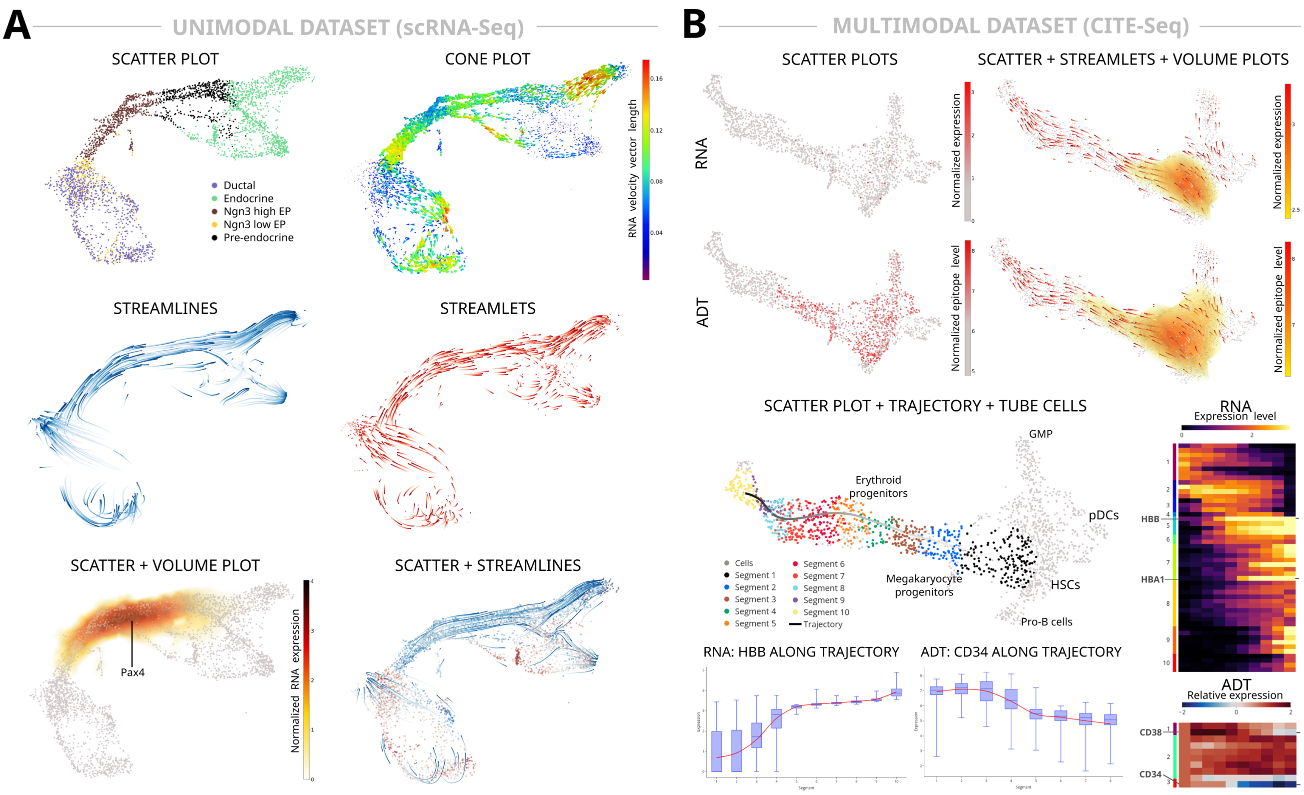

Figure 2. Cell Journey implemented visualizations of 3D single-cell embeddings and RNA velocity vector fields applied to (A) a unimodal scRNA-Seq dataset of pancreatic endocrinogenesis [49], and (B) a multimodal CITE-Seq dataset of human bone marrow mononuclear cell progenitors [52]. Cells in the neighborhood of the generated trajectory are grouped into user-specified segments to calculate the statistics of the feature activities. The interactive heatmap with grouped feature activities along the trajectory displays when clicking on a selected feature the trend of its activity in multiple formats.

sualizations. Figure 2A illustrates the 3D visualization of pancreatic endocrinogenesis, where an example trajectory is generated from a selected endocrine progenitor cell progressing toward Beta cells. Gene expression dynamics along this trajectory are clustered using k-means based on their temporal trends and subsequently visualized via an interactive heatmap. The cone plot, which projects the

Table 1. Comparison of the functionality of Cell Journey with other platforms for interactive exploration and analysis of single-cell data.

		**Cell Journey**	ASAP	cellxgene	Covro	SCope	scSVA	singlecellVR	StarmapVis	UCSC Cell Browser	vitessce
RNA Velocity	Cone plot	+									
	Volume plot	+									
	Streamlines/streamlets	+									
	Combining multiple types of plots	+									
	Trajectory from selected cell	+									
	Feature changes along the trajectory	+									
General	3D view	+	+		+		+	+	+		+
	Multimodality	+			+			+			+
	Docker	+	+	+		+	+	+			
	Example datasets	+	+	+		+		+	+	+	+
Input files	h5		+				+				
	h5ad	+		+	+		+	+		+	
	h5mu	+									
	csv/tsv/txt	+	+				+		+	+	+
	loom		+			+	+	+		+	
	Seurat/rds		+							+	
Processing	Clustering	+	+								+
	Data normalization	+	+			+					
	Scaling	+	+			+					
	Feature/cell filtering	+	+	+	+	+			+	+	

direction of cellular transitions within the 3D embedding, is highly dependent on the precision of detecting unspliced and spliced RNA forms of key genes and consequently displays heterogeneous transition probability patterns. In contrast, streamlines and streamlets, derived from the integration of individual cell velocity vectors, yield more consistent trajectories toward fully differentiated cells. Additionally, the volume plot effectively depicts the expression level of Pax4, a key transcription factor for endocrine development [66], which is transiently expressed in endocrine progenitors [67]. For the visualization of the BMMC CITE-seq dataset in 3D (2B), three modes are employed simultaneously: a scatter plot to display cell positions, streamlets to indicate the direction of cellular transitions, and a volume plot to portray the epitope levels of the CD34 surface marker, which is specific to hematopoietic stem cells (HSCs). Although epitope levels are readily visualized in conventional scatterplots, RNA expression is substantially sparser. Nonetheless, the volume plot derived from RNA modality data accurately reflects its HSC specificity, highlighting the utility of volume plots in delineating low-abundance features. In a subsequent visualization, the same plot orientation is used to generate a trajectory from a selected cell to erythroid progenitor cells. Segments of trajectory-projected cells are marked to compute differentially expressed feature activity levels, which are then grouped according to their trends and visualized in interactive heatmaps. Users can select the modality for analysis — such as RNA or ADT (epitope) levels — and the heatmaps can display either normalized or relative feature activity levels. Notably, the RNA heatmap highlights a pronounced upregulation of hemoglobin subunit beta (HBB) and hemoglobin alpha 1 (HBA1), components of hemoglobin A, along the erythroid lineage. Conversely, the ADT heatmap reveals a systematic decrease in CD34 protein levels from HSCs, alongside a transient upregulation of CD38 levels along the erythroid trajectory. These observations underscore the platform's robust capacity to elucidate dynamic cellular processes across multiple data modalities.

Discussion

Our objective is to facilitate rapid and in-depth exploration of single-cell developmental data in 3D, thereby providing immediate insights and fostering a nuanced understanding of cellular trajectories. Cell Journey is accessible to researchers regardless of computational expertise, featuring a streamlined setup process that requires only a few simple steps and an intuitive, user-friendly interface. A comprehensive help panel, accompanied by FAQs and sample datasets, enables users to quickly familiarize themselves with the tool's functionalities. Designed with extensibility in mind, Cell Journey accommodates the integration of additional analytical modules, thereby supporting increasingly comprehensive analyses. Moreover, the software is optimized for speed, efficiency, and robustness, ensuring reliable performance even when processing large-scale single-cell datasets. Cell Journey is under active development. We plan to extend its functionality by providing new modules for differential trajectory analysis and integration with workspaces for comprehensive single-cell multimodal data analysis. We actively invite user feedback and feature suggestions to drive the continuous enhancement of its functionality, and we are committed to providing regular updates and fostering active community engagement to ensure the tool's ongoing relevance and innovation.

While Cell Journey was originally developed to visualize transitions between cellular states as inferred from RNA velocity analyses [68], its framework is broadly applicable to any method — whether experimental or computational — that delineates cellular state transitions. For instance, the tool can be employed to visualize ground-truth cellular transitions obtained from lineage-tracing experiments, such as Cas9-based cellular ancestry recording [69] or DNA barcoding [70]. These techniques label cells with unique genetic markers, enabling the reconstruction of clonal dynamics and elucidation of lineage relationships through shared mutation patterns or random DNA sequences integrated into the genome. Furthermore, Cell Journey can be potentially used to integrate and visualize trajectories from transition probabilities between cells computationally inferred using optimal transport algorithms applied to time-course single-cell transcriptomic data [3] or multi-modal or spatial datasets [71]. Finally, we foresee that Cell Journey will be instrumental in visualizing putative cellular transitions within 3D spatial transcriptomics datasets [72], particularly when combined with emerging methodologies for spatial RNA velocity analysis [73–75]. This versatility underscores the potential of Cell Journey to facilitate a comprehensive understanding of cellular dynamics across diverse experimental and computational platforms.

Availability of Source Code and Requirements

- Project name: Cell Journey
- Project homepage: https://github.com/TabakaLab/CellJourney
- Documentation: https://tabakalab.github.io/CellJourney
- Operating system(s): Platform independent
- Programming languages: Python
- Other requirements: Python 3.11.7, Dash 2.14, Plotly 5.18
- License: MIT license

Acknowledgements and funding

We thank members of the Computational Genomics Group for their discussions. D.P. and M.T. are supported by the International Centre for Translational Eye Research (FENG.02.01-IP.05-T005/23) project, which is carried out within the International Research Agendas Program of the Foundation for Polish Science, co-financed by the European Union under the European Regional Development Fund; and grant funded by National Science Center, Poland: the Sonata Bis 12 grant 2022/46/E/NZ2/0037 (M.T.).

Author's Contributions

M.T. conceived and supervised the project. M.T. and D.P. designed and developed Cell Journey. D.P. implemented Cell Journey and wrote its documentation. M.T. and D.P. wrote the manuscript.

Competing Interests

The authors declare no competing interests.

Code availability

Cell Journey is under the MIT License. The software, documentation, tutorials, example datasets, and animated demos can be found at https://TabakaLab.github.io/CellJourney.

References

1. Trapnell C, Cacchiarelli D, Grimsby J, Pokharel P, Li S, Morse M, et al. The dynamics and regulators of cell fate decisions are revealed by pseudotemporal ordering of single cells. Nature Biotechnology 2014;32(4):381–386.
2. Farrell JA, Wang Y, Riesenfeld SJ, Shekhar K, Regev A, Schier AF. Single-cell reconstruction of developmental trajectories during zebrafish embryogenesis. Science 2018;360(6392):eaar3131.
3. Schiebinger G, Shu J, Tabaka M, Cleary B, Subramanian V,

Solomon A, et al. Optimal-Transport Analysis of Single-Cell Gene Expression Identifies Developmental Trajectories in Reprogramming. Cell 2019;176(4):928–943.e22.
4. La Manno G, Siletti K, Furlan A, Gyllborg D, Vinsland E, Mossi Albiach A, et al. Molecular architecture of the developing mouse brain. Nature 2021;596(7870):92–96.
5. La Manno G, Soldatov R, Zeisel A, Braun E, Hochgerner H, Petukhov V, et al. RNA velocity of single cells. Nature 2018;560(7719):494–498.
6. Berker V, Lange M, Paidli S, Wolf FA, Theis FJ. Generalizing RNA velocity to transient cell states through dynamical modeling. Nature Biotechnology 2020;38(12):1408–1414.
7. Gao M, Qiao C, Huang Y. UniTVelo: temporally unified RNA velocity reinforces single-cell trajectory inference. Nature Communications 2022;13(1):6586.
8. Lange M, Bergen V, Klein M, Setty M, Reuter B, Bakhti M, et al. CellRank for directed single-cell fate mapping. Nature Methods 2022;19(2):159–170.
9. Qiu X, Zhang Y, Martin-Rufino JD, Weng C, Hosseinzadeh S, Yang D, et al. Mapping transcriptomic vector fields of single cells. Cell 2022;185(4):690–711.e45.
10. Li C, Virgilio MC, Collins KL, Welch JD. Multi-omic single-cell velocity models epigenome–transcriptome interactions and improves cell fate prediction. Nature Biotechnology 2023;41(3):387–398.
11. Cui H, Maan H, Vladoiu MC, Zhang J, Taylor MD, Wang B. DeepVelo: deep learning extends RNA velocity to multi-lineage systems with cell-specific kinetics. Genome Biology 2024;25(1):27.
12. Gayoso A, Weiler P, Lotfollahi M, Klein D, Hong J, Streets A, et al. Deep generative modeling of transcriptional dynamics for RNA velocity analysis in single cells. Nature Methods 2024;21(1):50–59.
13. Li J, Pan X, Yuan Y, Shen HB. TFvelo: gene regulation inspired RNA velocity estimation. Nature Communications 2024;15(1):1387.
14. Li S, Zhang P, Chen W, Ye L, Brannan KW, Le NT, et al. A relay velocity model infers cell-dependent RNA velocity. Nature Biotechnology 2024;42(1):99–108.
15. Peng Q, Qiu X, Li T. Storm: Incorporating transient stochastic dynamics to infer the RNA velocity with metabolic labeling information. PLOS Computational Biology 2024 11;20(11):1–38.
16. Wang K, Hou L, Wang X, Zhai X, Lu Z, Zi Z, et al. PhyloVelo enhances transcriptomic velocity field mapping using monotonically expressed genes. Nature Biotechnology 2024;42(5):778–789.
17. Ge M, Miao J, Qi J, Zhou X, Lin Z. TIVelo: RNA velocity estimation leveraging cluster-level trajectory inference. Nature Communications 2025;16(1):6258.
18. Aivazidis A, Memi F, Kleshchevnikov V, Er S, Clarke B, Stegle O, et al. Cell2fate infers RNA velocity modules to improve cell fate prediction. Nature Methods 2025;p. 1–10.
19. Wang W, Hu Z, Weiler P, Mayes S, Lange M, Wang J, et al. RegVelo: gene-regulatory-informed dynamics of single cells. bioRxiv 2024;.
20. Chen Y, Zhang Y, Gan J, Ni K, Chen M, Bahar I, et al. GraphVelo allows for accurate inference of multimodal velocities and molecular mechanisms for single cells. bioRxiv 2025;.
21. Li J, Wang Z, Shen HB, Yuan Y. TSvelo: Comprehensive RNA velocity inference by jointly modeling Transcription and Splicing. bioRxiv 2024;.
22. Wolf FA, Hamey FK, Plass M, Solana J, Dahlin JS, Göttgens B, et al. PAGA: graph abstraction reconciles clustering with trajectory inference through a topology preserving map of single cells. Genome Biology 2019;20(1):59.
23. Schwabe D, Formichetti S, Junker JP, Falcke M, Rajewsky N. The transcriptome dynamics of single cells during the cell cycle. Molecular Systems Biology 2020;16(11):e9946.
24. Weng G, Kim J, Won KJ. VeTra: a tool for trajectory inference based on RNA velocity. Bioinformatics 2021;37(20):3509–3513.
25. Zhang Z, Zhang X. Inference of high-resolution trajectories in single-cell RNA-seq data by using RNA velocity. Cell Reports Methods 2021;1(6).
26. Gupta R, Cerletti D, Gut G, Oxenius A, Claassen M. Simulation-based inference of differentiation trajectories from RNA velocity fields. Cell Reports Methods 2022;2(12).
27. Lange M, Bergen V, Klein M, Setty M, Reuter B, Bakhti M, et al. CellRank for directed single-cell fate mapping. Nature Methods 2022;19(2):159–170.
28. Weiler P, Lange M, Klein M, Pe'er D, Theis F. CellRank 2: unified fate mapping in multiview single-cell data. Nature Methods 2024;21(7):1196–1205.
29. Atta L, Sahoo A, Fan J. VeloViz: RNA velocity-informed embeddings for visualizing cellular trajectories. Bioinformatics 2022;38(2):391–396.
30. Xia L, Lee C, Li JJ. Statistical method scDEED for detecting dubious 2D single-cell embeddings and optimizing t-SNE and UMAP hyperparameters. Nature Communications 2024;15(1):1753.
31. Sun ED, Ma R, Zou J. Dynamic visualization of high-dimensional data. Nature Computational Science 2023;3(1):86–100.
32. Rutkowski P, Tabaka M. Ocelli: an open-source tool for the analysis and visualization of developmental multimodal single-cell data. NAR Genomics and Bioinformatics 2025;7(2):lqaf040.
33. Cao J, Cusanovich DA, Ramani V, Aghamirzaie D, Pliner HA, Hill AJ, et al. Joint profiling of chromatin accessibility and gene expression in thousands of single cells. Science 2018;361(6409):1380–1385.
34. Zhu C, Yu M, Huang H, Juric I, Abnousi A, Hu R, et al. An ultra high-throughput method for single-cell joint analysis of open chromatin and transcriptome. Nature Structural & Molecular Biology 2019;26(11):1063–1070.
35. Ma S, Zhang B, LaFave LM, Earl AS, Chiang Z, Hu Y, et al. Chromatin potential identified by shared single-cell profiling of RNA and chromatin. Cell 2020;183(4):1103–1116.
36. Chen S, Lake BB, Zhang K. High-throughput sequencing of the transcriptome and chromatin accessibility in the same cell. Nature Biotechnology 2019;37(12):1452–1457.
37. Hunt KV, Burnard SM, Roper EA, Bond DR, Dun MD, Verrills NM, et al. scTEM-seq: Single-cell analysis of transposable element methylation to link global epigenetic heterogeneity with transcriptional programs. Scientific Reports 2022;12(1):5776.
38. Zhu C, Zhang Y, Li YE, Lucero J, Behrens MM, Ren B. Joint profiling of histone modifications and transcriptome in single cells from mouse brain. Nature Methods 2021;18(3):283–292.
39. Pan L, Ku WL, Tang Q, Cao Y, Zhao K. scPCOR-seq enables co-profiling of chromatin occupancy and RNAs in single cells. Communications Biology 2022;5(1):678.
40. Tedesco M, Giannese F, Lazarević D, Giansanti V, Rosano D, Monzani S, et al. Chromatin Velocity reveals epigenetic dynamics by single-cell profiling of heterochromatin and euchromatin. Nature Biotechnology 2022;40(2):235–244.
41. Bartosovic M, Kabbe M, Castelo-Branco G. Single-cell CUT&Tag profiles histone modifications and transcription factors in complex tissues. Nature Biotechnology 2021;39(7):825–835.
42. Gopalan S, Wang Y, Harper NW, Garber M, Fazzio TG. Simultaneous profiling of multiple chromatin proteins in the same cells. Molecular Cell 2021;81(22):4736–4746.
43. Stuart T, Hao S, Zhang B, Mekerishvili L, Landau DA, Maniatis S, et al. Nanobody-tethered transposition enables multifactorial chromatin profiling at single-cell resolution. Nature Biotechnology 2022;p. 1–7.
44. Bartosovic M, Castelo-Branco G. Multimodal chromatin profiling using nanobody-based single-cell CUT&Tag. Nature Biotechnology 2022;p. 1–12.

45. Yeung J, Florescu M, Zeller P, de Barbanson BA, Wellenstein MD, van Oudenaarden A. scChIX-seq infers dynamic relationships between histone modifications in single cells. Nature Biotechnology 2023;p. 1–11.
46. Stoeckius M, Hafemeister C, Stephenson W, Houck-Loomis B, Chattopadhyay PK, Swerdlow H, et al. Simultaneous epitope and transcriptome measurement in single cells. Nature Methods 2017;14(9):865–868.
47. Mimitou EP, Lareau CA, Chen KY, Zorzetto-Fernandes AL, Hao Y, Takeshima Y, et al. Scalable, multimodal profiling of chromatin accessibility, gene expression and protein levels in single cells. Nature Biotechnology 2021;39(10):1246–1258.
48. Wang S, Sontag ED, Lauffenburger DA. What cannot be seen correctly in 2D visualizations of single-cell 'omics data? Cell Systems 2023;14(9):723–731.
49. Bastidas-Ponce A, Tritschler S, Dony L, Scheibner K, Tarquis-Medina M, Salinno C, et al. Comprehensive single cell mRNA profiling reveals a detailed roadmap for pancreatic endocrinogenesis. Development 2019 06;146(12):dev173849.
50. Stuart T, Butler A, Hoffman P, Hafemeister C, Papalexi E, Mauck WM, et al. Comprehensive integration of single-cell data. Cell 2019;177(7):1888–1902.
51. Wolf FA, Angerer P, Theis FJ. SCANPY: large-scale single-cell gene expression data analysis. Genome Biology 2018;19(1):15.
52. Hao Y, Hao S, Andersen-Nissen E, Mauck WMr, Zheng S, Butler A, et al. Integrated analysis of multimodal single-cell data. Cell 2021;184(13):3573–3587.e29.
53. Bredikhin D, Kats I, Stegle O. MUON: multimodal omics analysis framework. Genome Biology 2022;23(1):42.
54. McInnes L, Healy J, Melville J. Umap: Uniform manifold approximation and projection for dimension reduction. arXiv preprint arXiv:180203426 2018;.
55. Jacomy M, Venturini T, Heymann S, Bastian M. ForceAtlas2, a continuous graph layout algorithm for handy network visualization designed for the Gephi software. PLOS ONE 2014;9(6):e98679.
56. Ding J, Regev A. Deep generative model embedding of single-cell RNA-Seq profiles on hyperspheres and hyperbolic spaces. Nature Communications 2021;12(1):2554.
57. Gardeux V, David FPA, Shajkofci A, Schwalie PC, Deplancke B. ASAP: a web-based platform for the analysis and interactive visualization of single-cell RNA-seq data. Bioinformatics 2017;33(19):3123–3125.
58. Program CCS, Abdulla S, Aevermann B, Assis P, Badajoz S, Bell SM, et al. CZ CELLxGENE Discover: a single-cell data platform for scalable exploration, analysis and modeling of aggregated data. Nucleic Acids Research 2024 11;53(D1):D886–D900.
59. Hyman L, Sbalzarini IF, Quake S, Günther U, Corvo: Visualizing CellxGene Single-Cell Datasets in Virtual Reality; 2022.
60. Davie K, Janssens J, Koldere D, De Waegeneer M, Pech U, Kreft L, et al. A Single-Cell Transcriptome Atlas of the Aging Drosophila Brain. Cell 2018;174(4):982–998.e20.
61. Tabaka M, Gould J, Regev A. scSVA: an interactive tool for big data visualization and exploration in single-cell omics. bioRxiv 2019;.
62. Stein DF, Chen H, Vinyard ME, Qin Q, Combs RD, Zhang Q, et al. singlecellVR: Interactive Visualization of Single-Cell Data in Virtual Reality. Frontiers in Genetics 2021;12.
63. Ma S, Fang X, Yao Y, Li J, Morgan DC, Xia Y, et al. StarmapVis: An interactive and narrative visualisation tool for single-cell and spatial data. Computational and Structural Biotechnology Journal 2023;21:1598–1605.
64. Speir ML, Bhaduri A, Markov NS, Moreno P, Nowakowski TJ, Papatheodorou I, et al. UCSC Cell Browser: visualize your single-cell data. Bioinformatics 2021 07;37(23):4578–4580.
65. Keller MS, Gold I, McCallum C, Manz T, Kharchenko PV, Gehlenborg N. Vitessce: integrative visualization of multimodal and spatially resolved single-cell data. Nature Methods 2025;22(1):63–67.
66. Collombat P, Mansouri A, Hecksher-Sørensen J, Serup P, Krull J, Gradwohl G, et al. Opposing actions of Arx and Pax4 in endocrine pancreas development. Genes & Development 2003;17(20):2591–2603.
67. Yu XX, Qiu WL, Yang L, Wang YC, He MY, Wang D, et al. Sequential progenitor states mark the generation of pancreatic endocrine lineages in mice and humans. Cell Research 2021;31(8):886–903.
68. Wang Y, Li J, Zha H, Liu S, Huang D, Fu L, et al. Paradigms, innovations, and biological applications of RNA velocity: a comprehensive review. Briefings in Bioinformatics 2025;26(4):bbaf339.
69. McKenna A, Findlay GM, Gagnon JA, Horwitz MS, Schier AF, Shendure J. Whole-organism lineage tracing by combinatorial and cumulative genome editing. Science 2016;353(6298):aaf7907.
70. Gutierrez C, Al'Khafaji A, Brenner E, Johnson K, Gohil S, Lin Z, et al. Multifunctional barcoding with ClonMapper enables high-resolution study of clonal dynamics during tumor evolution and treatment. DOI: https://doi org/101038/s43018-021-00222-8 2021;2(7):758–772.
71. Klein D, Palla G, Lange M, Klein M, Piran Z, Gander M, et al. Mapping cells through time and space with moscot. Nature 2025;638(8052):1065–1075.
72. Schott M, León-Periñán D, Splendiani E, Strenger L, Licha JR, Pentimalli TM, et al. Open-ST: High-resolution spatial transcriptomics in 3D. Cell 2024;187(15):3953–3972.
73. Abdelaal T, Grossouw LM, Pasterkamp RJ, Lelieveldt BP, Reinders MJ, Mahfouz A. SIRV: spatial inference of RNA velocity at the single-cell resolution. NAR Genomics and Bioinformatics 2024;6(3):lqae100.
74. Zhou P, Bocci F, Li T, Nie Q. Spatial transition tensor of single cells. Nature Methods 2024;21(6):1053–1062.
75. Gu Y, Liu J, Lee KH, Li C, Lu L, Moline J, et al. Topological velocity inference from spatial transcriptomic data. Nature Biotechnology 2025;p. 1–12.

Cover Letter Click here to access/download;Personal Cover;CoverLetter.pdf

The Editor

GigaScience

August 12th, 2025

Dear Editor,

Please find enclosed our manuscript entitled "**Interactive analysis of single-cell trajectories in 3D space with Cell Journey**", which we submit for publication as a Technical Note to GigaScience.

Single-cell genomics has grown dramatically in the last decade, enabling simultaneous genome-wide measurements of multiple modalities in the same single cell. This revolution allows researchers to reconstruct cellular differentiation trajectories at unprecedented resolution. The state-of-the-art computational tools used to infer the transitions between the cellular states include methods based on RNA velocity. The cellular transitions are commonly depicted on a two-dimensional (2D) embedding either by arrows or streamlines, which, in the case of intricate developmental single-cell data, is insufficient to represent complex topologies of multipartite cellular transitions.

Here, we present Cell Journey, a lightweight Python package with an interactive Dash interface that addresses this limitation by enabling multimodal visualization and analysis of RNA velocity-based single-cell trajectories.

The main features of the Cell Journey package are:

- Interactive visualization of single-cell RNA velocity-based trajectories in **three-dimensional** (3D) space.
- Computation of **RNA velocity 3D field lines** on user-specified grid resolution and their multimode visualization as streamlines or streamlets.
- Interactive computation of RNA velocity-based trajectory from user-selected cell or region on the grid.
- Computation of **multimodal features trends** and their clustering along the selected or generated trajectory.
- Novel visualization of continuous feature activities as **3D volume plots** that mitigates the inherent dropout present in single-cell profiling methods. This function enhances the visualization of low-activity features.
- Exploration of single-cell multimodal data embeddings with 3D scatterplots such as visualization of (i) clusters or other categorical cell metadata, (ii) continuous feature activities, e.g. gene expression, protein epitope levels, and chromatin accessibility.
- Comprehensive customization of 3D scatterplots allowing users to precisely define a scale or color palette or select one of the many built-in sets with colorblind-friendly options.
- Support for single- and multimodal datasets in **h5ad, h5mu, and csv** format.
- Exports of generated figures either as static images in raster and vector format (png, tiff, jpeg, pdf, svg) or interactive to be explored in a web browser.

Cell Journey package is timely and of interest to the broad readership of ***GigaScience***. It is available with installation instructions and extensive documentation at https://TabakaLab.github.io/CellJourney

Sincerely,

Marcin Tabaka
