# Supplementary material for: Interactive analysis of single-cell trajectories in 3D space with Cell Journey
Source: Gigascience. 2026 Mar 3;15:giag021. doi: 10.1093/gigascience/giag021 (PMC13042281; doi:10.1093/gigascience/giag021)
Supplement: giag021_GIGA-D-25-00322_Revision_1 [file giag021_giga-d-25-00322_revision_1.pdf]

GigaScience

Interactive analysis of single-cell trajectories in 3D space with Cell Journey

--Manuscript Draft--

**Manuscript Number:**	GIGA-D-25-00322R1
**Full Title:**	Interactive analysis of single-cell trajectories in 3D space with Cell Journey
**Article Type:**	Technical Note
**Funding Information:**	Fundacja na rzecz Nauki Polskiej (FENG.02.01-IP.05-T005/23) — Dr Marcin Tabaka
**Abstract:**	The integration of high-throughput single-cell profiling technologies with RNA velocity analysis has enabled the reconstruction<br>of dynamic cellular differentiation trajectories at unprecedented resolution. Despite these advances, current visualization<br>techniques for RNA velocity are predominantly confined to two-dimensional representations, typically employing arrows or<br>streamlines. While effective for depicting simple cellular trajectories, these approaches are insufficient for capturing the complex<br>topologies of multipartite cellular transitions. This limitation highlights the need for advanced three-dimensional visualization<br>tools that can more accurately convey the structure and dynamics of velocity-inferred transitions in single-cell data. Here, we<br>present Cell Journey, an interactive visualization platform specifically developed for three-dimensional analysis and<br>representation of RNA velocity trajectories derived from single-cell datasets. The platform features an intuitive graphical interface<br>supporting both unimodal and multimodal data, accommodates multiple input formats, and provides extensive customization<br>capabilities for trajectory visualization. Cell Journey computes RNA velocity vector fields on a user-defined three-dimensional grid<br>and constructs velocity trajectories using either Euler integration or the fourth-order Runge-Kutta method. The platform enables<br>dynamic exploration of cellular dynamics through interactive visual elements, including streamlines, streamlets, cones, and<br>volumetric plots. Furthermore, it allows users to investigate changes in feature activity along selected paths, facilitating deeper<br>insights into cellular state transitions within complex multimodal single-cell datasets.
**Corresponding Author:**	Marcin Tabaka<br>Institute of Physical Chemistry PAS: Polska Akademia Nauk Instytut Chemii Fizycznej<br>Warsaw, POLAND
**Corresponding Author Secondary Information:**	
**Corresponding Author's Institution:**	Institute of Physical Chemistry PAS: Polska Akademia Nauk Instytut Chemii Fizycznej
**Corresponding Author's Secondary Institution:**	
**First Author:**	Marcin Tabaka
**First Author Secondary Information:**	
**Order of Authors:**	Marcin Tabaka
	Damian Panas
**Order of Authors Secondary Information:**	
**Response to Reviewers:**	Dear Editor,<br><br>Thank you for the constructive feedback provided by you and the Reviewers regarding our manuscript, “Interactive analysis of single-cell trajectories in 3D space with Cell Journey” (GIGA-D-25-00322). We appreciate the opportunity to revise the manuscript

Powered by Editorial Manager® and ProduXion Manager® from Aries Systems Corporation

	for potential publication in GigaScience.<br><br>We have carefully considered all Reviewer comments and have revised the manuscript extensively to address the essential points raised. A detailed, point-by-point response to each Reviewer comment is provided in the "Cell Journey responses to the reviewers.pdf" indicating how each point was addressed in the manuscript.<br>In addition, we have updated the public code and/or data resources as needed to reflect the revisions described in the manuscript.<br><br>Thank you again for your time and consideration. We hope the revised manuscript and accompanying responses satisfactorily address all concerns, and we look forward to your further evaluation.<br><br>Sincerely,<br>Marcin Tabaka, PhD<br>Damian Panas, PhD
**Additional Information:**	
**Question**	**Response**
Are you submitting this manuscript to a special series or article collection?	No
**Experimental design and statistics**<br><br>Full details of the experimental design and statistical methods used should be given in the Methods section, as detailed in our Minimum Standards Reporting Checklist. Information essential to interpreting the data presented should be made available in the figure legends.<br><br>Have you included all the information requested in your manuscript?	Yes
**Resources**<br><br>A description of all resources used, including antibodies, cell lines, animals and software tools, with enough information to allow them to be uniquely identified, should be included in the Methods section. Authors are strongly encouraged to cite Research Resource Identifiers (RRIDs) for antibodies, model organisms and tools, where possible.<br><br>Have you included the information requested as detailed in our Minimum Standards Reporting Checklist?	Yes

**Availability of data and materials**<br><br>All datasets and code on which the conclusions of the paper rely must be either included in your submission or deposited in publicly available repositories (where available and ethically appropriate), referencing such data using a unique identifier in the references and in the “Availability of Data and Materials” section of your manuscript.<br><br>Have you have met the above requirement as detailed in our Minimum Standards Reporting Checklist?	Yes
GigaScience has policies and guidelines in place for the use of generative AI-writing tools such as ChatGPT. If you have used such writing tools to assist with writing the manuscript this must be declared and cited in the text. Authors should not list AI-writing tools and other AI-assisted technologies as an author or co-author and should acknowledge that they are fully responsible for text generated or refined by AI-writing tools.<p><br><br>A summary of use (particularly in the introduction or among methods) needs to be included at the end of the paper, and the outputs should also be included as a supplementary file hosted in GigaDB or other open repositories. Please read our guidelines for more information. <p><br><br>By submitting to GigaScience, you are aware of the journal's AI-writing tools policy, and if you have declared use of such tools below, you have acknowledged this where appropriate in your manuscript and have made a summary of use and outputs available. <p><br><b>AI-assisted writing tools have been used in the preparation of this manuscript?	No

Main text Click here to access/download;LaTeX - Main Document (TeX file);main.tex

```
This is pdfTeX, Version 3.141592653-2.6-1.40.26 (TeX Live 2024)
(preloaded format=pdflatex 2024.8.2)  26 FEB 2026 22:14
entering extended mode
 restricted \write18 enabled.
 %&-line parsing enabled.
**main.tex
(./main.tex
LaTeX2e <2024-06-01> patch level 2
L3 programming layer <2024-05-27>
(./oup-contemporary.cls
Document Class: oup-contemporary 2023/06/12, v1.2
(c:/texlive/2024/texmf-dist/tex/latex/base/article.cls
Document Class: article 2024/02/08 v1.4n Standard LaTeX document class
(c:/texlive/2024/texmf-dist/tex/latex/base/size10.clo
File: size10.clo 2024/02/08 v1.4n Standard LaTeX file (size option)
)
\c@part=\count194
\c@section=\count195
\c@subsection=\count196
\c@subsubsection=\count197
\c@paragraph=\count198
\c@subparagraph=\count199
\c@figure=\count266
\c@table=\count267
\abovecaptionskip=\skip49
\belowcaptionskip=\skip50
\bibindent=\dimen141
) (c:/texlive/2024/texmf-dist/tex/latex/base/inputenc.sty
Package: inputenc 2024/02/08 v1.3d Input encoding file
\inpenc@prehook=\toks17
\inpenc@posthook=\toks18
) (c:/texlive/2024/texmf-dist/tex/latex/base/fontenc.sty
Package: fontenc 2021/04/29 v2.0v Standard LaTeX package
) (c:/texlive/2024/texmf-dist/tex/generic/iftex/ifpdf.sty
Package: ifpdf 2019/10/25 v3.4 ifpdf legacy package. Use iftex instead.
(c:/texlive/2024/texmf-dist/tex/generic/iftex/iftex.sty
Package: iftex 2022/02/03 v1.0f TeX engine tests
)) (c:/texlive/2024/texmf-dist/tex/latex/microtype/microtype.sty
Package: microtype 2024/03/29 v3.1b Micro-typographical refinements (RS)
(c:/texlive/2024/texmf-dist/tex/latex/graphics/keyval.sty
Package: keyval 2022/05/29 v1.15 key=value parser (DPC)
\KV@toks@=\toks19
) (c:/texlive/2024/texmf-dist/tex/latex/etoolbox/etoolbox.sty
Package: etoolbox 2020/10/05 v2.5k e-TeX tools for LaTeX (JAW)
\etb@tempcnta=\count268
)
\MT@toks=\toks20
\MT@tempbox=\box52
\MT@count=\count269
LaTeX Info: Redefining \noprotrusionifhmode on input line 1061.
LaTeX Info: Redefining \leftprotrusion on input line 1062.
\MT@prot@toks=\toks21
LaTeX Info: Redefining \rightprotrusion on input line 1081.
LaTeX Info: Redefining \textls on input line 1392.
```

```
\MT@outer@kern=\dimen142
LaTeX Info: Redefining \textmicrotypecontext on input line 2013.
\MT@listname@count=\count270
(c:/texlive/2024/texmf-dist/tex/latex/microtype/microtype-pdftex.def
File: microtype-pdftex.def 2024/03/29 v3.1b Definitions specific to
pdftex (RS)

LaTeX Info: Redefining \lsstyle on input line 902.
LaTeX Info: Redefining \lslig on input line 902.
\MT@outer@space=\skip51
)
Package microtype Info: Loading configuration file microtype.cfg.
(c:/texlive/2024/texmf-dist/tex/latex/microtype/microtype.cfg
File: microtype.cfg 2024/03/29 v3.1b microtype main configuration file
(RS)
)) (c:/texlive/2024/texmf-dist/tex/latex/euler/euler.sty
Package: euler 1995/03/05 v2.5
Package: `euler' v2.5 <1995/03/05> (FJ and FMi)
LaTeX Font Info:    Redeclaring symbol font `letters' on input line 35.
LaTeX Font Info:    Encoding `OML' has changed to `U' for symbol font
(Font)              `letters' in the math version `normal' on input line
35.
LaTeX Font Info:    Overwriting symbol font `letters' in version `normal'
(Font)                  OML/cmm/m/it --> U/eur/m/n on input line 35.
LaTeX Font Info:    Encoding `OML' has changed to `U' for symbol font
(Font)              `letters' in the math version `bold' on input line
35.
LaTeX Font Info:    Overwriting symbol font `letters' in version `bold'
(Font)                  OML/cmm/b/it --> U/eur/m/n on input line 35.
LaTeX Font Info:    Overwriting symbol font `letters' in version `bold'
(Font)                  U/eur/m/n --> U/eur/b/n on input line 36.
LaTeX Font Info:    Redeclaring math symbol \Gamma on input line 47.
LaTeX Font Info:    Redeclaring math symbol \Delta on input line 48.
LaTeX Font Info:    Redeclaring math symbol \Theta on input line 49.
LaTeX Font Info:    Redeclaring math symbol \Lambda on input line 50.
LaTeX Font Info:    Redeclaring math symbol \Xi on input line 51.
LaTeX Font Info:    Redeclaring math symbol \Pi on input line 52.
LaTeX Font Info:    Redeclaring math symbol \Sigma on input line 53.
LaTeX Font Info:    Redeclaring math symbol \Upsilon on input line 54.
LaTeX Font Info:    Redeclaring math symbol \Phi on input line 55.
LaTeX Font Info:    Redeclaring math symbol \Psi on input line 56.
LaTeX Font Info:    Redeclaring math symbol \Omega on input line 57.
\symEulerFraktur=\mathgroup4
LaTeX Font Info:    Overwriting symbol font `EulerFraktur' in version
`bold'
(Font)                  U/euf/m/n --> U/euf/b/n on input line 63.
LaTeX Info: Redefining \oldstylenums on input line 85.
\symEulerScript=\mathgroup5
LaTeX Font Info:    Overwriting symbol font `EulerScript' in version
`bold'
(Font)                  U/eus/m/n --> U/eus/b/n on input line 93.
LaTeX Font Info:    Redeclaring math symbol \aleph on input line 97.
LaTeX Font Info:    Redeclaring math symbol \Re on input line 98.
LaTeX Font Info:    Redeclaring math symbol \Im on input line 99.
```

```
LaTeX Font Info:    Redeclaring math delimiter \vert on input line 101.
LaTeX Font Info:    Redeclaring math delimiter \backslash on input line
103.
LaTeX Font Info:    Redeclaring math symbol \neg on input line 106.
LaTeX Font Info:    Redeclaring math symbol \wedge on input line 108.
LaTeX Font Info:    Redeclaring math symbol \vee on input line 110.
LaTeX Font Info:    Redeclaring math symbol \setminus on input line 112.
LaTeX Font Info:    Redeclaring math symbol \sim on input line 113.
LaTeX Font Info:    Redeclaring math symbol \mid on input line 114.
LaTeX Font Info:    Redeclaring math delimiter \arrowvert on input line
116.
LaTeX Font Info:    Redeclaring math symbol \mathsection on input line
117.
\symEulerExtension=\mathgroup6
LaTeX Font Info:    Redeclaring math symbol \coprod on input line 125.
LaTeX Font Info:    Redeclaring math symbol \prod on input line 125.
LaTeX Font Info:    Redeclaring math symbol \sum on input line 125.
LaTeX Font Info:    Redeclaring math symbol \intop on input line 130.
LaTeX Font Info:    Redeclaring math symbol \ointop on input line 131.
LaTeX Font Info:    Redeclaring math symbol \braceld on input line 132.
LaTeX Font Info:    Redeclaring math symbol \bracerd on input line 133.
LaTeX Font Info:    Redeclaring math symbol \bracelu on input line 134.
LaTeX Font Info:    Redeclaring math symbol \braceru on input line 135.
LaTeX Font Info:    Redeclaring math symbol \infty on input line 136.
LaTeX Font Info:    Redeclaring math symbol \nearrow on input line 153.
LaTeX Font Info:    Redeclaring math symbol \searrow on input line 154.
LaTeX Font Info:    Redeclaring math symbol \nwarrow on input line 155.
LaTeX Font Info:    Redeclaring math symbol \swarrow on input line 156.
LaTeX Font Info:    Redeclaring math symbol \Leftrightarrow on input line
157.
LaTeX Font Info:    Redeclaring math symbol \Leftarrow on input line 158.
LaTeX Font Info:    Redeclaring math symbol \Rightarrow on input line
159.
LaTeX Font Info:    Redeclaring math symbol \leftrightarrow on input line
160.
LaTeX Font Info:    Redeclaring math symbol \leftarrow on input line 161.
LaTeX Font Info:    Redeclaring math symbol \rightarrow on input line
163.
LaTeX Font Info:    Redeclaring math delimiter \uparrow on input line
166.
LaTeX Font Info:    Redeclaring math delimiter \downarrow on input line
168.
LaTeX Font Info:    Redeclaring math delimiter \updownarrow on input line
170.
LaTeX Font Info:    Redeclaring math delimiter \Uparrow on input line
172.
LaTeX Font Info:    Redeclaring math delimiter \Downarrow on input line
174.
LaTeX Font Info:    Redeclaring math delimiter \Updownarrow on input line
176.
LaTeX Font Info:    Redeclaring math symbol \leftharpoonup on input line
177.
LaTeX Font Info:    Redeclaring math symbol \leftharpoondown on input
line 178.
```

```
LaTeX Font Info:    Redeclaring math symbol \rightharpoonup on input line
179.
LaTeX Font Info:    Redeclaring math symbol \rightharpoondown on input
line 180
.
LaTeX Font Info:    Redeclaring math delimiter \lbrace on input line 182.
LaTeX Font Info:    Redeclaring math delimiter \rbrace on input line 184.
\symcmmigroup=\mathgroup7
LaTeX Font Info:    Overwriting symbol font `cmmigroup' in version `bold'
(Font)                  OML/cmm/m/it --> OML/cmm/b/it on input line 200.
LaTeX Font Info:    Redeclaring math accent \vec on input line 201.
LaTeX Font Info:    Redeclaring math symbol \triangleleft on input line
202.
LaTeX Font Info:    Redeclaring math symbol \triangleright on input line
203.
LaTeX Font Info:    Redeclaring math symbol \star on input line 204.
LaTeX Font Info:    Redeclaring math symbol \lhook on input line 205.
LaTeX Font Info:    Redeclaring math symbol \rhook on input line 206.
LaTeX Font Info:    Redeclaring math symbol \flat on input line 207.
LaTeX Font Info:    Redeclaring math symbol \natural on input line 208.
LaTeX Font Info:    Redeclaring math symbol \sharp on input line 209.
LaTeX Font Info:    Redeclaring math symbol \smile on input line 210.
LaTeX Font Info:    Redeclaring math symbol \frown on input line 211.
LaTeX Font Info:    Redeclaring math accent \grave on input line 245.
LaTeX Font Info:    Redeclaring math accent \acute on input line 246.
LaTeX Font Info:    Redeclaring math accent \tilde on input line 247.
LaTeX Font Info:    Redeclaring math accent \ddot on input line 248.
LaTeX Font Info:    Redeclaring math accent \check on input line 249.
LaTeX Font Info:    Redeclaring math accent \breve on input line 250.
LaTeX Font Info:    Redeclaring math accent \bar on input line 251.
LaTeX Font Info:    Redeclaring math accent \dot on input line 252.
LaTeX Font Info:    Redeclaring math accent \hat on input line 254.
) (c:/texlive/2024/texmf-dist/tex/latex/merriweather/merriweather.sty
Package: merriweather 2022/09/20 (Bob Tennent) Supports
Merriweather(Sans) font
s for all LaTeX engines.
(c:/texlive/2024/texmf-dist/tex/generic/iftex/ifxetex.sty
Package: ifxetex 2019/10/25 v0.7 ifxetex legacy package. Use iftex
instead.
) (c:/texlive/2024/texmf-dist/tex/generic/iftex/ifluatex.sty
Package: ifluatex 2019/10/25 v1.5 ifluatex legacy package. Use iftex
instead.
) (c:/texlive/2024/texmf-dist/tex/latex/base/textcomp.sty
Package: textcomp 2024/04/24 v2.1b Standard LaTeX package
) (c:/texlive/2024/texmf-dist/tex/latex/xkeyval/xkeyval.sty
Package: xkeyval 2022/06/16 v2.9 package option processing (HA)
(c:/texlive/2024/texmf-dist/tex/generic/xkeyval/xkeyval.tex
(c:/texlive/2024/te
xmf-dist/tex/generic/xkeyval/xkvutils.tex
\XKV@toks=\toks22
\XKV@tempa@toks=\toks23
)
\XKV@depth=\count271
```

```
File: xkeyval.tex 2014/12/03 v2.7a key=value parser (HA)
)) (c:/texlive/2024/texmf-dist/tex/latex/base/fontenc.sty
Package: fontenc 2021/04/29 v2.0v Standard LaTeX package
) (c:/texlive/2024/texmf-dist/tex/latex/fontaxes/fontaxes.sty
Package: fontaxes 2020/07/21 v1.0e Font selection axes
LaTeX Info: Redefining \upshape on input line 29.
LaTeX Info: Redefining \itshape on input line 31.
LaTeX Info: Redefining \slshape on input line 33.
LaTeX Info: Redefining \swshape on input line 35.
LaTeX Info: Redefining \scshape on input line 37.
LaTeX Info: Redefining \sscshape on input line 39.
LaTeX Info: Redefining \ulcshape on input line 41.
LaTeX Info: Redefining \textsw on input line 47.
LaTeX Info: Redefining \textssc on input line 48.
LaTeX Info: Redefining \textulc on input line 49.
)) (c:/texlive/2024/texmf-dist/tex/latex/mathastext/mathastext.sty
Package: mathastext 2024/07/27 v1.4b Use the text font in math mode (JFB)

Package mathastext Info: Starting the math mode configuration.
\mst@exists@muskip=\muskip17
\mst@forall@muskip=\muskip18
\mst@prime@muskip=\muskip19
\mst@do@nonletters=\toks24
\mst@undo@nonletters=\toks25
\mst@do@easynonletters=\toks26
\mst@undo@easynonletters=\toks27
\symmtoperatorfont=\mathgroup8
\symmtletterfont=\mathgroup9
(   mathastext:   ) ! and ?
(   mathastext:   ) punctuation: , . : ; and \colon
LaTeX Info: Redefining \relbar on input line 1201.
LaTeX Info: Redefining \rightarrowfill on input line 1202.
LaTeX Info: Redefining \leftarrowfill on input line 1205.
(   mathastext:   ) + and =
LaTeX Info: Redefining \Relbar on input line 1298.
(   mathastext:   ) adding = ; and + to \nfss@catcodes
(   mathastext:   ) parentheses ( ) [ ] and slash /
(   mathastext:   ) alldelims: < > \backslash \setminus | \vert \mid \{
\}
LaTeX Font Info:    Redeclaring math symbol \setminus on input line 1364.
LaTeX Info: Redefining \models on input line 1383.
(   mathastext:   ) \# \mathdollar \% \&
(   mathastext:   ) \imath and \jmath
LaTeX Font Info:    Overwriting math alphabet `\Mathnormalbold' in
version `nor
mal'
(Font)                  T1/Merriwthr-OsF/b/it --> T1/Merriwthr-OsF/b/it
on inpu
t line 2863.
LaTeX Font Info:    Overwriting math alphabet `\Mathnormalbold' in
version `bol
d'
(Font)                  T1/Merriwthr-OsF/b/it --> T1/Merriwthr-OsF/b/it
on inpu
```

```
t line 2863.
LaTeX Font Info:    Overwriting symbol font `mtletterfont' in version
`normal'
(Font)                  T1/Merriwthr-OsF/m/it --> T1/Merriwthr-OsF/m/it
on inpu
t line 2863.
LaTeX Font Info:    Overwriting symbol font `mtletterfont' in version
`bold'
(Font)                  T1/Merriwthr-OsF/m/it --> T1/Merriwthr-OsF/b/it
on inpu
t line 2863.
LaTeX Font Info:    Overwriting symbol font `mtoperatorfont' in version
`normal
'
(Font)                  T1/Merriwthr-OsF/m/n --> T1/Merriwthr-OsF/m/n on
input
line 2863.
LaTeX Font Info:    Overwriting symbol font `mtoperatorfont' in version
`bold'
(Font)                  T1/Merriwthr-OsF/m/n --> T1/Merriwthr-OsF/b/n on
input
line 2863.
LaTeX Font Info:    Overwriting math alphabet `\Mathbf' in version
`normal'
(Font)                  T1/Merriwthr-OsF/b/n --> T1/Merriwthr-OsF/b/n on
input
line 2863.
LaTeX Font Info:    Overwriting math alphabet `\Mathbf' in version `bold'
(Font)                  T1/Merriwthr-OsF/b/n --> T1/Merriwthr-OsF/b/n on
input
line 2863.
LaTeX Font Info:    Overwriting math alphabet `\Mathit' in version
`normal'
(Font)                  T1/Merriwthr-OsF/m/it --> T1/Merriwthr-OsF/m/it
on inpu
t line 2863.
LaTeX Font Info:    Overwriting math alphabet `\Mathit' in version `bold'
(Font)                  T1/Merriwthr-OsF/m/it --> T1/Merriwthr-OsF/b/it
on inpu
t line 2863.
LaTeX Font Info:    Overwriting math alphabet `\Mathsf' in version
`normal'
(Font)                  T1/MerriwthrSans-OsF/m/n --> T1/MerriwthrSans-
OsF/m/n o
n input line 2863.
LaTeX Font Info:    Overwriting math alphabet `\Mathsf' in version `bold'
(Font)                  T1/MerriwthrSans-OsF/m/n --> T1/MerriwthrSans-
OsF/b/n o
n input line 2863.
LaTeX Font Info:    Overwriting math alphabet `\Mathtt' in version
`normal'
(Font)                  T1/lmtt/m/n --> T1/lmtt/m/n on input line 2863.
LaTeX Font Info:    Overwriting math alphabet `\Mathtt' in version `bold'
(Font)                  T1/lmtt/m/n --> T1/lmtt/b/n on input line 2863.
```

```
(   mathastext:   ) Latin letters in the `normal', resp. `bold',
(   mathastext:   ) math versions are now set up to use the fonts
(   mathastext:   ) T1/Merriwthr-OsF/m/it, resp. T1/Merriwthr-OsF/b/it.
(   mathastext:   ) Other characters (digits, ...) and \log-like names
will be
(   mathastext:   ) typeset with the n shape.
(   mathastext:   ) \hbar
(   mathastext:   ) minus as endash
(   mathastext:   ) The italic option is in effect.
(   mathastext:   ) \HUGE has been (re)-defined.
(   mathastext:   ) mathastext has declared larger sizes for subscripts.
(   mathastext:   ) To keep LaTeX defaults, use option
`defaultmathsizes'.

Package mathastext Info: Loading is complete.  You can now use
\Mathastext to
(mathastext)             modify the normal and bold math versions.  Use
it
(mathastext)             with optional argument or use \MTDeclareVersion
to
(mathastext)             declare additional math versions.
) (c:/texlive/2024/texmf-dist/tex/latex/relsize/relsize.sty
Package: relsize 2013/03/29 ver 4.1
) (c:/texlive/2024/texmf-dist/tex/latex/ragged2e/ragged2e.sty
Package: ragged2e 2023/06/22 v3.6 ragged2e Package
\CenteringLeftskip=\skip52
\RaggedLeftLeftskip=\skip53
\RaggedRightLeftskip=\skip54
\CenteringRightskip=\skip55
\RaggedLeftRightskip=\skip56
\RaggedRightRightskip=\skip57
\CenteringParfillskip=\skip58
\RaggedLeftParfillskip=\skip59
\RaggedRightParfillskip=\skip60
\JustifyingParfillskip=\skip61
\CenteringParindent=\skip62
\RaggedLeftParindent=\skip63
\RaggedRightParindent=\skip64
\JustifyingParindent=\skip65
) (c:/texlive/2024/texmf-dist/tex/latex/xcolor/xcolor.sty
Package: xcolor 2023/11/15 v3.01 LaTeX color extensions (UK)
(c:/texlive/2024/texmf-dist/tex/latex/graphics-cfg/color.cfg
File: color.cfg 2016/01/02 v1.6 sample color configuration
)
Package xcolor Info: Driver file: pdftex.def on input line 274.
(c:/texlive/2024/texmf-dist/tex/latex/graphics-def/pdftex.def
File: pdftex.def 2024/04/13 v1.2c Graphics/color driver for pdftex
) (c:/texlive/2024/texmf-dist/tex/latex/graphics/mathcolor.ltx)
Package xcolor Info: Model `cmy' substituted by `cmy0' on input line
1350.
Package xcolor Info: Model `hsb' substituted by `rgb' on input line 1354.
Package xcolor Info: Model `RGB' extended on input line 1366.
Package xcolor Info: Model `HTML' substituted by `rgb' on input line
1368.
```

```
Package xcolor Info: Model `Hsb' substituted by `hsb' on input line 1369.
Package xcolor Info: Model `tHsb' substituted by `hsb' on input line
1370.
Package xcolor Info: Model `HSB' substituted by `hsb' on input line 1371.
Package xcolor Info: Model `Gray' substituted by `gray' on input line
1372.
Package xcolor Info: Model `wave' substituted by `hsb' on input line
1373.
) (c:/texlive/2024/texmf-dist/tex/latex/colortbl/colortbl.sty
Package: colortbl 2024/07/06 v1.0i Color table columns (DPC)
(c:/texlive/2024/texmf-dist/tex/latex/tools/array.sty
Package: array 2024/06/14 v2.6d Tabular extension package (FMi)
\col@sep=\dimen143
\ar@mcellbox=\box53
\extrarowheight=\dimen144
\NC@list=\toks28
\extratabsurround=\skip66
\backup@length=\skip67
\ar@cellbox=\box54
)
\everycr=\toks29
\minrowclearance=\skip68
\rownum=\count272
) (c:/texlive/2024/texmf-dist/tex/latex/graphics/graphicx.sty
Package: graphicx 2021/09/16 v1.2d Enhanced LaTeX Graphics (DPC,SPQR)
(c:/texlive/2024/texmf-dist/tex/latex/graphics/graphics.sty
Package: graphics 2024/05/23 v1.4g Standard LaTeX Graphics (DPC,SPQR)
(c:/texlive/2024/texmf-dist/tex/latex/graphics/trig.sty
Package: trig 2023/12/02 v1.11 sin cos tan (DPC)
) (c:/texlive/2024/texmf-dist/tex/latex/graphics-cfg/graphics.cfg
File: graphics.cfg 2016/06/04 v1.11 sample graphics configuration
)
Package graphics Info: Driver file: pdftex.def on input line 106.
)
\Gin@req@height=\dimen145
\Gin@req@width=\dimen146
) (c:/texlive/2024/texmf-dist/tex/latex/xpatch/xpatch.sty
(c:/texlive/2024/texm
f-dist/tex/latex/l3kernel/expl3.sty
Package: expl3 2024-05-27 L3 programming layer (loader)
(c:/texlive/2024/texmf-dist/tex/latex/l3backend/l3backend-pdftex.def
File: l3backend-pdftex.def 2024-05-08 L3 backend support: PDF output
(pdfTeX)
\l__color_backend_stack_int=\count273
\l__pdf_internal_box=\box55
))
Package: xpatch 2020/03/25 v0.3a Extending etoolbox patching commands
(c:/texlive/2024/texmf-dist/tex/latex/l3packages/xparse/xparse.sty
Package: xparse 2024-05-08 L3 Experimental document command parser
)) (c:/texlive/2024/texmf-dist/tex/latex/environ/environ.sty
Package: environ 2014/05/04 v0.3 A new way to define environments
(c:/texlive/2024/texmf-dist/tex/latex/trimspaces/trimspaces.sty
Package: trimspaces 2009/09/17 v1.1 Trim spaces around a token list
)
```

```
\@envbody=\toks30
) (c:/texlive/2024/texmf-dist/tex/latex/lastpage/lastpage.sty
Package: lastpage 2024/07/07 v2.1c lastpage: 2.09 or 2e? (HMM)
(c:/texlive/2024/texmf-dist/tex/latex/lastpage/lastpage2e.sty
Package: lastpage2e 2024/07/07 v2.1c Decide which 2e lastpage version to
use (H
MM)
(c:/texlive/2024/texmf-dist/tex/latex/lastpage/lastpagemodern.sty
Package: lastpagemodern 2024-07-07 v2.1c Refers to last page's name (HMM;
JPG)
\c@lastpagecount=\count274
)
)) (c:/texlive/2024/texmf-dist/tex/latex/graphics/rotating.sty
Package: rotating 2016/08/11 v2.16d rotated objects in LaTeX
(c:/texlive/2024/texmf-dist/tex/latex/base/ifthen.sty
Package: ifthen 2024/03/16 v1.1e Standard LaTeX ifthen package (DPC)
)
\c@r@tfl@t=\count275
\rotFPtop=\skip69
\rotFPbot=\skip70
\rot@float@box=\box56
\rot@mess@toks=\toks31
) (c:/texlive/2024/texmf-dist/tex/latex/graphics/lscape.sty
Package: lscape 2020/05/28 v3.02 Landscape Pages (DPC)
) (c:/texlive/2024/texmf-dist/tex/latex/tools/afterpage.sty
Package: afterpage 2023/07/04 v1.08 After-Page Package (DPC)
\AP@output=\toks32
\AP@partial=\box57
\AP@footins=\box58
) (c:/texlive/2024/texmf-dist/tex/latex/textpos/textpos.sty
Package: textpos 2022/07/23 v1.10.1
Package textpos Info: choosing support for LaTeX3 on input line 60.
\TP@textbox=\box59
\TP@holdbox=\box60
\TPHorizModule=\dimen147
\TPVertModule=\dimen148
\TP@margin=\dimen149
\TP@absmargin=\dimen150
Grid set 16 x 16 = 37.34424pt x 52.81541pt
\TPboxrulesize=\dimen151
\TP@ox=\dimen152
\TP@oy=\dimen153
\TP@tbargs=\toks33
TextBlockOrigin set to 0pt x 0pt
) (c:/texlive/2024/texmf-dist/tex/latex/url/url.sty
\Urlmuskip=\muskip20
Package: url 2013/09/16  ver 3.4  Verb mode for urls, etc.
) (c:/texlive/2024/texmf-dist/tex/latex/newfloat/newfloat.sty
Package: newfloat 2023/10/01 v1.2 Defining new floating environments (AR)
Package newfloat Info: `rotating' package detected.
) (c:/texlive/2024/texmf-dist/tex/latex/mdframed/mdframed.sty
Package: mdframed 2013/07/01 1.9b: mdframed
(c:/texlive/2024/texmf-dist/tex/latex/kvoptions/kvoptions.sty
```

```
Package: kvoptions 2022-06-15 v3.15 Key value format for package options
(HO)
(c:/texlive/2024/texmf-dist/tex/generic/ltxcmds/ltxcmds.sty
Package: ltxcmds 2023-12-04 v1.26 LaTeX kernel commands for general use
(HO)
) (c:/texlive/2024/texmf-dist/tex/latex/kvsetkeys/kvsetkeys.sty
Package: kvsetkeys 2022-10-05 v1.19 Key value parser (HO)
)) (c:/texlive/2024/texmf-dist/tex/latex/zref/zref-abspage.sty
Package: zref-abspage 2023-09-14 v2.35 Module abspage for zref (HO)
(c:/texlive/2024/texmf-dist/tex/latex/zref/zref-base.sty
Package: zref-base 2023-09-14 v2.35 Module base for zref (HO)
(c:/texlive/2024/texmf-dist/tex/generic/infwarerr/infwarerr.sty
Package: infwarerr 2019/12/03 v1.5 Providing info/warning/error messages
(HO)
) (c:/texlive/2024/texmf-dist/tex/generic/kvdefinekeys/kvdefinekeys.sty
Package: kvdefinekeys 2019-12-19 v1.6 Define keys (HO)
) (c:/texlive/2024/texmf-dist/tex/generic/pdftexcmds/pdftexcmds.sty
Package: pdftexcmds 2020-06-27 v0.33 Utility functions of pdfTeX for
LuaTeX (HO
)
Package pdftexcmds Info: \pdf@primitive is available.
Package pdftexcmds Info: \pdf@ifprimitive is available.
Package pdftexcmds Info: \pdfdraftmode found.
) (c:/texlive/2024/texmf-dist/tex/generic/etexcmds/etexcmds.sty
Package: etexcmds 2019/12/15 v1.7 Avoid name clashes with e-TeX commands
(HO)
) (c:/texlive/2024/texmf-dist/tex/latex/auxhook/auxhook.sty
Package: auxhook 2019-12-17 v1.6 Hooks for auxiliary files (HO)
)
Package zref Info: New property list: main on input line 767.
Package zref Info: New property: default on input line 768.
Package zref Info: New property: page on input line 769.
)
\c@abspage=\count276
Package zref Info: New property: abspage on input line 67.
) (c:/texlive/2024/texmf-dist/tex/latex/needspace/needspace.sty
Package: needspace 2010/09/12 v1.3d reserve vertical space
)
\mdf@templength=\skip71
\c@mdf@globalstyle@cnt=\count277
\mdf@skipabove@length=\skip72
\mdf@skipbelow@length=\skip73
\mdf@leftmargin@length=\skip74
\mdf@rightmargin@length=\skip75
\mdf@innerleftmargin@length=\skip76
\mdf@innerrightmargin@length=\skip77
\mdf@innertopmargin@length=\skip78
\mdf@innerbottommargin@length=\skip79
\mdf@splittopskip@length=\skip80
\mdf@splitbottomskip@length=\skip81
\mdf@outermargin@length=\skip82
\mdf@innermargin@length=\skip83
\mdf@linewidth@length=\skip84
\mdf@innerlinewidth@length=\skip85
```

```
\mdf@middlelinewidth@length=\skip86
\mdf@outerlinewidth@length=\skip87
\mdf@roundcorner@length=\skip88
\mdf@footenotedistance@length=\skip89
\mdf@userdefinedwidth@length=\skip90
\mdf@needspace@length=\skip91
\mdf@frametitleaboveskip@length=\skip92
\mdf@frametitlebelowskip@length=\skip93
\mdf@frametitlerulewidth@length=\skip94
\mdf@frametitleleftmargin@length=\skip95
\mdf@frametitlerightmargin@length=\skip96
\mdf@shadowsize@length=\skip97
\mdf@extratopheight@length=\skip98
\mdf@subtitleabovelinewidth@length=\skip99
\mdf@subtitlebelowlinewidth@length=\skip100
\mdf@subtitleaboveskip@length=\skip101
\mdf@subtitlebelowskip@length=\skip102
\mdf@subtitleinneraboveskip@length=\skip103
\mdf@subtitleinnerbelowskip@length=\skip104
\mdf@subsubtitleabovelinewidth@length=\skip105
\mdf@subsubtitlebelowlinewidth@length=\skip106
\mdf@subsubtitleaboveskip@length=\skip107
\mdf@subsubtitlebelowskip@length=\skip108
\mdf@subsubtitleinneraboveskip@length=\skip109
\mdf@subsubtitleinnerbelowskip@length=\skip110
(c:/texlive/2024/texmf-dist/tex/latex/mdframed/md-frame-0.mdf
File: md-frame-0.mdf 2013/07/01\ 1.9b: md-frame-0
)
\mdf@frametitlebox=\box61
\mdf@footnotebox=\box62
\mdf@splitbox@one=\box63
\mdf@splitbox@two=\box64
\mdf@splitbox@save=\box65
\mdfsplitboxwidth=\skip111
\mdfsplitboxtotalwidth=\skip112
\mdfsplitboxheight=\skip113
\mdfsplitboxdepth=\skip114
\mdfsplitboxtotalheight=\skip115
\mdfframetitleboxwidth=\skip116
\mdfframetitleboxtotalwidth=\skip117
\mdfframetitleboxheight=\skip118
\mdfframetitleboxdepth=\skip119
\mdfframetitleboxtotalheight=\skip120
\mdffootnoteboxwidth=\skip121
\mdffootnoteboxtotalwidth=\skip122
\mdffootnoteboxheight=\skip123
\mdffootnoteboxdepth=\skip124
\mdffootnoteboxtotalheight=\skip125
\mdftotallinewidth=\skip126
\mdfboundingboxwidth=\skip127
\mdfboundingboxtotalwidth=\skip128
\mdfboundingboxheight=\skip129
\mdfboundingboxdepth=\skip130
\mdfboundingboxtotalheight=\skip131
```

```
\mdf@freevspace@length=\skip132
\mdf@horizontalwidthofbox@length=\skip133
\mdf@verticalmarginwhole@length=\skip134
\mdf@horizontalspaceofbox=\skip135
\mdfsubtitleheight=\skip136
\mdfsubsubtitleheight=\skip137
\c@mdfcountframes=\count278

****** mdframed patching \endmdf@trivlist

****** -- success******

\mdf@envdepth=\count279
\c@mdf@env@i=\count280
\c@mdf@env@ii=\count281
\c@mdf@zref@counter=\count282
Package zref Info: New property: mdf@pagevalue on input line 895.
) (c:/texlive/2024/texmf-dist/tex/latex/titlesec/titlesec.sty
Package: titlesec 2023/10/27 v2.16 Sectioning titles
\ttl@box=\box66
\beforetitleunit=\skip138
\aftertitleunit=\skip139
\ttl@plus=\dimen154
\ttl@minus=\dimen155
\ttl@toksa=\toks34
\titlewidth=\dimen156
\titlewidthlast=\dimen157
\titlewidthfirst=\dimen158
) (c:/texlive/2024/texmf-dist/tex/latex/koma-script/scrextend.sty
Package: scrextend 2023/07/07 v3.41 KOMA-Script package (extend other
classes w
ith features of KOMA-Script classes)
(c:/texlive/2024/texmf-dist/tex/latex/koma-script/scrkbase.sty
Package: scrkbase 2023/07/07 v3.41 KOMA-Script package (KOMA-Script-
dependent b
asics and keyval usage)
(c:/texlive/2024/texmf-dist/tex/latex/koma-script/scrbase.sty
Package: scrbase 2023/07/07 v3.41 KOMA-Script package (KOMA-Script-
independent
basics and keyval usage)
(c:/texlive/2024/texmf-dist/tex/latex/koma-script/scrlfile.sty
Package: scrlfile 2023/07/07 v3.41 KOMA-Script package (file load hooks)
(c:/texlive/2024/texmf-dist/tex/latex/koma-script/scrlfile-hook.sty
Package: scrlfile-hook 2023/07/07 v3.41 KOMA-Script package (using LaTeX
hooks)

(c:/texlive/2024/texmf-dist/tex/latex/koma-script/scrlogo.sty
Package: scrlogo 2023/07/07 v3.41 KOMA-Script package (logo)
)))
Applying: [2021/05/01] Usage of raw or classic option list on input line
252.
Already applied: [0000/00/00] Usage of raw or classic option list on
input line
 368.
```

```
))
Package scrextend Info: unexpected definition of `\@makefnmark'.
(scrextend)             Trying to patch it on input line 1762.
Package scrextend Info: patch seems to be successfull on input line 1762.
)

LaTeX Font Warning: Font shape `T1/cmr/m/n' in size <7.5> not available
(Font)              size <7> substituted on input line 69.

(c:/texlive/2024/texmf-dist/tex/latex/tools/calc.sty
Package: calc 2023/07/08 v4.3 Infix arithmetic (KKT,FJ)
\calc@Acount=\count283
\calc@Bcount=\count284
\calc@Adimen=\dimen159
\calc@Bdimen=\dimen160
\calc@Askip=\skip140
\calc@Bskip=\skip141
LaTeX Info: Redefining \setlength on input line 80.
LaTeX Info: Redefining \addtolength on input line 81.
\calc@Ccount=\count285
\calc@Cskip=\skip142
) (c:/texlive/2024/texmf-dist/tex/latex/geometry/geometry.sty
Package: geometry 2020/01/02 v5.9 Page Geometry
(c:/texlive/2024/texmf-dist/tex/generic/iftex/ifvtex.sty
Package: ifvtex 2019/10/25 v1.7 ifvtex legacy package. Use iftex instead.
)
\Gm@cnth=\count286
\Gm@cntv=\count287
\c@Gm@tempcnt=\count288
\Gm@bindingoffset=\dimen161
\Gm@wd@mp=\dimen162
\Gm@odd@mp=\dimen163
\Gm@even@mp=\dimen164
\Gm@layoutwidth=\dimen165
\Gm@layoutheight=\dimen166
\Gm@layouthoffset=\dimen167
\Gm@layoutvoffset=\dimen168
\Gm@dimlist=\toks35
) (c:/texlive/2024/texmf-dist/tex/latex/preprint/authblk.sty
Package: authblk 2001/02/27 1.3 (PWD)
\affilsep=\skip143
\@affilsep=\skip144
\c@Maxaffil=\count289
\c@authors=\count290
\c@affil=\count291
) (c:/texlive/2024/texmf-dist/tex/latex/footmisc/footmisc.sty
Package: footmisc 2023/07/05 v6.0f a miscellany of footnote facilities
\FN@temptoken=\toks36
\footnotemargin=\dimen169
\@outputbox@depth=\dimen170
Package footmisc Info: Declaring symbol style bringhurst on input line
696.
Package footmisc Info: Declaring symbol style chicago on input line 704.
Package footmisc Info: Declaring symbol style wiley on input line 713.
```

```
Package footmisc Info: Declaring symbol style lamport-robust on input
line 724.

Package footmisc Info: Declaring symbol style lamport* on input line 744.
Package footmisc Info: Declaring symbol style lamport*-robust on input
line 765
.
) (c:/texlive/2024/texmf-dist/tex/latex/fancyhdr/fancyhdr.sty
Package: fancyhdr 2024/07/23 v4.3.1 Extensive control of page headers and
foote
rs
\f@nch@headwidth=\skip145
\f@nch@O@elh=\skip146
\f@nch@O@erh=\skip147
\f@nch@O@olh=\skip148
\f@nch@O@orh=\skip149
\f@nch@O@elf=\skip150
\f@nch@O@erf=\skip151
\f@nch@O@olf=\skip152
\f@nch@O@orf=\skip153
) (c:/texlive/2024/texmf-dist/tex/generic/alphalph/alphalph.sty
Package: alphalph 2019/12/09 v2.6 Convert numbers to letters (HO)
(c:/texlive/2024/texmf-dist/tex/generic/intcalc/intcalc.sty
Package: intcalc 2019/12/15 v1.3 Expandable calculations with integers
(HO)
))
\c@authorfn=\count292
(c:/texlive/2024/texmf-dist/tex/latex/abstract/abstract.sty
Package: abstract 2009/06/08 v1.2a configurable abstracts
\abstitleskip=\skip154
\absleftindent=\skip155
\absrightindent=\skip156
\absparindent=\skip157
\absparsep=\skip158
)
Package newfloat Info: New float `keypoints' with options
`placement=t!,name=kp
t' on input line 291.
\c@keypoints=\count293
\newfloat@ftype=\count294
Package newfloat Info: float type `keypoints'=8 on input line 291.
(c:/texlive/2024/texmf-dist/tex/latex/enumitem/enumitem.sty
Package: enumitem 2019/06/20 v3.9 Customized lists
\labelindent=\skip159
\enit@outerparindent=\dimen171
\enit@toks=\toks37
\enit@inbox=\box67
\enit@count@id=\count295
\enitdp@description=\count296
) (c:/texlive/2024/texmf-dist/tex/latex/quoting/quoting.sty
Package: quoting 2014/01/28 v0.1c Consolidated environment for displayed
text
\quo@toppartop=\skip160
) (c:/texlive/2024/texmf-dist/tex/latex/sttools/stfloats.sty
```

```
Package: stfloats 2017/03/27 v3.3 Improve float mechanism and
baselineskip sett
ings
\@dblbotnum=\count297
\c@dblbotnumber=\count298
) (c:/texlive/2024/texmf-dist/tex/latex/booktabs/booktabs.sty
Package: booktabs 2020/01/12 v1.61803398 Publication quality tables
\heavyrulewidth=\dimen172
\lightrulewidth=\dimen173
\cmidrulewidth=\dimen174
\belowrulesep=\dimen175
\belowbottomsep=\dimen176
\aboverulesep=\dimen177
\abovetopsep=\dimen178
\cmidrulesep=\dimen179
\cmidrulekern=\dimen180
\defaultaddspace=\dimen181
\@cmidla=\count299
\@cmidlb=\count300
\@aboverulesep=\dimen182
\@belowrulesep=\dimen183
\@thisruleclass=\count301
\@lastruleclass=\count302
\@thisrulewidth=\dimen184
) (c:/texlive/2024/texmf-dist/tex/latex/tools/tabularx.sty
Package: tabularx 2023/12/11 v2.12a `tabularx' package (DPC)
\TX@col@width=\dimen185
\TX@old@table=\dimen186
\TX@old@col=\dimen187
\TX@target=\dimen188
\TX@delta=\dimen189
\TX@cols=\count303
\TX@ftn=\toks38
)
\enitdp@tablenotes=\count304
(c:/texlive/2024/texmf-dist/tex/latex/caption/caption.sty
Package: caption 2023/08/05 v3.6o Customizing captions (AR)
(c:/texlive/2024/texmf-dist/tex/latex/caption/caption3.sty
Package: caption3 2023/07/31 v2.4d caption3 kernel (AR)
\caption@tempdima=\dimen190
\captionmargin=\dimen191
\caption@leftmargin=\dimen192
\caption@rightmargin=\dimen193
\caption@width=\dimen194
\caption@indent=\dimen195
\caption@parindent=\dimen196
\caption@hangindent=\dimen197
Package caption Info: Standard document class detected.
)
\c@caption@flags=\count305
\c@continuedfloat=\count306
Package caption Info: rotating package is loaded.
Package caption Info: scrextend package is loaded.
\caption@addmargin@hsize=\dimen198
```

```
\caption@addmargin@linewidth=\dimen199
) (c:/texlive/2024/texmf-dist/tex/latex/natbib/natbib.sty
Package: natbib 2010/09/13 8.31b (PWD, AO)
\bibhang=\skip161
\bibsep=\skip162
LaTeX Info: Redefining \cite on input line 694.
\c@NAT@ctr=\count307
)) (c:/texlive/2024/texmf-dist/tex/latex/siunitx/siunitx.sty
Package: siunitx 2024-06-24 v3.3.19 A comprehensive (SI) units package
\l__siunitx_number_uncert_offset_int=\count308
\l__siunitx_number_exponent_fixed_int=\count309
\l__siunitx_number_min_decimal_int=\count310
\l__siunitx_number_min_integer_int=\count311
\l__siunitx_number_round_precision_int=\count312
\l__siunitx_number_lower_threshold_int=\count313
\l__siunitx_number_upper_threshold_int=\count314
\l__siunitx_number_group_first_int=\count315
\l__siunitx_number_group_size_int=\count316
\l__siunitx_number_group_minimum_int=\count317
\l__siunitx_angle_tmp_dim=\dimen256
\l__siunitx_angle_marker_box=\box68
\l__siunitx_angle_unit_box=\box69
\l__siunitx_compound_count_int=\count318
(c:/texlive/2024/texmf-dist/tex/latex/translations/translations.sty
Package: translations 2022/02/05 v1.12 internationalization of LaTeX2e
packages
 (CN)
) (c:/texlive/2024/texmf-dist/tex/latex/amsmath/amstext.sty
Package: amstext 2021/08/26 v2.01 AMS text
(c:/texlive/2024/texmf-dist/tex/latex/amsmath/amsgen.sty
File: amsgen.sty 1999/11/30 v2.0 generic functions
\@emptytoks=\toks39
\ex@=\dimen257
))
\l__siunitx_table_tmp_box=\box70
\l__siunitx_table_tmp_dim=\dimen258
\l__siunitx_table_column_width_dim=\dimen259
\l__siunitx_table_integer_box=\box71
\l__siunitx_table_decimal_box=\box72
\l__siunitx_table_uncert_box=\box73
\l__siunitx_table_before_box=\box74
\l__siunitx_table_after_box=\box75
\l__siunitx_table_before_dim=\dimen260
\l__siunitx_table_carry_dim=\dimen261
\l__siunitx_unit_tmp_int=\count319
\l__siunitx_unit_position_int=\count320
\l__siunitx_unit_total_int=\count321
) (c:/texlive/2024/texmf-dist/tex/latex/orcidlink/orcidlink.sty
Package: orcidlink 2024/06/26 v1.1.0 Support ORCID's three different ID
formats
.
(c:/texlive/2024/texmf-dist/tex/latex/hyperref/hyperref.sty
Package: hyperref 2024-07-10 v7.01j Hypertext links for LaTeX
(c:/texlive/2024/texmf-dist/tex/generic/pdfescape/pdfescape.sty
```

```
Package: pdfescape 2019/12/09 v1.15 Implements pdfTeX's escape features
(HO)
) (c:/texlive/2024/texmf-dist/tex/latex/hycolor/hycolor.sty
Package: hycolor 2020-01-27 v1.10 Color options for hyperref/bookmark
(HO)
) (c:/texlive/2024/texmf-dist/tex/latex/hyperref/nameref.sty
Package: nameref 2023-11-26 v2.56 Cross-referencing by name of section
(c:/texlive/2024/texmf-dist/tex/latex/refcount/refcount.sty
Package: refcount 2019/12/15 v3.6 Data extraction from label references
(HO)
) (c:/texlive/2024/texmf-
dist/tex/generic/gettitlestring/gettitlestring.sty
Package: gettitlestring 2019/12/15 v1.6 Cleanup title references (HO)
)
\c@section@level=\count322
) (c:/texlive/2024/texmf-dist/tex/generic/stringenc/stringenc.sty
Package: stringenc 2019/11/29 v1.12 Convert strings between diff.
encodings (HO
)
)
\@linkdim=\dimen262
\Hy@linkcounter=\count323
\Hy@pagecounter=\count324
(c:/texlive/2024/texmf-dist/tex/latex/hyperref/pd1enc.def
File: pd1enc.def 2024-07-10 v7.01j Hyperref: PDFDocEncoding definition
(HO)
Now handling font encoding PD1 ...
... no UTF-8 mapping file for font encoding PD1
)
\Hy@SavedSpaceFactor=\count325
(c:/texlive/2024/texmf-dist/tex/latex/hyperref/puenc.def
File: puenc.def 2024-07-10 v7.01j Hyperref: PDF Unicode definition (HO)
Now handling font encoding PU ...
... no UTF-8 mapping file for font encoding PU
)
Package hyperref Info: Hyper figures OFF on input line 4157.
Package hyperref Info: Link nesting OFF on input line 4162.
Package hyperref Info: Hyper index ON on input line 4165.
Package hyperref Info: Plain pages OFF on input line 4172.
Package hyperref Info: Backreferencing OFF on input line 4177.
Package hyperref Info: Implicit mode ON; LaTeX internals redefined.
Package hyperref Info: Bookmarks ON on input line 4424.
\c@Hy@tempcnt=\count326
LaTeX Info: Redefining \url on input line 4763.
\XeTeXLinkMargin=\dimen263
(c:/texlive/2024/texmf-dist/tex/generic/bitset/bitset.sty
Package: bitset 2019/12/09 v1.3 Handle bit-vector datatype (HO)
(c:/texlive/2024/texmf-dist/tex/generic/bigintcalc/bigintcalc.sty
Package: bigintcalc 2019/12/15 v1.5 Expandable calculations on big
integers (HO
)
))
\Fld@menulength=\count327
\Field@Width=\dimen264
```

```
\Fld@charsize=\dimen265
Package hyperref Info: Hyper figures OFF on input line 6042.
Package hyperref Info: Link nesting OFF on input line 6047.
Package hyperref Info: Hyper index ON on input line 6050.
Package hyperref Info: backreferencing OFF on input line 6057.
Package hyperref Info: Link coloring OFF on input line 6062.
Package hyperref Info: Link coloring with OCG OFF on input line 6067.
Package hyperref Info: PDF/A mode OFF on input line 6072.
(c:/texlive/2024/texmf-dist/tex/latex/base/atbegshi-ltx.sty
Package: atbegshi-ltx 2021/01/10 v1.0c Emulation of the original atbegshi
package with kernel methods
)
\Hy@abspage=\count328
\c@Item=\count329
\c@Hfootnote=\count330
)
Package hyperref Info: Driver (autodetected): hpdftex.
(c:/texlive/2024/texmf-dist/tex/latex/hyperref/hpdftex.def
File: hpdftex.def 2024-07-10 v7.01j Hyperref driver for pdfTeX
(c:/texlive/2024/texmf-dist/tex/latex/base/atveryend-ltx.sty
Package: atveryend-ltx 2020/08/19 v1.0a Emulation of the original
atveryend pac
kage
with kernel methods
)
\HyAnn@Count=\count331
\Fld@listcount=\count332
\c@bookmark@seq@number=\count333
(c:/texlive/2024/texmf-dist/tex/latex/rerunfilecheck/rerunfilecheck.sty
Package: rerunfilecheck 2022-07-10 v1.10 Rerun checks for auxiliary files
(HO)
(c:/texlive/2024/texmf-dist/tex/generic/uniquecounter/uniquecounter.sty
Package: uniquecounter 2019/12/15 v1.4 Provide unlimited unique counter
(HO)
)
Package uniquecounter Info: New unique counter `rerunfilecheck' on input
line 2
85.
)
\Hy@SectionHShift=\skip163
) (c:/texlive/2024/texmf-dist/tex/latex/pgf/frontendlayer/tikz.sty
(c:/texlive/
2024/texmf-dist/tex/latex/pgf/basiclayer/pgf.sty (c:/texlive/2024/texmf-
dist/te
x/latex/pgf/utilities/pgfrcs.sty (c:/texlive/2024/texmf-
dist/tex/generic/pgf/ut
ilities/pgfutil-common.tex
\pgfutil@everybye=\toks40
\pgfutil@tempdima=\dimen266
\pgfutil@tempdimb=\dimen267
) (c:/texlive/2024/texmf-dist/tex/generic/pgf/utilities/pgfutil-latex.def
\pgfutil@abb=\box76
) (c:/texlive/2024/texmf-dist/tex/generic/pgf/utilities/pgfrcs.code.tex
(c:/tex
```

```
live/2024/texmf-dist/tex/generic/pgf/pgf.revision.tex)
Package: pgfrcs 2023-01-15 v3.1.10 (3.1.10)
))
Package: pgf 2023-01-15 v3.1.10 (3.1.10)
(c:/texlive/2024/texmf-dist/tex/latex/pgf/basiclayer/pgfcore.sty
(c:/texlive/20
24/texmf-dist/tex/latex/pgf/systemlayer/pgfsys.sty
(c:/texlive/2024/texmf-dist/
tex/generic/pgf/systemlayer/pgfsys.code.tex
Package: pgfsys 2023-01-15 v3.1.10 (3.1.10)
(c:/texlive/2024/texmf-dist/tex/generic/pgf/utilities/pgfkeys.code.tex
\pgfkeys@pathtoks=\toks41
\pgfkeys@temptoks=\toks42

(c:/texlive/2024/texmf-
dist/tex/generic/pgf/utilities/pgfkeyslibraryfiltered.co
de.tex
\pgfkeys@tmptoks=\toks43
))
\pgf@x=\dimen268
\pgf@y=\dimen269
\pgf@xa=\dimen270
\pgf@ya=\dimen271
\pgf@xb=\dimen272
\pgf@yb=\dimen273
\pgf@xc=\dimen274
\pgf@yc=\dimen275
\pgf@xd=\dimen276
\pgf@yd=\dimen277
\w@pgf@writea=\write3
\r@pgf@reada=\read2
\c@pgf@counta=\count334
\c@pgf@countb=\count335
\c@pgf@countc=\count336
\c@pgf@countd=\count337
\t@pgf@toka=\toks44
\t@pgf@tokb=\toks45
\t@pgf@tokc=\toks46
\pgf@sys@id@count=\count338
(c:/texlive/2024/texmf-dist/tex/generic/pgf/systemlayer/pgf.cfg
File: pgf.cfg 2023-01-15 v3.1.10 (3.1.10)
)
Driver file for pgf: pgfsys-pdftex.def
(c:/texlive/2024/texmf-dist/tex/generic/pgf/systemlayer/pgfsys-pdftex.def
File: pgfsys-pdftex.def 2023-01-15 v3.1.10 (3.1.10)
(c:/texlive/2024/texmf-dist/tex/generic/pgf/systemlayer/pgfsys-common-
pdf.def
File: pgfsys-common-pdf.def 2023-01-15 v3.1.10 (3.1.10)
)))
(c:/texlive/2024/texmf-
dist/tex/generic/pgf/systemlayer/pgfsyssoftpath.code.tex
File: pgfsyssoftpath.code.tex 2023-01-15 v3.1.10 (3.1.10)
\pgfsyssoftpath@smallbuffer@items=\count339
\pgfsyssoftpath@bigbuffer@items=\count340
```

```
)
(c:/texlive/2024/texmf-
dist/tex/generic/pgf/systemlayer/pgfsysprotocol.code.tex
File: pgfsysprotocol.code.tex 2023-01-15 v3.1.10 (3.1.10)
)) (c:/texlive/2024/texmf-
dist/tex/generic/pgf/basiclayer/pgfcore.code.tex
Package: pgfcore 2023-01-15 v3.1.10 (3.1.10)
(c:/texlive/2024/texmf-dist/tex/generic/pgf/math/pgfmath.code.tex
(c:/texlive/2
024/texmf-dist/tex/generic/pgf/math/pgfmathutil.code.tex)
(c:/texlive/2024/texm
f-dist/tex/generic/pgf/math/pgfmathparser.code.tex
\pgfmath@dimen=\dimen278
\pgfmath@count=\count341
\pgfmath@box=\box77
\pgfmath@toks=\toks47
\pgfmath@stack@operand=\toks48
\pgfmath@stack@operation=\toks49
) (c:/texlive/2024/texmf-
dist/tex/generic/pgf/math/pgfmathfunctions.code.tex)
(c:/texlive/2024/texmf-
dist/tex/generic/pgf/math/pgfmathfunctions.basic.code.te
x)
(c:/texlive/2024/texmf-
dist/tex/generic/pgf/math/pgfmathfunctions.trigonometric
.code.tex)
(c:/texlive/2024/texmf-
dist/tex/generic/pgf/math/pgfmathfunctions.random.code.t
ex)
(c:/texlive/2024/texmf-
dist/tex/generic/pgf/math/pgfmathfunctions.comparison.co
de.tex)
(c:/texlive/2024/texmf-
dist/tex/generic/pgf/math/pgfmathfunctions.base.code.tex
)
(c:/texlive/2024/texmf-
dist/tex/generic/pgf/math/pgfmathfunctions.round.code.te
x)
(c:/texlive/2024/texmf-
dist/tex/generic/pgf/math/pgfmathfunctions.misc.code.tex
)
(c:/texlive/2024/texmf-
dist/tex/generic/pgf/math/pgfmathfunctions.integerarithm
etics.code.tex) (c:/texlive/2024/texmf-
dist/tex/generic/pgf/math/pgfmathcalc.co
de.tex) (c:/texlive/2024/texmf-
dist/tex/generic/pgf/math/pgfmathfloat.code.tex
\c@pgfmathroundto@lastzeros=\count342
)) (c:/texlive/2024/texmf-dist/tex/generic/pgf/math/pgfint.code.tex)
(c:/texliv
e/2024/texmf-dist/tex/generic/pgf/basiclayer/pgfcorepoints.code.tex
File: pgfcorepoints.code.tex 2023-01-15 v3.1.10 (3.1.10)
\pgf@picminx=\dimen279
\pgf@picmaxx=\dimen280
```

```
\pgf@picminy=\dimen281
\pgf@picmaxy=\dimen282
\pgf@pathminx=\dimen283
\pgf@pathmaxx=\dimen284
\pgf@pathminy=\dimen285
\pgf@pathmaxy=\dimen286
\pgf@xx=\dimen287
\pgf@xy=\dimen288
\pgf@yx=\dimen289
\pgf@yy=\dimen290
\pgf@zx=\dimen291
\pgf@zy=\dimen292
)
(c:/texlive/2024/texmf-
dist/tex/generic/pgf/basiclayer/pgfcorepathconstruct.cod
e.tex
File: pgfcorepathconstruct.code.tex 2023-01-15 v3.1.10 (3.1.10)
\pgf@path@lastx=\dimen293
\pgf@path@lasty=\dimen294
)
(c:/texlive/2024/texmf-
dist/tex/generic/pgf/basiclayer/pgfcorepathusage.code.te
x
File: pgfcorepathusage.code.tex 2023-01-15 v3.1.10 (3.1.10)
\pgf@shorten@end@additional=\dimen295
\pgf@shorten@start@additional=\dimen296
) (c:/texlive/2024/texmf-
dist/tex/generic/pgf/basiclayer/pgfcorescopes.code.tex
File: pgfcorescopes.code.tex 2023-01-15 v3.1.10 (3.1.10)
\pgfpic=\box78
\pgf@hbox=\box79
\pgf@layerbox@main=\box80
\pgf@picture@serial@count=\count343
)
(c:/texlive/2024/texmf-
dist/tex/generic/pgf/basiclayer/pgfcoregraphicstate.code
.tex
File: pgfcoregraphicstate.code.tex 2023-01-15 v3.1.10 (3.1.10)
\pgflinewidth=\dimen297
)
(c:/texlive/2024/texmf-
dist/tex/generic/pgf/basiclayer/pgfcoretransformations.c
ode.tex
File: pgfcoretransformations.code.tex 2023-01-15 v3.1.10 (3.1.10)
\pgf@pt@x=\dimen298
\pgf@pt@y=\dimen299
\pgf@pt@temp=\dimen300
) (c:/texlive/2024/texmf-
dist/tex/generic/pgf/basiclayer/pgfcorequick.code.tex
File: pgfcorequick.code.tex 2023-01-15 v3.1.10 (3.1.10)
) (c:/texlive/2024/texmf-
dist/tex/generic/pgf/basiclayer/pgfcoreobjects.code.te
x
File: pgfcoreobjects.code.tex 2023-01-15 v3.1.10 (3.1.10)
```

```
)
(c:/texlive/2024/texmf-
dist/tex/generic/pgf/basiclayer/pgfcorepathprocessing.co
de.tex
File: pgfcorepathprocessing.code.tex 2023-01-15 v3.1.10 (3.1.10)
) (c:/texlive/2024/texmf-
dist/tex/generic/pgf/basiclayer/pgfcorearrows.code.tex
File: pgfcorearrows.code.tex 2023-01-15 v3.1.10 (3.1.10)
\pgfarrowsep=\dimen301
) (c:/texlive/2024/texmf-
dist/tex/generic/pgf/basiclayer/pgfcoreshade.code.tex
File: pgfcoreshade.code.tex 2023-01-15 v3.1.10 (3.1.10)
\pgf@max=\dimen302
\pgf@sys@shading@range@num=\count344
\pgf@shadingcount=\count345
) (c:/texlive/2024/texmf-
dist/tex/generic/pgf/basiclayer/pgfcoreimage.code.tex
File: pgfcoreimage.code.tex 2023-01-15 v3.1.10 (3.1.10)
)
(c:/texlive/2024/texmf-
dist/tex/generic/pgf/basiclayer/pgfcoreexternal.code.tex
File: pgfcoreexternal.code.tex 2023-01-15 v3.1.10 (3.1.10)
\pgfexternal@startupbox=\box81
) (c:/texlive/2024/texmf-
dist/tex/generic/pgf/basiclayer/pgfcorelayers.code.tex
File: pgfcorelayers.code.tex 2023-01-15 v3.1.10 (3.1.10)
)
(c:/texlive/2024/texmf-
dist/tex/generic/pgf/basiclayer/pgfcoretransparency.code
.tex
File: pgfcoretransparency.code.tex 2023-01-15 v3.1.10 (3.1.10)
)
(c:/texlive/2024/texmf-
dist/tex/generic/pgf/basiclayer/pgfcorepatterns.code.tex
File: pgfcorepatterns.code.tex 2023-01-15 v3.1.10 (3.1.10)
) (c:/texlive/2024/texmf-
dist/tex/generic/pgf/basiclayer/pgfcorerdf.code.tex
File: pgfcorerdf.code.tex 2023-01-15 v3.1.10 (3.1.10)
))) (c:/texlive/2024/texmf-
dist/tex/generic/pgf/modules/pgfmoduleshapes.code.te
x
File: pgfmoduleshapes.code.tex 2023-01-15 v3.1.10 (3.1.10)
\pgfnodeparttextbox=\box82
) (c:/texlive/2024/texmf-
dist/tex/generic/pgf/modules/pgfmoduleplot.code.tex
File: pgfmoduleplot.code.tex 2023-01-15 v3.1.10 (3.1.10)
)
(c:/texlive/2024/texmf-dist/tex/latex/pgf/compatibility/pgfcomp-version-
0-65.st
y
Package: pgfcomp-version-0-65 2023-01-15 v3.1.10 (3.1.10)
\pgf@nodesepstart=\dimen303
\pgf@nodesepend=\dimen304
)
```

```
(c:/texlive/2024/texmf-dist/tex/latex/pgf/compatibility/pgfcomp-version-
1-18.st
y
Package: pgfcomp-version-1-18 2023-01-15 v3.1.10 (3.1.10)
)) (c:/texlive/2024/texmf-dist/tex/latex/pgf/utilities/pgffor.sty
(c:/texlive/2
024/texmf-dist/tex/latex/pgf/utilities/pgfkeys.sty
(c:/texlive/2024/texmf-dist/
tex/generic/pgf/utilities/pgfkeys.code.tex)) (c:/texlive/2024/texmf-
dist/tex/la
tex/pgf/math/pgfmath.sty (c:/texlive/2024/texmf-
dist/tex/generic/pgf/math/pgfma
th.code.tex)) (c:/texlive/2024/texmf-
dist/tex/generic/pgf/utilities/pgffor.code
.tex
Package: pgffor 2023-01-15 v3.1.10 (3.1.10)
\pgffor@iter=\dimen305
\pgffor@skip=\dimen306
\pgffor@stack=\toks50
\pgffor@toks=\toks51
)) (c:/texlive/2024/texmf-
dist/tex/generic/pgf/frontendlayer/tikz/tikz.code.tex
Package: tikz 2023-01-15 v3.1.10 (3.1.10)

(c:/texlive/2024/texmf-
dist/tex/generic/pgf/libraries/pgflibraryplothandlers.co
de.tex
File: pgflibraryplothandlers.code.tex 2023-01-15 v3.1.10 (3.1.10)
\pgf@plot@mark@count=\count346
\pgfplotmarksize=\dimen307
)
\tikz@lastx=\dimen308
\tikz@lasty=\dimen309
\tikz@lastxsaved=\dimen310
\tikz@lastysaved=\dimen311
\tikz@lastmovetox=\dimen312
\tikz@lastmovetoy=\dimen313
\tikzleveldistance=\dimen314
\tikzsiblingdistance=\dimen315
\tikz@figbox=\box83
\tikz@figbox@bg=\box84
\tikz@tempbox=\box85
\tikz@tempbox@bg=\box86
\tikztreelevel=\count347
\tikznumberofchildren=\count348
\tikznumberofcurrentchild=\count349
\tikz@fig@count=\count350
(c:/texlive/2024/texmf-
dist/tex/generic/pgf/modules/pgfmodulematrix.code.tex
File: pgfmodulematrix.code.tex 2023-01-15 v3.1.10 (3.1.10)
\pgfmatrixcurrentrow=\count351
\pgfmatrixcurrentcolumn=\count352
\pgf@matrix@numberofcolumns=\count353
)
```

```
\tikz@expandcount=\count354

(c:/texlive/2024/texmf-
dist/tex/generic/pgf/frontendlayer/tikz/libraries/tikzli
brarytopaths.code.tex
File: tikzlibrarytopaths.code.tex 2023-01-15 v3.1.10 (3.1.10)
)))
(c:/texlive/2024/texmf-
dist/tex/generic/pgf/frontendlayer/tikz/libraries/tikzli
brarysvg.path.code.tex
File: tikzlibrarysvg.path.code.tex 2023-01-15 v3.1.10 (3.1.10)

(c:/texlive/2024/texmf-
dist/tex/generic/pgf/libraries/pgflibrarysvg.path.code.t
ex
File: pgflibrarysvg.path.code.tex 2023-01-15 v3.1.10 (3.1.10)
(c:/texlive/2024/texmf-
dist/tex/generic/pgf/modules/pgfmoduleparser.code.tex
File: pgfmoduleparser.code.tex 2023-01-15 v3.1.10 (3.1.10)
\pgfparserdef@arg@count=\count355
)
\pgf@lib@svg@last@x=\dimen316
\pgf@lib@svg@last@y=\dimen317
\pgf@lib@svg@last@c@x=\dimen318
\pgf@lib@svg@last@c@y=\dimen319
\pgf@lib@svg@count=\count356
\pgf@lib@svg@max@num=\count357
))
\@curXheight=\skip164
)

! LaTeX Error: Option clash for package hyperref.

See the LaTeX manual or LaTeX Companion for explanation.
Type  H <return>  for immediate help.
 ...

l.47 \begin{document}

The package hyperref has already been loaded with options:
  []
There has now been an attempt to load it with options
  [colorlinks,allcolors=black,urlcolor=blue]
Adding the global options:
  ,colorlinks,allcolors=black,urlcolor=blue
to your \documentclass declaration may fix this.
Try typing  <return>  to proceed.

Package translations Info: No language package found. I am going to use
`englis
h' as default language. on input line 47.
LaTeX Font Info:    Trying to load font information for T1+Merriwthr-OsF
on inp
ut line 47.
```

```
(c:/texlive/2024/texmf-dist/tex/latex/merriweather/T1Merriwthr-OsF.fd
File: T1Merriwthr-OsF.fd 2020/08/30 (autoinst) Font definitions for
T1/Merriwth
r-OsF.
)
LaTeX Font Info:    Font shape `T1/Merriwthr-OsF/m/n' will be
(Font)              scaled to size 7.5pt on input line 47.
(./main.aux)
\openout1 = `main.aux'.

LaTeX Font Info:    Checking defaults for OML/cmm/m/it on input line 47.
LaTeX Font Info:    ... okay on input line 47.
LaTeX Font Info:    Checking defaults for OMS/cmsy/m/n on input line 47.
LaTeX Font Info:    ... okay on input line 47.
LaTeX Font Info:    Checking defaults for OT1/cmr/m/n on input line 47.
LaTeX Font Info:    ... okay on input line 47.
LaTeX Font Info:    Checking defaults for T1/cmr/m/n on input line 47.
LaTeX Font Info:    ... okay on input line 47.
LaTeX Font Info:    Checking defaults for TS1/cmr/m/n on input line 47.
LaTeX Font Info:    ... okay on input line 47.
LaTeX Font Info:    Checking defaults for OMX/cmex/m/n on input line 47.
LaTeX Font Info:    ... okay on input line 47.
LaTeX Font Info:    Checking defaults for U/cmr/m/n on input line 47.
LaTeX Font Info:    ... okay on input line 47.
LaTeX Font Info:    Checking defaults for PD1/pdf/m/n on input line 47.
LaTeX Font Info:    ... okay on input line 47.
LaTeX Font Info:    Checking defaults for PU/pdf/m/n on input line 47.
LaTeX Font Info:    ... okay on input line 47.
LaTeX Info: Redefining \microtypecontext on input line 47.
Package microtype Info: Applying patch `item' on input line 47.
Package microtype Info: Applying patch `toc' on input line 47.
Package microtype Info: Applying patch `eqnum' on input line 47.
Package microtype Info: Applying patch `footnote' on input line 47.
Package microtype Info: Applying patch `verbatim' on input line 47.
Package microtype Info: Generating PDF output.
Package microtype Info: Character protrusion enabled (level 2).
Package microtype Info: Using default protrusion set `alltext'.
Package microtype Info: Automatic font expansion enabled (level 2),
(microtype)             stretch: 20, shrink: 20, step: 1, non-selected.
Package microtype Info: Using default expansion set `alltext-nott'.
LaTeX Info: Redefining \showhyphens on input line 47.
Package microtype Info: No adjustment of tracking.
Package microtype Info: No adjustment of interword spacing.
Package microtype Info: No adjustment of character kerning.
Package microtype Info: Loading generic protrusion settings for font
family
(microtype)             `Merriwthr-OsF' (encoding: T1).
(microtype)             For optimal results, create family-specific
settings.
(microtype)             See the microtype manual for details.
LaTeX Font Info:    Redeclaring symbol font `operators' on input line 47.
LaTeX Font Info:    Encoding `OT1' has changed to `T1' for symbol font
(Font)              `operators' in the math version `normal' on input
line 47.
```

```
LaTeX Font Info:    Overwriting symbol font `operators' in version
`normal'
(Font)                  OT1/cmr/m/n --> T1/Merriwthr-OsF/m/up on input
line 47.

LaTeX Font Info:    Encoding `OT1' has changed to `T1' for symbol font
(Font)              `operators' in the math version `bold' on input line
47.
LaTeX Font Info:    Overwriting symbol font `operators' in version `bold'
(Font)                  OT1/cmr/bx/n --> T1/Merriwthr-OsF/m/up on input
line 47
.
LaTeX Font Info:    Overwriting symbol font `operators' in version `bold'
(Font)                  T1/Merriwthr-OsF/m/up --> T1/Merriwthr-OsF/b/up
on inpu
t line 47.
LaTeX Font Info:    Redeclaring math alphabet \mathbf on input line 47.
LaTeX Font Info:    Overwriting math alphabet `\mathbf' in version
`normal'
(Font)                  OT1/cmr/bx/n --> T1/Merriwthr-OsF/b/up on input
line 47
.
LaTeX Font Info:    Overwriting math alphabet `\mathbf' in version `bold'
(Font)                  OT1/cmr/bx/n --> T1/Merriwthr-OsF/b/up on input
line 47
.
LaTeX Font Info:    Redeclaring math alphabet \mathsf on input line 47.
LaTeX Font Info:    Overwriting math alphabet `\mathsf' in version
`normal'
(Font)                  OT1/cmss/m/n --> T1/MerriwthrSans-OsF/m/up on
input lin
e 47.
LaTeX Font Info:    Overwriting math alphabet `\mathsf' in version `bold'
(Font)                  OT1/cmss/bx/n --> T1/MerriwthrSans-OsF/m/up on
input li
ne 47.
LaTeX Font Info:    Redeclaring math alphabet \mathit on input line 47.
LaTeX Font Info:    Overwriting math alphabet `\mathit' in version
`normal'
(Font)                  OT1/cmr/m/it --> T1/Merriwthr-OsF/m/it on input
line 47
.
LaTeX Font Info:    Overwriting math alphabet `\mathit' in version `bold'
(Font)                  OT1/cmr/bx/it --> T1/Merriwthr-OsF/m/it on input
line 4
7.
LaTeX Font Info:    Redeclaring math alphabet \mathtt on input line 47.
LaTeX Font Info:    Overwriting math alphabet `\mathtt' in version
`normal'
(Font)                  OT1/cmtt/m/n --> T1/lmtt/m/up on input line 47.
LaTeX Font Info:    Overwriting math alphabet `\mathtt' in version `bold'
(Font)                  OT1/cmtt/m/n --> T1/lmtt/m/up on input line 47.
LaTeX Font Info:    Overwriting math alphabet `\mathsf' in version `bold'
```

```
(Font)                  T1/MerriwthrSans-OsF/m/up --> T1/MerriwthrSans-
OsF/b/up
 on input line 47.
LaTeX Font Info:    Overwriting math alphabet `\mathit' in version `bold'
(Font)                  T1/Merriwthr-OsF/m/it --> T1/Merriwthr-OsF/b/it
on inpu
t line 47.
\c@mv@tabular=\count358
\c@mv@boldtabular=\count359
(c:/texlive/2024/texmf-dist/tex/context/base/mkii/supp-pdf.mkii
[Loading MPS to PDF converter (version 2006.09.02).]
\scratchcounter=\count360
\scratchdimen=\dimen320
\scratchbox=\box87
\nofMPsegments=\count361
\nofMParguments=\count362
\everyMPshowfont=\toks52
\MPscratchCnt=\count363
\MPscratchDim=\dimen321
\MPnumerator=\count364
\makeMPintoPDFobject=\count365
\everyMPtoPDFconversion=\toks53
) (c:/texlive/2024/texmf-dist/tex/latex/epstopdf-pkg/epstopdf-base.sty
Package: epstopdf-base 2020-01-24 v2.11 Base part for package epstopdf
Package epstopdf-base Info: Redefining graphics rule for `.eps' on input
line 4
85.
(c:/texlive/2024/texmf-dist/tex/latex/latexconfig/epstopdf-sys.cfg
File: epstopdf-sys.cfg 2010/07/13 v1.3 Configuration of (r)epstopdf for
TeX Liv
e
))
*geometry* driver: auto-detecting
*geometry* detected driver: pdftex
*geometry* verbose mode - [ preamble ] result:
* driver: pdftex
* paper: a4paper
* layout: <same size as paper>
* layoutoffset:(h,v)=(0.0pt,0.0pt)
* modes: includefoot twoside
* h-part:(L,W,R)=(54.64pt, 488.22787pt, 54.64pt)
* v-part:(T,H,B)=(66.0pt, 745.04684pt, 34.0pt)
* \paperwidth=597.50787pt
* \paperheight=845.04684pt
* \textwidth=488.22787pt
* \textheight=715.04684pt
* \oddsidemargin=-17.62999pt
* \evensidemargin=-17.62999pt
* \topmargin=-47.76999pt
* \headheight=17.5pt
* \headsep=24.0pt
* \topskip=10.0pt
* \footskip=30.0pt
* \marginparwidth=48.0pt
```

```
* \marginparsep=10.0pt
* \columnsep=18.0pt
* \skip\footins=22.0pt plus 2.0pt
* \hoffset=0.0pt
* \voffset=0.0pt
* \mag=1000
* \@twocolumntrue
* \@twosidetrue
* \@mparswitchtrue
* \@reversemarginfalse
* (1in=72.27pt=25.4mm, 1cm=28.453pt)

Package caption Info: Begin \AtBeginDocument code.
Package caption Info: hyperref package is loaded.
Package caption Info: End \AtBeginDocument code.

(c:/texlive/2024/texmf-dist/tex/latex/translations/translations-basic-
dictionar
y-english.trsl
File: translations-basic-dictionary-english.trsl (english translation
file `tra
nslations-basic-dictionary')
)
Package translations Info: loading dictionary `translations-basic-
dictionary' f
or `english'. on input line 47.
Package hyperref Info: Link coloring OFF on input line 47.
(./main.out) (./main.out)
\@outlinefile=\write4
\openout4 = `main.out'.

\@gscitedetails=\box88
\@gscitedetailsheight=\skip165
\@gsheadbox=\box89
\@gsheadboxheight=\skip166
LaTeX Font Info:    Font shape `T1/Merriwthr-OsF/b/n' will be
(Font)              scaled to size 6.5pt on input line 47.
LaTeX Font Info:    Calculating math sizes for size <7.5> on input line
47.

LaTeX Font Warning: Font shape `T1/Merriwthr-OsF/m/up' undefined
(Font)              using `T1/Merriwthr-OsF/m/n' instead on input line
47.

LaTeX Font Info:    Font shape `T1/Merriwthr-OsF/m/up' will be
(Font)              scaled to size 6.24973pt on input line 47.
LaTeX Font Info:    Font shape `T1/Merriwthr-OsF/m/up' will be
(Font)              scaled to size 5.24997pt on input line 47.
LaTeX Font Info:    Trying to load font information for U+eur on input
line 47.

(c:/texlive/2024/texmf-dist/tex/latex/amsfonts/ueur.fd
File: ueur.fd 2013/01/14 v3.01 Euler Roman
) (c:/texlive/2024/texmf-dist/tex/latex/microtype/mt-eur.cfg
```

```
File: mt-eur.cfg 2006/07/31 v1.1 microtype config. file: AMS Euler Roman
(RS)
)

LaTeX Font Warning: Font shape `OMS/cmsy/m/n' in size <7.5> not available
(Font)              size <7> substituted on input line 47.

LaTeX Font Info:    External font `cmex10' loaded for size
(Font)              <7.5> on input line 47.
LaTeX Font Info:    External font `cmex10' loaded for size
(Font)              <6.24973> on input line 47.
LaTeX Font Info:    External font `cmex10' loaded for size
(Font)              <5.24997> on input line 47.
LaTeX Font Info:    Trying to load font information for U+euf on input
line 47.

(c:/texlive/2024/texmf-dist/tex/latex/amsfonts/ueuf.fd
File: ueuf.fd 2013/01/14 v3.01 Euler Fraktur
) (c:/texlive/2024/texmf-dist/tex/latex/microtype/mt-euf.cfg
File: mt-euf.cfg 2006/07/03 v1.1 microtype config. file: AMS Euler
Fraktur (RS)

)
LaTeX Font Info:    Trying to load font information for U+eus on input
line 47.

(c:/texlive/2024/texmf-dist/tex/latex/amsfonts/ueus.fd
File: ueus.fd 2013/01/14 v3.01 Euler Script
) (c:/texlive/2024/texmf-dist/tex/latex/microtype/mt-eus.cfg
File: mt-eus.cfg 2006/07/28 v1.2 microtype config. file: AMS Euler Script
(RS)
)
LaTeX Font Info:    Trying to load font information for U+euex on input
line 47
.
(c:/texlive/2024/texmf-dist/tex/latex/amsfonts/ueuex.fd
File: ueuex.fd 2013/01/14 v3.01 Euler extra symbols
)

LaTeX Font Warning: Font shape `OML/cmm/m/it' in size <7.5> not available
(Font)              size <7> substituted on input line 47.

LaTeX Font Info:    Font shape `T1/Merriwthr-OsF/m/n' will be
(Font)              scaled to size 6.24973pt on input line 47.
LaTeX Font Info:    Font shape `T1/Merriwthr-OsF/m/n' will be
(Font)              scaled to size 5.24997pt on input line 47.
LaTeX Font Info:    Font shape `T1/Merriwthr-OsF/m/it' will be
(Font)              scaled to size 7.5pt on input line 47.
LaTeX Font Info:    Font shape `T1/Merriwthr-OsF/m/it' will be
(Font)              scaled to size 6.24973pt on input line 47.
LaTeX Font Info:    Font shape `T1/Merriwthr-OsF/m/it' will be
(Font)              scaled to size 5.24997pt on input line 47.
LaTeX Font Info:    Font shape `T1/Merriwthr-OsF/m/n' will be
(Font)              scaled to size 8.0pt on input line 47.
```

```
LaTeX Font Info:    Font shape `T1/Merriwthr-OsF/m/it' will be
(Font)              scaled to size 8.0pt on input line 47.
LaTeX Font Info:    Font shape `T1/Merriwthr-OsF/b/it' will be
(Font)              scaled to size 8.0pt on input line 47.
TextBlockOrigin set to 4pc+6.64pt x 4pc+6pt
<gigascience-logo.pdf, id=44, 99.37125pt x 33.12375pt>
File: gigascience-logo.pdf Graphic file (type pdf)
<use gigascience-logo.pdf>
Package pdftex.def Info: gigascience-logo.pdf  used on input line 58.
(pdftex.def)             Requested size: 126.00902pt x 42.0pt.

Overfull \hbox (54.64pt too wide) in paragraph at lines 58--58
[][]
 []

LaTeX Font Info:    Font shape `T1/Merriwthr-OsF/m/n' will be
(Font)              scaled to size 14.0pt on input line 58.
LaTeX Font Info:    Font shape `T1/Merriwthr-OsF/m/n' will be
(Font)              scaled to size 8.99997pt on input line 58.
LaTeX Font Info:    Calculating math sizes for size <14> on input line
58.
LaTeX Font Info:    Font shape `T1/Merriwthr-OsF/m/up' will be
(Font)              scaled to size 14.0pt on input line 58.
LaTeX Font Info:    Font shape `T1/Merriwthr-OsF/m/up' will be
(Font)              scaled to size 11.66617pt on input line 58.
LaTeX Font Info:    Font shape `T1/Merriwthr-OsF/m/up' will be
(Font)              scaled to size 9.79996pt on input line 58.
LaTeX Font Info:    External font `cmex10' loaded for size
(Font)              <14> on input line 58.
LaTeX Font Info:    External font `cmex10' loaded for size
(Font)              <11.66617> on input line 58.
LaTeX Font Info:    External font `cmex10' loaded for size
(Font)              <9.79996> on input line 58.
LaTeX Font Info:    Font shape `T1/Merriwthr-OsF/m/n' will be
(Font)              scaled to size 11.66617pt on input line 58.
LaTeX Font Info:    Font shape `T1/Merriwthr-OsF/m/n' will be
(Font)              scaled to size 9.79996pt on input line 58.
LaTeX Font Info:    Font shape `T1/Merriwthr-OsF/m/it' will be
(Font)              scaled to size 14.0pt on input line 58.
LaTeX Font Info:    Font shape `T1/Merriwthr-OsF/m/it' will be
(Font)              scaled to size 11.66617pt on input line 58.
LaTeX Font Info:    Font shape `T1/Merriwthr-OsF/m/it' will be
(Font)              scaled to size 9.79996pt on input line 58.
LaTeX Font Info:    Font shape `T1/Merriwthr-OsF/b/n' will be
(Font)              scaled to size 18.0pt on input line 58.
LaTeX Font Info:    Font shape `T1/Merriwthr-OsF/m/n' will be
(Font)              scaled to size 13.0pt on input line 58.
LaTeX Font Info:    Calculating math sizes for size <13> on input line
58.
LaTeX Font Info:    Font shape `T1/Merriwthr-OsF/m/up' will be
(Font)              scaled to size 13.0pt on input line 58.
LaTeX Font Info:    Font shape `T1/Merriwthr-OsF/m/up' will be
(Font)              scaled to size 10.83287pt on input line 58.
LaTeX Font Info:    Font shape `T1/Merriwthr-OsF/m/up' will be
```

```
(Font)              scaled to size 9.09996pt on input line 58.

LaTeX Font Warning: Font shape `OMS/cmsy/m/n' in size <13> not available
(Font)              size <12> substituted on input line 58.

LaTeX Font Info:    External font `cmex10' loaded for size
(Font)              <13> on input line 58.
LaTeX Font Info:    External font `cmex10' loaded for size
(Font)              <10.83287> on input line 58.
LaTeX Font Info:    External font `cmex10' loaded for size
(Font)              <9.09996> on input line 58.

LaTeX Font Warning: Font shape `OML/cmm/m/it' in size <13> not available
(Font)              size <12> substituted on input line 58.

LaTeX Font Info:    Font shape `T1/Merriwthr-OsF/m/n' will be
(Font)              scaled to size 10.83287pt on input line 58.
LaTeX Font Info:    Font shape `T1/Merriwthr-OsF/m/n' will be
(Font)              scaled to size 9.09996pt on input line 58.
LaTeX Font Info:    Font shape `T1/Merriwthr-OsF/m/it' will be
(Font)              scaled to size 13.0pt on input line 58.
LaTeX Font Info:    Font shape `T1/Merriwthr-OsF/m/it' will be
(Font)              scaled to size 10.83287pt on input line 58.
LaTeX Font Info:    Font shape `T1/Merriwthr-OsF/m/it' will be
(Font)              scaled to size 9.09996pt on input line 58.
LaTeX Font Info:    Trying to load font information for TS1+Merriwthr-OsF
on in
put line 58.
(c:/texlive/2024/texmf-dist/tex/latex/merriweather/TS1Merriwthr-OsF.fd
File: TS1Merriwthr-OsF.fd 2020/08/30 (autoinst) Font definitions for
TS1/Merriw
thr-OsF.
)
LaTeX Font Info:    Font shape `TS1/Merriwthr-OsF/m/n' will be
(Font)              scaled to size 10.83287pt on input line 58.
Package microtype Info: Loading generic protrusion settings for font
family
(microtype)             `Merriwthr-OsF' (encoding: TS1).
(microtype)             For optimal results, create family-specific
settings.
(microtype)             See the microtype manual for details.
LaTeX Font Info:    Font shape `T1/Merriwthr-OsF/m/n' will be
(Font)              scaled to size 9.0pt on input line 58.
LaTeX Font Info:    Font shape `T1/Merriwthr-OsF/m/up' will be
(Font)              scaled to size 9.0pt on input line 58.
LaTeX Font Info:    Font shape `T1/Merriwthr-OsF/m/up' will be
(Font)              scaled to size 7.0pt on input line 58.
LaTeX Font Info:    Font shape `T1/Merriwthr-OsF/m/up' will be
(Font)              scaled to size 5.0pt on input line 58.
LaTeX Font Info:    External font `cmex10' loaded for size
(Font)              <9> on input line 58.
LaTeX Font Info:    External font `cmex10' loaded for size
(Font)              <7> on input line 58.
LaTeX Font Info:    External font `cmex10' loaded for size
```

```
(Font)              <5> on input line 58.
LaTeX Font Info:    Font shape `T1/Merriwthr-OsF/m/n' will be
(Font)              scaled to size 7.0pt on input line 58.
LaTeX Font Info:    Font shape `T1/Merriwthr-OsF/m/n' will be
(Font)              scaled to size 5.0pt on input line 58.
LaTeX Font Info:    Font shape `T1/Merriwthr-OsF/m/it' will be
(Font)              scaled to size 9.0pt on input line 58.
LaTeX Font Info:    Font shape `T1/Merriwthr-OsF/m/it' will be
(Font)              scaled to size 7.0pt on input line 58.
LaTeX Font Info:    Font shape `T1/Merriwthr-OsF/m/it' will be
(Font)              scaled to size 5.0pt on input line 58.
LaTeX Font Info:    Font shape `T1/Merriwthr-OsF/m/n' will be
(Font)              scaled to size 6.5pt on input line 58.
LaTeX Font Info:    Calculating math sizes for size <6.5> on input line
58.
LaTeX Font Info:    Font shape `T1/Merriwthr-OsF/m/up' will be
(Font)              scaled to size 6.5pt on input line 58.
LaTeX Font Info:    Font shape `T1/Merriwthr-OsF/m/up' will be
(Font)              scaled to size 5.41643pt on input line 58.
LaTeX Font Info:    Font shape `T1/Merriwthr-OsF/m/up' will be
(Font)              scaled to size 4.54997pt on input line 58.

LaTeX Font Warning: Font shape `OMS/cmsy/m/n' in size <6.5> not available
(Font)              size <6> substituted on input line 58.


LaTeX Font Warning: Font shape `OMS/cmsy/m/n' in size <5.41643> not
available
(Font)              size <5> substituted on input line 58.


LaTeX Font Warning: Font shape `OMS/cmsy/m/n' in size <4.54997> not
available
(Font)              size <5> substituted on input line 58.

LaTeX Font Info:    External font `cmex10' loaded for size
(Font)              <6.5> on input line 58.
LaTeX Font Info:    External font `cmex10' loaded for size
(Font)              <5.41643> on input line 58.
LaTeX Font Info:    External font `cmex10' loaded for size
(Font)              <4.54997> on input line 58.

LaTeX Font Warning: Font shape `OML/cmm/m/it' in size <6.5> not available
(Font)              size <6> substituted on input line 58.


LaTeX Font Warning: Font shape `OML/cmm/m/it' in size <5.41643> not
available
(Font)              size <5> substituted on input line 58.


LaTeX Font Warning: Font shape `OML/cmm/m/it' in size <4.54997> not
available
(Font)              size <5> substituted on input line 58.
```

```
LaTeX Font Info:    Font shape `T1/Merriwthr-OsF/m/n' will be
(Font)              scaled to size 5.41643pt on input line 58.
LaTeX Font Info:    Font shape `T1/Merriwthr-OsF/m/n' will be
(Font)              scaled to size 4.54997pt on input line 58.
LaTeX Font Info:    Font shape `T1/Merriwthr-OsF/m/it' will be
(Font)              scaled to size 6.5pt on input line 58.
LaTeX Font Info:    Font shape `T1/Merriwthr-OsF/m/it' will be
(Font)              scaled to size 5.41643pt on input line 58.
LaTeX Font Info:    Font shape `T1/Merriwthr-OsF/m/it' will be
(Font)              scaled to size 4.54997pt on input line 58.
LaTeX Font Info:    Font shape `TS1/Merriwthr-OsF/m/n' will be
(Font)              scaled to size 5.41643pt on input line 58.

Overfull \hbox (54.64pt too wide) in paragraph at lines 58--58
[][][]
 []

LaTeX Font Info:    Font shape `T1/Merriwthr-OsF/b/n' will be
(Font)              scaled to size 10.0pt on input line 58.
LaTeX Font Info:    Font shape `T1/Merriwthr-OsF/b/n' will be
(Font)              scaled to size 8.0pt on input line 58.

Overfull \hbox (54.64pt too wide) in paragraph at lines 58--58
[][][]
 []

LaTeX Font Info:    Font shape `T1/Merriwthr-OsF/b/n' will be
(Font)              scaled to size 7.5pt on input line 61.

Package natbib Warning: Citation `Trapnell2014' on page 1 undefined on
input li
ne 61.


Package natbib Warning: Citation `Farrell2018' on page 1 undefined on
input lin
e 61.


Package natbib Warning: Citation `Schiebinger2019' on page 1 undefined on
input
 line 61.


Package natbib Warning: Citation `LaManno2021' on page 1 undefined on
input lin
e 61.


Package natbib Warning: Citation `LaManno2018' on page 1 undefined on
input lin
e 61.
```

Package natbib Warning: Citation `Bergen2020' on page 1 undefined on
input line
 61.

Package natbib Warning: Citation `Gao2022' on page 1 undefined on input
line 61
.

Package natbib Warning: Citation `Lange2022' on page 1 undefined on input
line
61.

Package natbib Warning: Citation `Qiu2022' on page 1 undefined on input
line 61
.

Package natbib Warning: Citation `Li2023' on page 1 undefined on input
line 61.

Package natbib Warning: Citation `Cui2024' on page 1 undefined on input
line 61
.

Package natbib Warning: Citation `Gayoso2024' on page 1 undefined on
input line
 61.

Package natbib Warning: Citation `LiJ2024' on page 1 undefined on input
line 61
.

Package natbib Warning: Citation `LiS2024' on page 1 undefined on input
line 61
.

Package natbib Warning: Citation `Peng2024' on page 1 undefined on input
line 6
1.

Package natbib Warning: Citation `wang2024phylovelo' on page 1 undefined
on inp
ut line 61.

```
Package natbib Warning: Citation `ge2025tivelo' on page 1 undefined on
input li
ne 61.


Package natbib Warning: Citation `aivazidis2025cell2fate' on page 1
undefined o
n input line 61.


Package natbib Warning: Citation `wang2024regvelo' on page 1 undefined on
input
 line 61.


Package natbib Warning: Citation `chen2025graphvelo' on page 1 undefined
on inp
ut line 61.


Package natbib Warning: Citation `li2024tsvelo' on page 1 undefined on
input li
ne 61.


Package natbib Warning: Citation `wolf2019paga' on page 1 undefined on
input li
ne 61.


Package natbib Warning: Citation `schwabe2020transcriptome' on page 1
undefined
 on input line 61.


Package natbib Warning: Citation `weng2021vetra' on page 1 undefined on
input l
ine 61.


Package natbib Warning: Citation `zhang2021inference' on page 1 undefined
on in
put line 61.


Package natbib Warning: Citation `gupta2022simulation' on page 1
undefined on i
nput line 61.


Package natbib Warning: Citation `lange2022cellrank' on page 1 undefined
on inp
```

```
ut line 61.


Package natbib Warning: Citation `weiler2024cellrank' on page 1 undefined on in
put line 61.


Package natbib Warning: Citation `atta2022veloviz' on page 1 undefined on input
 line 61.


Package natbib Warning: Citation `xia2024statistical' on page 1 undefined on in
put line 61.


Package natbib Warning: Citation `sun2023dynamic' on page 1 undefined on input
line 61.


Package natbib Warning: Citation `rutkowski2025ocelli' on page 1 undefined on i
nput line 61.


Package natbib Warning: Citation `cao2018joint' on page 1 undefined on input li
ne 62.


Package natbib Warning: Citation `zhu2019ultra' on page 1 undefined on input li
ne 62.


Package natbib Warning: Citation `ma2020chromatin' on page 1 undefined on input
 line 62.


Package natbib Warning: Citation `chen2019high' on page 1 undefined on input li
ne 62.


Package natbib Warning: Citation `hunt2022sctem' on page 1 undefined on input l
ine 62.
```

Package natbib Warning: Citation `zhu2021joint' on page 1 undefined on input li
ne 62.

Package natbib Warning: Citation `pan2022scpcor' on page 1 undefined on input l
ine 62.

Package natbib Warning: Citation `tedesco2022chromatin' on page 1 undefined on
input line 62.

Package natbib Warning: Citation `bartosovic2021single' on page 1 undefined on
input line 62.

Package natbib Warning: Citation `gopalan2021simultaneous' on page 1 undefined
on input line 62.

Package natbib Warning: Citation `stuart2022nanobody' on page 1 undefined on in
put line 62.

Package natbib Warning: Citation `bartosovic2022multimodal' on page 1 undefined
 on input line 62.

Package natbib Warning: Citation `yeung2023scchix' on page 1 undefined on input
 line 62.

Package natbib Warning: Citation `stoeckius2017simultaneous' on page 1 undefine
d on input line 62.

Package natbib Warning: Citation `mimitou2021scalable' on page 1 undefined on i
nput line 62.

Package natbib Warning: Citation `Li2023' on page 1 undefined on input line 62.

```
Underfull \vbox (badness 10000) has occurred while \output is active []



Underfull \vbox (badness 10000) has occurred while \output is active []



LaTeX Font Info:    Font shape `T1/Merriwthr-OsF/m/n' will be
(Font)              scaled to size 7.8pt on input line 63.
LaTeX Font Info:    Font shape `T1/Merriwthr-OsF/b/n' will be
(Font)              scaled to size 7.8pt on input line 63.
[1{c:/texlive/2024/texmf-
var/fonts/map/pdftex/updmap/pdftex.map}{c:/texlive/202
4/texmf-
dist/fonts/enc/dvips/merriweather/merriwthr_posqbl.enc}{c:/texlive/2024
/texmf-dist/fonts/enc/dvips/merriweather/merriwthr_owzwzj.enc}


 <./gigascience-logo.pdf>]

Package natbib Warning: Citation `wang2023cannot' on page 2 undefined on
input
line 64.


Package natbib Warning: Citation `rutkowski2025ocelli' on page 2
undefined on i
nput line 64.

<figure1.png, id=66, 1468.0446pt x 1158.729pt>
File: figure1.png Graphic file (type png)
<use figure1.png>
Package pdftex.def Info: figure1.png  used on input line 70.
(pdftex.def)             Requested size: 488.22787pt x 385.35213pt.
LaTeX Font Info:    Font shape `T1/Merriwthr-OsF/m/n' will be
(Font)              scaled to size 6.0pt on input line 71.
LaTeX Font Info:    Font shape `T1/Merriwthr-OsF/b/n' will be
(Font)              scaled to size 6.0pt on input line 71.

Package natbib Warning: Citation `Bastidas-Ponce2019' on page 2 undefined
on in
put line 75.


Package natbib Warning: Citation `stuart2019comprehensive' on page 2
undefined
on input line 75.


Package natbib Warning: Citation `Bergen2020' on page 2 undefined on
input line
```

```
 75.


Package natbib Warning: Citation `wolf2018scanpy' on page 2 undefined on
input
line 75.


Package natbib Warning: Citation `stoeckius2017simultaneous' on page 2
undefine
d on input line 75.


Package natbib Warning: Citation `Gao2022' on page 2 undefined on input
line 75
.




Package natbib Warning: Citation `weinreb2020' on page 2 undefined on
input lin
e 77.

LaTeX Font Info:    Font shape `T1/Merriwthr-OsF/b/n' will be
(Font)              scaled to size 7.0pt on input line 81.
<figure2.png, id=71, 1472.8626pt x 886.512pt>
File: figure2.png Graphic file (type png)
<use figure2.png>
Package pdftex.def Info: figure2.png  used on input line 115.
(pdftex.def)             Requested size: 488.22787pt x 293.86198pt.

Package natbib Warning: Citation `Bastidas-Ponce2019' on page 2 undefined
on in
put line 116.


Package natbib Warning: Citation `Hao2021' on page 2 undefined on input
line 11
6.


Package natbib Warning: Citation `wolf2018scanpy' on page 2 undefined on
input
line 120.


Package natbib Warning: Citation `bredikhin2022muon' on page 2 undefined
on inp
ut line 120.


Package natbib Warning: Citation `scvae' on page 2 undefined on input
line 120.
```

```
Package natbib Warning: Citation `scbean' on page 2 undefined on input
line 120
.


Package natbib Warning: Citation `mcinnes2018umap' on page 2 undefined on
input
 line 122.


Package natbib Warning: Citation `jacomy2014forceatlas2' on page 2
undefined on
 input line 122.


Package natbib Warning: Citation `ding2021deep' on page 2 undefined on
input li
ne 122.



LaTeX Font Info:    Font shape `T1/Merriwthr-OsF/m/it' will be
(Font)              scaled to size 7.8pt on input line 124.
[2]
Underfull \vbox (badness 10000) has occurred while \output is active []




Underfull \vbox (badness 10000) has occurred while \output is active []



[3 <./figure1.png>]

Package natbib Warning: Citation `ASAP' on page 4 undefined on input line
128.


Package natbib Warning: Citation `cellxgene' on page 4 undefined on input
line
128.


Package natbib Warning: Citation `corvo' on page 4 undefined on input
line 128.



Package natbib Warning: Citation `SCope' on page 4 undefined on input
line 128.
```

```
Package natbib Warning: Citation `scSVA' on page 4 undefined on input
line 128.



Package natbib Warning: Citation `singlecellVR' on page 4 undefined on
input li
ne 128.


Package natbib Warning: Citation `StarmapVis' on page 4 undefined on
input line
 128.


Package natbib Warning: Citation `UCSCCellBrowser' on page 4 undefined on
input
 line 128.


Package natbib Warning: Citation `Vitessce' on page 4 undefined on input
line 1
28.


Underfull \vbox (badness 10000) has occurred while \output is active []




Underfull \vbox (badness 10000) has occurred while \output is active []



[4 <./figure2.png>]
<figure3.png, id=96, 782.925pt x 905.784pt>
File: figure3.png Graphic file (type png)
<use figure3.png>
Package pdftex.def Info: figure3.png  used on input line 132.
(pdftex.def)             Requested size: 229.46768pt x 265.47632pt.
LaTeX Font Info:    Font shape `T1/Merriwthr-OsF/m/it' will be
(Font)              scaled to size 6.0pt on input line 133.

Package natbib Warning: Citation `weinreb2020' on page 5 undefined on
input lin
e 133.


Package natbib Warning: Citation `collombat2003opposing' on page 5
undefined on
 input line 137.
```

```
Package natbib Warning: Citation `yu2021sequential' on page 5 undefined on inpu
t line 137.


Underfull \vbox (badness 10000) has occurred while \output is active []



Package natbib Warning: Citation `weinreb2020' on page 5 undefined on input lin
e 140.

<figure4.png, id=107, 1032.10594pt x 348.55219pt>
File: figure4.png Graphic file (type png)
<use figure4.png>
Package pdftex.def Info: figure4.png  used on input line 146.
(pdftex.def)             Requested size: 463.81499pt x 156.63428pt.

Package natbib Warning: Citation `Waskom2021seaborn' on page 5 undefined on inp
ut line 147.


Package natbib Warning: Citation `Hao2021' on page 5 undefined on input line 14
7.


Package natbib Warning: Citation `Bastidas-Ponce2019' on page 5 undefined on in
put line 147.


Package natbib Warning: Citation `ma2020chromatin' on page 5 undefined on input
 line 147.



[5 <./figure3.png>]

Package natbib Warning: Citation `wang2025paradigms' on page 6 undefined on inp
ut line 154.


Package natbib Warning: Citation `weinreb2020' on page 6 undefined on input lin
e 154.
```

```
Package natbib Warning: Citation `gutierrez10multifunctional' on page 6
undefin
ed on input line 154.


Package natbib Warning: Citation `mckenna2016whole' on page 6 undefined
on inpu
t line 154.


Package natbib Warning: Citation `Schiebinger2019' on page 6 undefined on
input
 line 154.


Package natbib Warning: Citation `klein2025mapping' on page 6 undefined
on inpu
t line 154.


Package natbib Warning: Citation `schott2024open' on page 6 undefined on
input
line 154.


Package natbib Warning: Citation `abdelaal2024sirv' on page 6 undefined
on inpu
t line 154.


Package natbib Warning: Citation `zhou2024spatial' on page 6 undefined on
input
 line 154.


Package natbib Warning: Citation `gu2025topological' on page 6 undefined
on inp
ut line 154.


Underfull \vbox (badness 3965) has occurred while \output is active []



LaTeX Font Info:    Font shape `TS1/Merriwthr-OsF/m/n' will be
(Font)              scaled to size 7.5pt on input line 158.
LaTeX Font Info:    Trying to load font information for T1+lmtt on input
line 1
59.
(c:/texlive/2024/texmf-dist/tex/latex/lm/t1lmtt.fd
File: t1lmtt.fd 2015/05/01 v1.6.1 Font defs for Latin Modern
)
```

```
Package microtype Info: Loading generic protrusion settings for font
family
(microtype)                `lmtt' (encoding: T1).
(microtype)                For optimal results, create family-specific
settings.
(microtype)                See the microtype manual for details.
LaTeX Font Info:    Font shape `T1/Merriwthr-OsF/m/up' will be
(Font)              scaled to size 7.5pt on input line 159.

Underfull \hbox (badness 10000) in paragraph at lines 159--160
[]\T1/Merriwthr-OsF/m/up/7.5 (+20) Project home-page: []$\T1/lmtt/m/n/7.5
https
 : / / github . com / TabakaLab /
 []


Package natbib Warning: Citation `CJgithub' on page 6 undefined on input
line 1
77.


Package natbib Warning: Citation `CJdataset' on page 6 undefined on input
line
177.

No file main.bbl.

Package natbib Warning: There were undefined citations.



[6{c:/texlive/2024/texmf-dist/fonts/enc/dvips/lm/lm-ec.enc}
<./figure4.png>]
enddocument/afterlastpage: lastpage setting LastPage.
(./main.aux)
 ***********
LaTeX2e <2024-06-01> patch level 2
L3 programming layer <2020/03/25>
 ***********

LaTeX Font Warning: Size substitutions with differences
(Font)              up to 1.0pt have occurred.


LaTeX Font Warning: Some font shapes were not available, defaults
substituted.


LaTeX Warning: Label(s) may have changed. Rerun to get cross-references
right.

Package rerunfilecheck Info: File `main.out' has not changed.
(rerunfilecheck)             Checksum:
31B3724746FAF30333E296EF0788DECC;1341.
```

```
 )
Here is how much of TeX's memory you used:
 34430 strings out of 473583
 699707 string characters out of 5732343
 2001908 words of memory out of 5000000
 56168 multiletter control sequences out of 15000+600000
 1776932 words of font info for 498 fonts, out of 8000000 for 9000
 1141 hyphenation exceptions out of 8191
 123i,12n,131p,2315b,946s stack positions out of
10000i,1000n,20000p,200000b,200000s

pdfTeX warning (dest): name{page.7} has been referenced but does not
exist, rep
laced by a fixed one

<c:/texlive/2024/texmf-dist/fonts/type1/sorkin/merriweather/Merriwthr-
Bold.pfb>
<c:/texlive/2024/texmf-dist/fonts/type1/sorkin/merriweather/Merriwthr-
BoldItali
c.pfb><c:/texlive/2024/texmf-
dist/fonts/type1/sorkin/merriweather/Merriwthr-Ita
lic.pfb><c:/texlive/2024/texmf-
dist/fonts/type1/sorkin/merriweather/Merriwthr-R
egular.pfb><c:/texlive/2024/texmf-dist/fonts/type1/public/lm/lmtt8.pfb>
Output written on main.pdf (6 pages, 5086571 bytes).
PDF statistics:
 159 PDF objects out of 1000 (max. 8388607)
 128 compressed objects within 2 object streams
 23 named destinations out of 1000 (max. 500000)
 186990 words of extra memory for PDF output out of 221844 (max.
10000000)
```

Manuscript

Click here to access/download;Manuscript;Cell_Journey_GigaScience.pdf

Placeholder for OUP logo oup.pdf

GigaScience, 2026, 1–9

doi: xx.xxxx/xxxx
Manuscript in Preparation
Technical note

TECHNICAL NOTE

Interactive analysis of single-cell trajectories in 3D space with Cell Journey

Damian Panas[1,2] and Marcin Tabaka[1, 2, *]

[1]International Centre for Translational Eye Research, Skierniewicka 10A, Warsaw, 01-230, Poland and [2]Institute of Physical Chemistry, Polish Academy of Sciences, Kasprzaka 44/52, Warsaw, 01-224, Poland

*Corresponding author: mtabaka@ichf.edu.pl

Abstract

The integration of high-throughput single-cell profiling technologies with RNA velocity analysis has enabled the reconstruction of dynamic cellular differentiation trajectories at unprecedented resolution. Despite these advances, current visualization techniques for RNA velocity are predominantly confined to two-dimensional representations, typically employing arrows or streamlines. While effective for depicting simple cellular trajectories, these approaches are insufficient for capturing the complex topologies of multipartite cellular transitions. This limitation highlights the need for advanced three-dimensional visualization tools that can more accurately convey the structure and dynamics of velocity-inferred transitions in single-cell data. Here, we present Cell Journey, an interactive visualization platform specifically developed for three-dimensional analysis and representation of RNA velocity trajectories derived from single-cell datasets. The platform features an intuitive graphical interface supporting both unimodal and multimodal data, accommodates multiple input formats, and provides extensive customization capabilities for trajectory visualization. Cell Journey computes RNA velocity vector fields on a user-defined three-dimensional grid and constructs velocity trajectories using either Euler integration or the fourth-order Runge-Kutta method. The platform enables dynamic exploration of cellular dynamics through interactive visual elements, including streamlines, streamlets, cones, and volumetric plots. Furthermore, it allows users to investigate changes in feature activity along selected paths, facilitating deeper insights into cellular state transitions within complex multimodal single-cell datasets.

Key words: Single-cell multiomics; Developmental trajectory; RNA velocity

Introduction

Single-cell RNA sequencing technologies have enabled the study of cellular differentiation trajectories at unprecedented resolution [1–4]. These methods capture static snapshots of cellular transcriptomic states, with dynamic transitions between states subsequently inferred through computational analysis. Notably, RNA velocity–based approaches have emerged as state-of-the-art tools for uncovering the directionality of cellular transitions [5–21]. In single-cell RNA sequencing data, newly transcribed (unspliced) transcripts retaining intronic sequences can be reliably distinguished from fully spliced, mature transcripts. RNA velocity analysis exploits the quantitative relationship between nascent and mature RNA molecules. By aggregating gene-specific transcriptional dynamics across the transcriptome, RNA velocity enables the prediction of future transcriptional states, effectively forecasting a cell's trajectory in gene expression space over short time scales. RNA velocity has emerged as one of the most influential frameworks for inferring cellular differentiation pathways, disentangling subpopulation kinetics, elucidating lineage relationships, and visualizing dynamic developmental processes. Its introduction has spurred the development of numerous computational methods for trajectory inference [22–28], as well as advanced visualization algorithms that incorporate RNA velocity information to represent cellular dynamics [29–32]. In recent years, we have witnessed the development of multimodal single-cell sequencing technologies that co-profile from the same cell various combinations of genome-wide feature such as transcriptome, chromatin accessibility, histone modifications, and protein epitopes [33–47]. These methods offer an opportunity to understand the temporal relation-

Compiled on: February 26, 2026.
Draft manuscript prepared by the author.

ship between different layers of gene expression regulation and increase the potential of determining cell states. Incorporation of chromatin states switch times to RNA velocity framework improves the accuracy of cell fate prediction compared to velocity estimates from RNA only [10].

The crucial analytical task in RNA velocity analysis is the calculation of cell's transition probability in high-dimensional space and its subsequent projection onto a low-dimensional embedding. The transitions between the cellular states are often visualized as arrows or streamlines on a two-dimensional (2D) cell embedding. However, 2D cell embeddings can result in significant topological misrepresentations [48]. In the context of complex developmental single-cell data, such 2D representations are often insufficient to capture the intricate topologies of multipartite continuous cellular transitions [32]. Cell Journey addresses these limitations by computing and interactively visualizing single-cell velocity-based trajectories in three-dimensional (3D) space (Fig. 1A). Cell Journey computes RNA velocity vectors on a user-defined grid to capture spatially resolved transcriptional dynamics. It then constructs 3D field lines by numerically integrating these vectors, offering a choice between the Euler method and the fourth-order Runge-Kutta algorithm. The Euler method provides a straightforward, stepwise approximation of the cell trajectory, while the fourth-order Runge-Kutta approach delivers enhanced accuracy by accounting for intermediate evaluations within each integration step. This dual-method framework allows researchers to balance computational efficiency and precision when modeling the complex dynamics of cellular state transitions.

Cell Journey is engineered to serve single-cell researchers, irrespective of their computational proficiency, by providing a platform that simplifies the exploration of single-cell datasets in 3D. Its intuitive and accessible graphical user interface (GUI) promotes seamless interaction and efficient data analysis (Fig. 1B). The software employs a state-of-the-art visualization platform to render computed 3D trajectories using graphical aids such as streamlines, streamlets, and cones (Fig. 2A). Additionally, interactive 3D scatterplots enable the dynamic computation and visualization of differentiation trajectories, either initiated from a selected cell or generated across a user-defined regular grid to mitigate the single-cell data sparsity. This functionality enables researchers to interactively explore complex trajectories and efficiently assess multimodal feature activity changes along selected trajectories (Fig. 2B).

Materials and Methods

To demonstrate its utility, Cell Journey was applied to visualize RNA velocity-based inferred trajectories from two representative datasets: a unimodal scRNA-seq dataset of mouse pancreatic endocrinogenesis [49] (GSE132188, Fig. 2A), and a multimodal CITE-Seq dataset of human bone marrow mononuclear cell (BMMC) progenitors [50] (GSE128639, Fig. 2B). Unimodal scRNA-seq pacreatic endocrinogenesis was processed with scVelo 0.2.5 [6]. The package was also used to preprocess the data and estimate RNA velocity components. Preprocessing consisted of applying filter_and_normalize function with min_shared_counts parameter equal to 20, and n_top_genes equal to 2000. Next, the moment function was applied with n_pcs and n_neighbors parameters both equal to 30. The UMAP embedding was calculated using Scanpy 1.9.6 [51] with n_components parameter equal to 3. Finally, cell velocities were projected into the UMAP using scVelo's velocity_embedding function. CITE-seq multimodal human bone marrow data was preprocessed with CITE-seq-Count v1.4.5 [46]. The obtained RNA count matrix was preprocessed with Scanpy. The following functions were applied: filter_cells with min_genes equal to 100, filter_genes with min_cells equal to 3, normalize_total and log1p with default parameters, highly_variable_genes with n_top_genes set to 5000. Next, pca and neighbors function were applied with the default parameters, and the three-dimensional UMAP embedding was calculated using the umap function with n_components parameter equal to 3. Finally, RNA velocity was inferred using UnitVelo 0.2.5.2 [7] with N_TOP_GENES parameter equal to 1000 and R2_ADJUST set to False.

Furthermore, we demonstrated Cell Journey's utility for clonal data analysis using the dataset from Weinreb *et al.* [52] (GSE140802, Fig.3). Clonal gene expression data and cell annotations were retrieved from the Allon Klein's lab GitHub repository. The data were processed using a standard Scanpy pipeline: count matrix was log-transformed using log1p function. 2,000 highly variable genes were identified with highly_variable_genes function, and then principal component analysis was conducted using the pca function. Subsequently, the neighborhood graph was constructed with the neighbors function (n_neighbors = 20, and n_pcs = 30). Finally, a 3D UMAP embedding was generated using the umap function with the n_components parameters set to 3.

Results

Cell Journey is implemented in Python 3.11.7 and leverages a robust ecosystem of libraries to ensure both functionality and user-friendliness. The core dashboard is constructed using Dash, with Dash Mantine Components and Dash Bootstrap Components enhancing the interactivity and aesthetic of the user interface. Interactive visualizations are generated with Plotly, while SciPy provides essential numerical routines, including linear and radial basis interpolation, linear smoothing, and nearest-neighbor lookups. Scikit-learn is utilized for k-means clustering of computed averaged feature activity trends, and Scanpy [51] supports comprehensive processing of single-cell data. The platform also integrates MuData [54] for handling multimodal datasets, with NumPy and Pandas managing array operations and data frames, respectively. Coloraide is employed to interpolate color palettes, ensuring visual consistency throughout the analyses. Cell Journey focuses on interactive 3D visualization and exploratory analysis of cellular transitions and therefore builds on widely used Scanpy/AnnData - based workflows for standard preprocessing. We note that Cell Journey is complementary to a broad ecosystem of upstream single-cell frameworks for representation learning and denoising [55, 56], whose outputs (e.g., embeddings and processed feature matrices) can be readily visualized within Cell Journey. This cohesive integration of Python-based computational tools enables Cell Journey to deliver scalable and precise analyses of single-cell developmental data.

The platform is engineered to address key tasks in the exploration and visualization of single-cell data (Fig. 1A). It accommodates the upload of diverse dataset formats—including h5ad for single-modality, h5mu for multimodal, and comma-separated CSV dataset files—thus ensuring broad compatibility with various data sources. It enables the visualization of cells embedded in 3D space, where representations can be based on either categorical or continuous feature activity values, such as gene expression, protein epitope levels, or cell cluster annotations. The 3D embeddings of single-cell data can be generated by an arbitrary method, including UMAP [57], FLE [58], or hyperbolic ones such as scPhere [59]. Furthermore, the platform addresses challenges in visualizing low-abundance features and compensates for dropout artifacts inherent in single-cell profiling by rendering feature activity values as partially transparent isosurfaces (volumetric/volume plots) within the 3D embedding (Fig. 2-3). Furthermore, the volume plot provides a mechanism for interpolating any desired feature by means of a selection of radial basis functions (including Gaussian, linear, quadratic, or multi-quadratic). The smoothing level of the resulting approximation can be accurately controlled via multiple independent parameters, thereby offering a high degree of flexibility in adjusting the level of precision and computational efficiency. These volume plots are

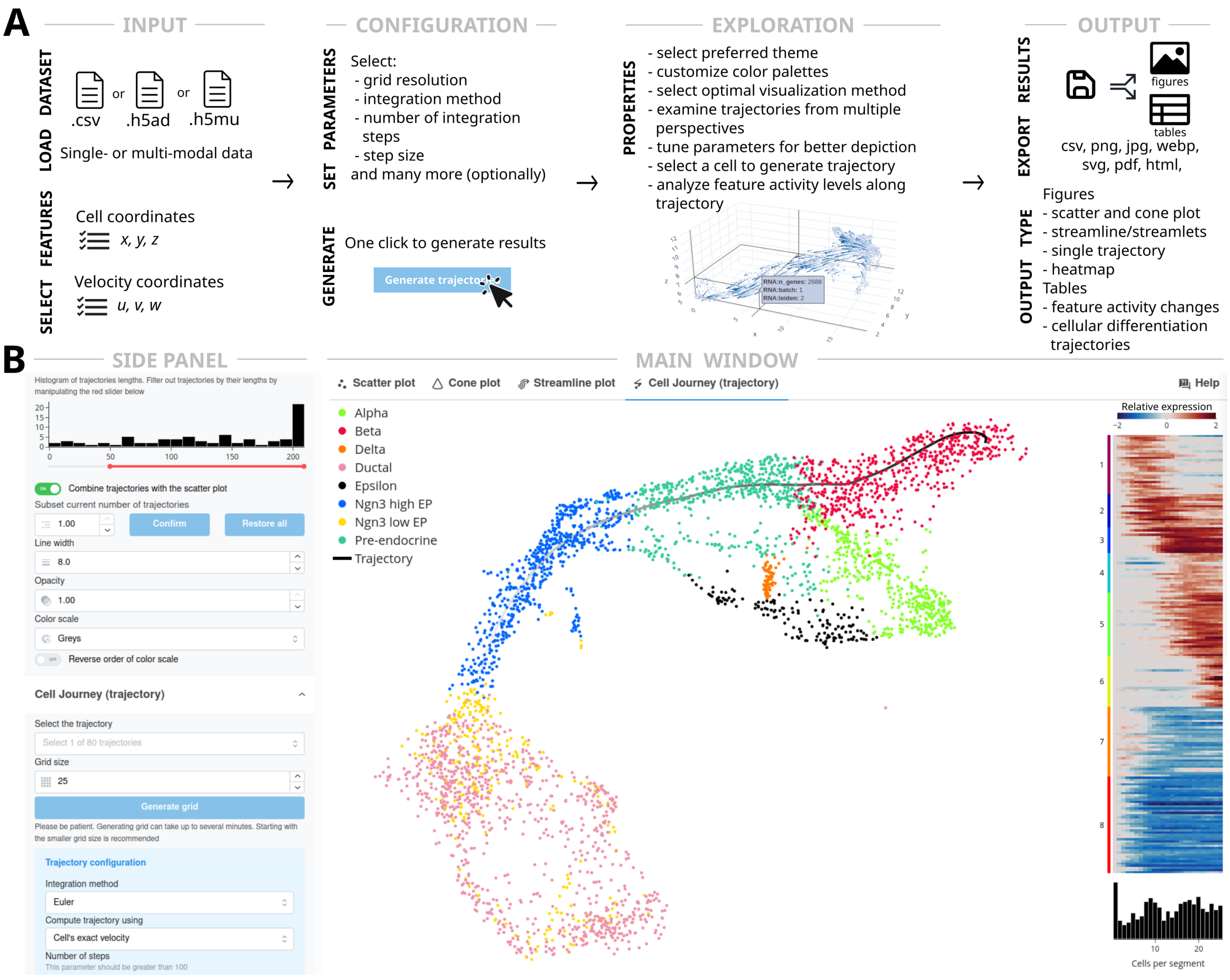

Figure 1. Cell Journey is an interactive tool for visualization and exploratory analysis of single-cell multiomics data and velocity-based trajectories. (A) Overview. Cell Journey accepts as input (left) single-cell datasets in text or hdf5 file format (h5ad or h5mu for uni- or multimodal data). Users in the first step load the data and select the variables of cell coordinates and the components of the velocity vectors. After defining the grid resolution and integration method, Cell Journey computes RNA velocity vector field lines in a 3D cell embedding. Then, the user can explore the visual depiction of the vector field, generate trajectories for a selected cell, and study the modality feature changes along the computed trajectory. Finally, generated figures can be exported in a high-resolution publication-ready format. (B), A part of the interface with a generated trajectory from a selected cell. The software has a main window and a drop-down panel on the left. The fully customizable heatmap shows activities of modality features (e.g. gene expression) along the trajectory.

fully customizable, allowing users to independently adjust the grid resolution—separate from the grid used for trajectory computation—select appropriate color palettes, and define the range of feature activity values displayed. This level of customization ensures that subtle variations in feature activities are effectively visualized, thereby enhancing the interpretability of low-abundant features like expression levels of transcription factors or surface proteins. Such volume plots become especially important in large-scale 3D visualizations, where small point sizes representing cells and low detection probabilities of features can impede the visualization of activity levels in conventional scatter plots. Extensive customization options for scatterplots are provided, allowing users to define specific scales or color palettes, or to select from an array of built-in options, including those optimized for colorblind accessibility. Additionally, figures can be exported either as static images in raster and vector formats or as interactive visualizations suitable for exploration in a web browser. Finally, Cell Journey offers a flexible interface that supports dynamic zooming of cells and trajectories, with real-time updates following adjustments to visual parameters.

To facilitate the visualization of cellular transitions in three-dimensional single-cell data embeddings, Cell Journey calculates RNA velocity vectors on a regularly spaced grid with user-defined resolution. These vectors are then used to compute 3D field lines through numerical integration, employing either the Euler method or the fourth-order Runge-Kutta algorithm. Researchers can regulate both the step count and the step size of these algorithms to obtain an optimal trajectory length. In particular, implementing parameters such as the scale grid and the difference threshold enables a fine-grained balance between computational speed and the level of detail captured in the integration. Users can flexibly adjust the density of the streamlines, generate streamlets, and customize their attributes — including length, color, transparency, and color gradients depicting direction of trajectories. Users can select specific trajectories for detailed analysis. Moreover, trajectories can be dynamically generated from any selected cell or grid element within the scatterplot. The tool further quantifies changes in uni- or multimodal feature activity levels — such as gene expression profiles or epitope levels — along the selected or generated trajectory. These activity changes are clustered according to their trends and subsequently visualized in an interactive heatmap (Fig. 2B), providing a comprehensive overview of the dynamic cellular processes at genome-wide scale. The sequence of clusters is first determined by grouping the up- and down-regulated averaged profiles and then by ordering their extremal values. This approach yields well-defined

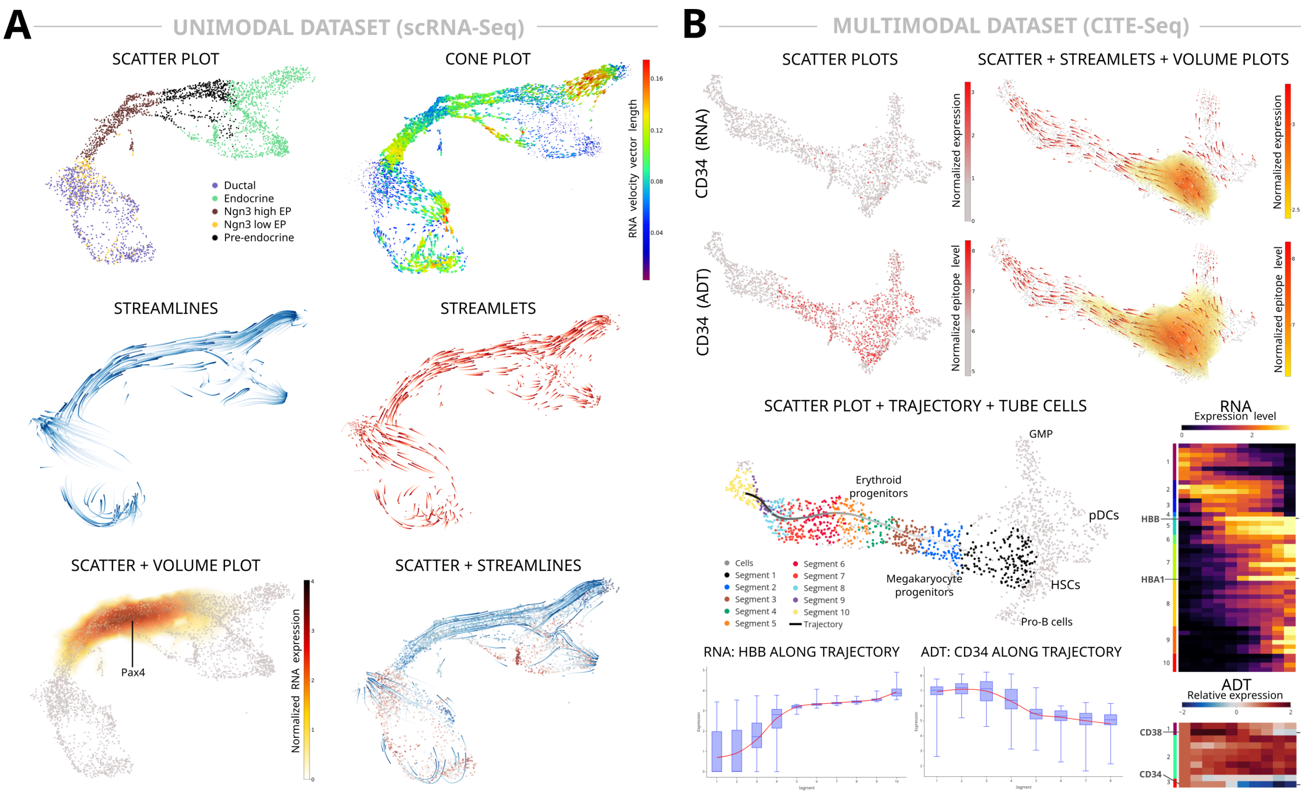

Figure 2. Cell Journey implemented visualizations of 3D single-cell embeddings and RNA velocity vector fields applied to (A) a unimodal scRNA-Seq dataset of pancreatic endocrinogenesis [49], and (B) a multimodal CITE-Seq dataset of human bone marrow mononuclear cell progenitors [53]. Cells in the neighborhood of the generated trajectory are grouped into user-specified segments to calculate the statistics of the feature activities. The interactive heatmap with grouped feature activities along the trajectory displays when clicking on a selected feature the trend of its activity in multiple formats.

temporal groupings of features that exhibit gradual transitions, enabling more precise characterization of dynamic patterns over time.

To evaluate the distinctive capabilities of Cell Journey within the landscape of interactive single-cell data analysis tools, we conducted a comprehensive comparison with a range of existing plat-

Table 1. Comparison of the functionality of Cell Journey with other platforms for interactive exploration and analysis of single-cell data.

		**Cell Journey**	ASAP	CELLxGENE	Covro	SCope	scSVA	singlecellVR	StarmapVis	UCSC Cell Browser	Vitessce
RNA Velocity	Cone plot	+									
	Volume plot	+									
	Streamlines/streamlets	+									
	Combining multiple types of plots	+									
	Trajectory from selected cell	+									
	Feature changes along the trajectory	+									
General	3D view	+	+		+		+	+	+		+
	Multimodality	+			+			+			+
	Docker	+	+	+		+	+	+			
	Example datasets	+	+	+		+		+	+	+	+
Input files	h5		+				+				
	h5ad	+		+	+		+	+		+	
	h5mu	+									
	csv/tsv/txt	+	+				+		+	+	+
	loom		+			+	+	+		+	
	Seurat/rds		+							+	
Processing	Clustering	+	+								+
	Data normalization	+	+			+					
	Scaling	+	+			+					
	Feature/cell filtering	+	+	+	+	+			+	+	

forms (Table 1), including ASAP [60], cellxgene [61], Corvo [62], SCope [63], scSVA [64], singlecellVR [65], StarmapVis [66], the UCSC Cell Browser [67], and Vitessce [68]. While several of these tools offer limited support for three-dimensional (3D) visualization of single-cell data, Cell Journey stands out as the most comprehensive platform in terms of functionality and analytical depth. It is the only tool capable of generating and rendering RNA velocity outputs directly in 3D space. Furthermore, it uniquely supports fully interactive computation, visualization, and exploration of cellular trajectories based on RNA velocity, offering an integrated platform for trajectory inference and dynamic state analysis.

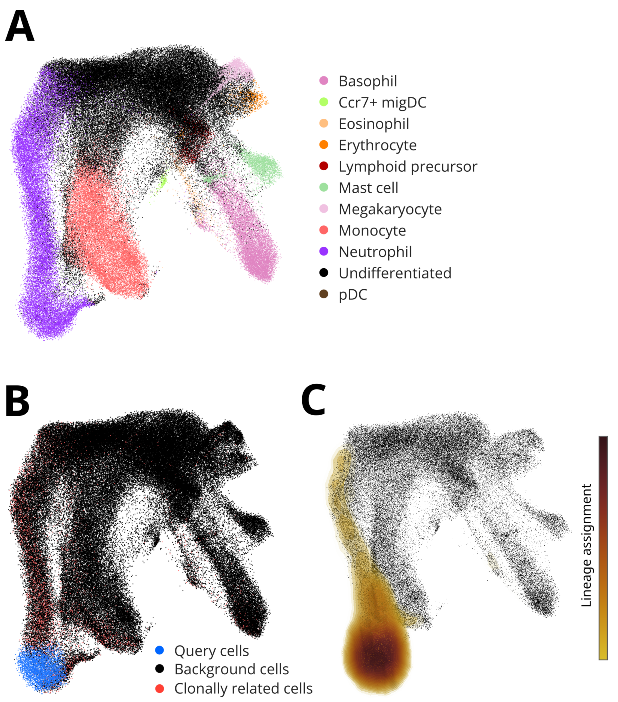

Figure 3. Example application of Cell Journey for lineage tracing analysis using clonal scRNA-seq data from Weinreb *et al.* (2020) [52]. (A) Three-dimensional UMAP visualization with cell-type annotations; (B) Identification of all cells assigned to the same clonal lineage as the cells in a user-defined region selected by a single click. To maintain full analytical control, Cell Journey highlights the query cells and their clonally related cells separately; (C) Visualization of the same data as a volume plot facilitates reconstruction of a developmental trajectory.

We next demonstrate the application of Cell Journey to single-cell transcriptomic and multi-omic datasets, illustrating its capability to resolve complex cellular dynamics through integrated 3D visualizations. Figure 2A illustrates the 3D visualization of pancreatic endocrinogenesis, where an example trajectory is generated from a selected endocrine progenitor cell progressing toward Beta cells. Gene expression dynamics along this trajectory are clustered using k-means based on their temporal trends and subsequently visualized via an interactive heatmap. The cone plot, which projects the direction of cellular transitions within the 3D embedding, is highly dependent on the precision of detecting unspliced and spliced RNA forms of key genes and consequently displays heterogeneous transition probability patterns. In contrast, streamlines and streamlets, derived from the integration of individual cell velocity vectors, yield more consistent trajectories toward fully differentiated cells. Additionally, the volume plot effectively depicts the expression level of Pax4, a key transcription factor for endocrine development [69], which is transiently expressed in endocrine progenitors [70]. For the visualization of the BMMC CITE-seq dataset in 3D (Fig. 2B), three modes are employed simultaneously: a scatter plot to display cell positions, streamlets to indicate the direction of cellular transitions, and a volume plot to portray the epitope levels of the CD34 surface marker, which is specific to hematopoietic stem cells (HSCs). Although epitope levels are readily visualized in conventional scatterplots, RNA expression is substantially sparser. Nonetheless, the volume plot derived from RNA modality data accurately reflects its HSC specificity, highlighting the utility of volume plots in delineating low-abundance features. In a subsequent visualization, the same plot orientation is used to generate a trajectory from a selected cell to erythroid progenitor cells. Segments of trajectory-projected cells are marked to compute differentially expressed feature activity levels, which are then grouped according to their trends and visualized in interactive heatmaps. Users can select the modality for analysis — such as RNA or ADT (epitope) levels — and the heatmaps can display either normalized or relative feature activity levels. Notably, the RNA heatmap highlights a pronounced upregulation of hemoglobin subunit beta (HBB) and hemoglobin alpha 1 (HBA1), components of hemoglobin A, along the erythroid lineage. Conversely, the ADT heatmap reveals a systematic decrease in CD34 protein levels from HSCs, alongside a transient upregulation of CD38 levels along the erythroid trajectory. These observations underscore the platform's robust capacity to elucidate dynamic cellular processes across multiple data modalities.

Finally, we demonstrate that Cell Journey can also be applied to clonal scRNA-seq lineage-tracing data, where "ground-truth" relationships between cells are provided by shared barcodes rather than inferred velocities. Using the DNA-barcoding dataset from Weinreb et al. (2020) [52] (Fig. 3A), Cell Journey enables interactive identification and visualization of clonally related cells directly within a 3D embedding (Fig. 3B-C). Specifically, a user can select a region of interest with a single click (Fig. 3B), after which the platform highlights the query cells and all cells assigned to the same clonal lineage (neutrophil lineage in Fig. 3B), while keeping background cells visible to preserve global context. Importantly, because barcode-based lineage tracing does not provide vector-valued directionality in transcriptional space, analysis focuses on the distribution of clone membership across cellular states. In this setting, representing clonal enrichment as a volume plot (Fig. 3C) provides a smooth, continuous depiction of clone-associated regions over the manifold, which can be difficult to interpret from sparse point overlays alone, particularly when clonally related cells are rare or dispersed. The number and spread of clonally related cells across states can provide constraints on likely state-to-fate relationships (e.g., clonal expansion and shared ancestry across compartments), and Cell Journey facilitates interactive exploration of these patterns in 3D.

To assess the computational efficiency of Cell Journey during its most demanding tasks, we performed benchmarking across various datasets, integration methods, and grid sizes (Fig. 4). Our findings indicate that both the integration method and grid resolution influence computation time; however, both vector-field averaging and trajectory integration remain efficient (Fig. 4A-B). Even at higher grid resolutions, execution times stay within a range that allows for near-instantaneous analysis without prolonged waiting periods. While we observed a non-linear increase in processing time as the grid resolution increases, the absolute duration remains low across all tested datasets. Furthermore, comparing integration strategies revealed that the Euler method is significantly more efficient than the fourth-order Runge-Kutta, offering approximately a 40% reduction in processing time (Fig. 4C). Consequently, we recommend that users begin data exploration using the default parameters and subsequently adjust them according to their available computational resources to achieve the desired balance between detail and speed.

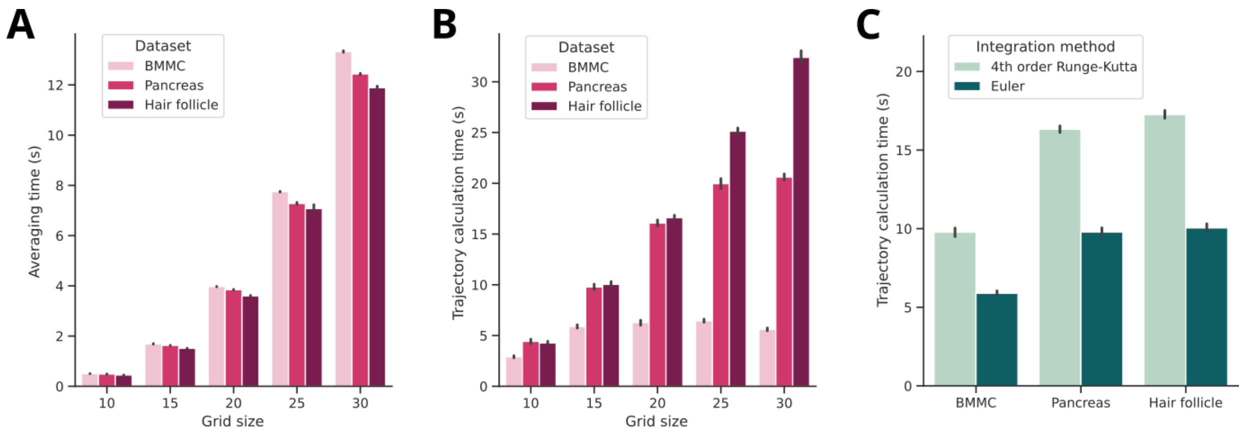

Figure 4. Computational efficiency of trajectory vector-field averaging and trajectory integration. (A) Benchmarking Euler integration across varying grid densities. (B) Comparative performance of different integration methods at a fixed grid size of 15. Bars represent the mean execution time over 10 independent runs, with whiskers denoting 95% confidence intervals (CIs). These CIs were estimated via the bootstrap method using the Seaborn Python package [71]. All benchmarks were conducted on a Dell Latitude X1201 (16 GB RAM) running the Debian 13 operating system. Datasets: BMMCs — human bone marrow mononuclear cell progenitors [53]; Pancreas — pancreatic endocrinogenesis [49]; Hair follicle — regenerative compartment of the hair follicle [35].

Discussion

Our objective is to facilitate rapid and in-depth exploration of single-cell developmental data in 3D, thereby providing immediate insights and fostering a nuanced understanding of cellular trajectories. Cell Journey is accessible to researchers regardless of computational expertise, featuring a streamlined setup process that requires only a few simple steps and an intuitive, user-friendly interface. A comprehensive help panel, accompanied by FAQs and sample datasets, enables users to quickly familiarize themselves with the tool's functionalities. Designed with extensibility in mind, Cell Journey accommodates the integration of additional analytical modules, thereby supporting increasingly comprehensive analyses. Moreover, the software is optimized for speed, efficiency, and robustness, ensuring reliable performance even when processing large-scale single-cell datasets. Cell Journey is under active development. We plan to extend its functionality by providing new modules for differential trajectory analysis and integration with workspaces for comprehensive single-cell multimodal data analysis. We actively invite user feedback and feature suggestions to drive the continuous enhancement of its functionality, and we are committed to providing regular updates and fostering active community engagement to ensure the tool's ongoing relevance and innovation.

While Cell Journey was originally developed to visualize transitions between cellular states inferred from RNA velocity analyses [72], its framework is broadly applicable to any approach, experimental or computational, that delineates cellular state transitions. In particular, Cell Journey can be used to visualize ground-truth transitions and lineage relationships derived from lineage-tracing experiments. As demonstrated in Fig. 3, Cell Journey supports interactive exploration of DNA-barcoding clonal scRNA-seq data (e.g., Weinreb *et al.* [52]; see also related DNA-barcoding approaches [73]), enabling users to highlight clonally related cells and reconstruct lineage trajectories directly on transcriptional embeddings. More generally, Cell Journey is also compatible with other lineage-tracing modalities, including Cas9-based cellular ancestry recording [74]. These experimental strategies label cells with heritable genetic markers, allowing reconstruction of clonal dynamics and lineage relationships from shared barcodes or mutation patterns, which can then be visualized and interrogated within Cell Journey. Furthermore, Cell Journey can be potentially used to integrate and visualize trajectories from transition probabilities between cells computationally inferred using optimal transport algorithms applied to time-course single-cell transcriptomic data [3] or multimodal or spatial datasets [75]. Finally, we foresee that Cell Journey will be instrumental in visualizing putative cellular transitions within 3D spatial transcriptomics datasets [76], particularly when combined with emerging methodologies for spatial RNA velocity analysis [77–79]. This versatility underscores the potential of Cell Journey to facilitate a comprehensive understanding of cellular dynamics across diverse experimental and computational platforms.

Availability of Source Code and Requirements

- Project name: Cell Journey
- Project homepage: `https://github.com/TabakaLab/CellJourney`
- Documentation: `https://tabakalab.github.io/CellJourney`
- Operating system(s): Platform independent
- Programming languages: Python
- Other requirements: Python 3.11.7, Dash 3.3.0, Plotly 6.5.2
- License: MIT license

Acknowledgements and Funding

We thank members of the Computational Genomics Group for their discussions, with particular thanks to Piotr Rutkowski for his assistance in preprocessing the human bone marrow mononuclear cell data. D.P. and M.T. are supported by the „International Centre for Translational Eye Research" project, which is carried out within the MAB FENG action 02.01. of the Foundation for Polish Science co-financed by the European Union under the European Regional Development Fund, European Funds for Smart Economy, agreement no. FENG.02.01-IP.05-T005/23.

Author's Contributions

M.T. conceived and supervised the project. M.T. and D.P. designed and developed Cell Journey. D.P. implemented Cell Journey and wrote its documentation. M.T. and D.P. wrote the manuscript.

Competing Interests

The authors declare no competing interests.

Data Availability

Cell Journey is under the MIT License. The software, documentation, tutorials, example datasets, and animated demos can be found at GitHub [80]. All additional supporting data are available in the *GigaScience* repository, GigaDB [81].

References

1. Trapnell C, Cacchiarelli D, Grimsby J, Pokharel P, Li S, Morse M, et al. The dynamics and regulators of cell fate decisions are revealed by pseudotemporal ordering of single cells. Nature Biotechnology 2014;32(4):381–386. https://doi.org/10.1038/nbt.2859.
2. Farrell JA, Wang Y, Riesenfeld SJ, Shekhar K, Regev A, Schier AF. Single-cell reconstruction of developmental trajectories during zebrafish embryogenesis. Science 2018;360(6392):eaar3131. https://doi.org/10.1126/science.aar3131.
3. Schiebinger G, Shu J, Tabaka M, Cleary B, Subramanian V, Solomon A, et al. Optimal-Transport Analysis of Single-Cell Gene Expression Identifies Developmental Trajectories in Reprogramming. Cell 2019;176(4):928–943.e22. https://doi.org/10.1016/j.cell.2019.01.006.
4. La Manno G, Siletti K, Furlan A, Gyllborg D, Vinsland E, Mossi Albiach A, et al. Molecular architecture of the developing mouse brain. Nature 2021;596(7870):92–96. https://doi.org/10.1038/s41586-021-03775-x.
5. La Manno G, Soldatov R, Zeisel A, Braun E, Hochgerner H, Petukhov V, et al. RNA velocity of single cells. Nature 2018;560(7719):494–498. https://doi.org/10.1038/s41586-018-0414-6.
6. Berker V, Lange M, Paidli S, Wolf FA, Theis FJ. Generalizing RNA velocity to transient cell states through dynamical modeling. Nature Biotechnology 2020;38(12):1408–1414. https://doi.org/10.1038/s41587-020-0591-3.
7. Gao M, Qiao C, Huang Y. UniTVelo: temporally unified RNA velocity reinforces single-cell trajectory inference. Nature Communications 2022;13(1):6586. https://doi.org/10.1038/s41467-022-34188-7.
8. Lange M, Bergen V, Klein M, Setty M, Reuter B, Bakhti M, et al. CellRank for directed single-cell fate mapping. Nature Methods 2022;19(2):159–170. https://doi.org/10.1038/s41592-021-01346-6.
9. Qiu X, Zhang Y, Martin-Rufino JD, Weng C, Hosseinzadeh S, Yang D, et al. Mapping transcriptomic vector fields of single cells. Cell 2022;185(4):690–711.e45. https://doi.org/10.1016/j.cell.2021.12.045.
10. Li C, Virgilio MC, Collins KL, Welch JD. Multi-omic single-cell velocity models epigenome–transcriptome interactions and improves cell fate prediction. Nature Biotechnology 2023;41(3):387–398. https://doi.org/10.1038/s41587-022-01476-y.
11. Cui H, Maan H, Vladoiu MC, Zhang J, Taylor MD, Wang B. DeepVelo: deep learning extends RNA velocity to multi-lineage systems with cell-specific kinetics. Genome Biology 2024;25(1):27. https://doi.org/10.1186/s13059-023-03148-9.
12. Gayoso A, Weiler P, Lotfollahi M, Klein D, Hong J, Streets A, et al. Deep generative modeling of transcriptional dynamics for RNA velocity analysis in single cells. Nature Methods 2024;21(1):50–59. https://doi.org/10.1038/s41592-023-01994-w.
13. Li J, Pan X, Yuan Y, Shen HB. TFvelo: gene regulation inspired RNA velocity estimation. Nature Communications 2024;15(1):1387. https://doi.org/10.1038/s41467-024-45661-w.
14. Li S, Zhang P, Chen W, Ye L, Brannan KW, Le NT, et al. A relay velocity model infers cell-dependent RNA velocity. Nature Biotechnology 2024;42(1):99–108. https://doi.org/10.1038/s41587-023-01728-5.
15. Peng Q, Qiu X, Li T. Storm: Incorporating transient stochastic dynamics to infer the RNA velocity with metabolic labeling information. PLOS Computational Biology 2024 11;20(11):1–38. https://doi.org/10.1371/journal.pcbi.1012606.
16. Wang K, Hou L, Wang X, Zhai X, Lu Z, Zi Z, et al. PhyloVelo enhances transcriptomic velocity field mapping using monotonically expressed genes. Nature Biotechnology 2024;42(5):778–789. https://doi.org/10.1038/s41587-023-01887-5.
17. Ge M, Miao J, Qi J, Zhou X, Lin Z. TIVelo: RNA velocity estimation leveraging cluster-level trajectory inference. Nature Communications 2025;16(1):6258. https://doi.org/10.1038/s41467-025-61628-x.
18. Aivazidis A, Memi F, Kleshchevnikov V, Er S, Clarke B, Stegle O, et al. Cell2fate infers RNA velocity modules to improve cell fate prediction. Nature Methods 2025;p. 1–10. https://doi.org/10.1038/s41592-025-02608-3.
19. Wang W, Hu Z, Weiler P, Mayes S, Lange M, Wang J, et al. RegVelo: gene-regulatory-informed dynamics of single cells. bioRxiv 2024;https://doi.org/10.1101/2024.12.11.627935.
20. Chen Y, Zhang Y, Gan J, Ni K, Chen M, Bahar I, et al. GraphVelo allows for accurate inference of multimodal velocities and molecular mechanisms for single cells. Nature Communications 2025;16:7831. https://doi.org/10.1038/s41467-025-62784-w.
21. Li J, Wang Z, Shen HB, Yuan Y. TSvelo: Comprehensive RNA velocity inference by jointly modeling Transcription and Splicing. bioRxiv 2024;https://doi.org/10.1101/2024.12.24.630058.
22. Wolf FA, Hamey FK, Plass M, Solana J, Dahlin JS, Göttgens B, et al. PAGA: graph abstraction reconciles clustering with trajectory inference through a topology preserving map of single cells. Genome Biology 2019;20(1):59. https://doi.org/10.1186/s13059-019-1663-x.
23. Schwabe D, Formichetti S, Junker JP, Falcke M, Rajewsky N. The transcriptome dynamics of single cells during the cell cycle. Molecular Systems Biology 2020;16(11):e9946. https://doi.org/10.15252/msb.20209946.
24. Weng G, Kim J, Won KJ. VeTra: a tool for trajectory inference based on RNA velocity. Bioinformatics 2021;37(20):3509–3513. https://doi.org/10.1093/bioinformatics/btab364.
25. Zhang Z, Zhang X. Inference of high-resolution trajectories in single-cell RNA-seq data by using RNA velocity. Cell Reports Methods 2021;1(6). https://doi.org/10.1016/j.crmeth.2021.1000954.
26. Gupta R, Cerletti D, Gut G, Oxenius A, Claassen M. Simulation-based inference of differentiation trajectories from RNA velocity fields. Cell Reports Methods 2022;2(12). https://doi.org/10.1016/j.crmeth.2022.100359.
27. Lange M, Bergen V, Klein M, Setty M, Reuter B, Bakhti M, et al. CellRank for directed single-cell fate mapping. Nature Methods 2022;19(2):159–170. https://doi.org/10.1038/s41592-021-01346-6.
28. Weiler P, Lange M, Klein M, Pe'er D, Theis F. CellRank 2: unified fate mapping in multiview single-cell data. Nature Methods 2024;21(7):1196–1205. https://doi.org/10.1038/s41592-024-02303-9.
29. Atta L, Sahoo A, Fan J. VeloViz: RNA velocity-informed embeddings for visualizing cellular trajectories. Bioinformatics 2022;38(2):391–396. https://doi.org/10.1093/bioinformatics/btab653.
30. Xia L, Lee C, Li JJ. Statistical method scDEED for detecting dubious 2D single-cell embeddings and optimizing t-SNE and UMAP hyperparameters. Nature Communications 2024;15(1):1753. https://doi.org/10.1038/s41467-024-45891-y.
31. Sun ED, Ma R, Zou J. Dynamic visualization of high-dimensional data. Nature Computational Science 2023;3(1):86–100. https://doi.org/10.1038/s43588-022-00380-4.

32. Rutkowski P, Tabaka M. Ocelli: an open-source tool for the analysis and visualization of developmental multimodal single-cell data. NAR Genomics and Bioinformatics 2025;7(2):lqaf040. https://doi.org/10.1093/nargab/lqaf040.
33. Cao J, Cusanovich DA, Ramani V, Aghamirzaie D, Pliner HA, Hill AJ, et al. Joint profiling of chromatin accessibility and gene expression in thousands of single cells. Science 2018;361(6409):1380–1385. https://doi.org/10.1126/science.aau0730.
34. Zhu C, Yu M, Huang H, Juric I, Abnousi A, Hu R, et al. An ultra high-throughput method for single-cell joint analysis of open chromatin and transcriptome. Nature Structural & Molecular Biology 2019;26(11):1063–1070. https://doi.org/10.1038/s41594-019-0323-x.
35. Ma S, Zhang B, LaFave LM, Earl AS, Chiang Z, Hu Y, et al. Chromatin potential identified by shared single-cell profiling of RNA and chromatin. Cell 2020;183(4):1103–1116. https://doi.org/10.1016/j.cell.2020.09.056.
36. Chen S, Lake BB, Zhang K. High-throughput sequencing of the transcriptome and chromatin accessibility in the same cell. Nature Biotechnology 2019;37(12):1452–1457. https://doi.org/10.1038/s41587-019-0290-0.
37. Hunt KV, Burnard SM, Roper EA, Bond DR, Dun MD, Verrills NM, et al. scTEM-seq: Single-cell analysis of transposable element methylation to link global epigenetic heterogeneity with transcriptional programs. Scientific Reports 2022;12(1):5776. https://doi.org/10.1038/s41598-022-09765-x0.
38. Zhu C, Zhang Y, Li YE, Lucero J, Behrens MM, Ren B. Joint profiling of histone modifications and transcriptome in single cells from mouse brain. Nature Methods 2021;18(3):283–292. https://doi.org/10.1038/s41592-021-01060-30.
39. Pan L, Ku WL, Tang Q, Cao Y, Zhao K. scPCOR-seq enables co-profiling of chromatin occupancy and RNAs in single cells. Communications Biology 2022;5(1):678. https://doi.org/10.1038/s42003-022-03584-6.
40. Tedesco M, Giannese F, Lazarević D, Giansanti V, Rosano D, Monzani S, et al. Chromatin Velocity reveals epigenetic dynamics by single-cell profiling of heterochromatin and euchromatin. Nature Biotechnology 2022;40(2):235–244. https://doi.org/10.1038/s41587-021-01031-1.
41. Bartosovic M, Kabbe M, Castelo-Branco G. Single-cell CUT&Tag profiles histone modifications and transcription factors in complex tissues. Nature Biotechnology 2021;39(7):825–835. https://doi.org/10.1038/s41587-021-00869-9.
42. Gopalan S, Wang Y, Harper NW, Garber M, Fazzio TG. Simultaneous profiling of multiple chromatin proteins in the same cells. Molecular Cell 2021;81(22):4736–4746. https://doi.org/10.1016/j.molcel.2021.09.019.
43. Stuart T, Hao S, Zhang B, Mekerishvili L, Landau DA, Maniatis S, et al. Nanobody-tethered transposition enables multifactorial chromatin profiling at single-cell resolution. Nature Biotechnology 2022;p. 1–7. https://doi.org/10.1038/s41587-022-01588-5.
44. Bartosovic M, Castelo-Branco G. Multimodal chromatin profiling using nanobody-based single-cell CUT&Tag. Nature Biotechnology 2022;p. 1–12. https://doi.org/10.1038/s41587-022-01535-4.
45. Yeung J, Florescu M, Zeller P, de Barbanson BA, Wellenstein MD, van Oudenaarden A. scChIX-seq infers dynamic relationships between histone modifications in single cells. Nature Biotechnology 2023;p. 1–11. https://doi.org/10.1038/s41587-022-01560-3.
46. Stoeckius M, Hafemeister C, Stephenson W, Houck-Loomis B, Chattopadhyay PK, Swerdlow H, et al. Simultaneous epitope and transcriptome measurement in single cells. Nature Methods 2017;14(9):865–868. https://doi.org/10.1038/nmeth.4380.
47. Mimitou EP, Lareau CA, Chen KY, Zorzetto-Fernandes AL, Hao Y, Takeshima Y, et al. Scalable, multimodal profiling of chromatin accessibility, gene expression and protein levels in single cells. Nature Biotechnology 2021;39(10):1246–1258. https://doi.org/10.1038/s41587-021-00927-2.
48. Wang S, Sontag ED, Lauffenburger DA. What cannot be seen correctly in 2D visualizations of single-cell 'omics data? Cell Systems 2023;14(9):723–731. https://doi.org/10.1016/j.cels.2023.07.002.
49. Bastidas-Ponce A, Tritschler S, Dony L, Scheibner K, Tarquis-Medina M, Salinno C, et al. Comprehensive single cell mRNA profiling reveals a detailed roadmap for pancreatic endocrinogenesis. Development 2019 06;146(12):dev173849. https://doi.org/10.1242/dev.173849.
50. Stuart T, Butler A, Hoffman P, Hafemeister C, Papalexi E, Mauck WM, et al. Comprehensive integration of single-cell data. Cell 2019;177(7):1888–1902. https://doi.org/10.1016/j.cell.2019.05.031.
51. Wolf FA, Angerer P, Theis FJ. SCANPY: large-scale single-cell gene expression data analysis. Genome Biology 2018;19(1):15. https://doi.org/10.1186/s13059-017-1382-0.
52. Weinreb C, Rodriguez-Fraticelli A, Camargo FD, Klein AM. Lineage tracing on transcriptional landscapes links state to fate during differentiation. Science 2020;367(6479):eaaw3381. https://doi.org/10.1126/science.aaw3381.
53. Hao Y, Hao S, Andersen-Nissen E, Mauck WMr, Zheng S, Butler A, et al. Integrated analysis of multimodal single-cell data. Cell 2021;184(13):3573–3587.e29. https://doi.org/10.1016/j.cell.2021.04.048.
54. Bredikhin D, Kats I, Stegle O. MUON: multimodal omics analysis framework. Genome Biology 2022;23(1):42. https://doi.org/10.1186/s13059-021-02577-8.
55. Grønbech CH, Vording MF, Timshel PN, Sønderby CK, Pers TH, Winther O. scVAE: variational auto-encoders for single-cell gene expression data. Bioinformatics 2020;36(16):4415–4422. https://doi.org/10.1093/bioinformatics/btaa293.
56. Zhang H, Wang Y, Lian B, Wang Y, Li X, Wang T, et al. Scbean: a python library for single-cell multi-omics data analysis. Bioinformatics 2024;p. btae053. https://doi.org/10.1093/bioinformatics/btae053.
57. McInnes L, Healy J, Melville J. Umap: Uniform manifold approximation and projection for dimension reduction. arXiv preprint arXiv:180203426 2018;https://doi.org/10.48550/arXiv.1802.03426.
58. Jacomy M, Venturini T, Heymann S, Bastian M. ForceAtlas2, a continuous graph layout algorithm for handy network visualization designed for the Gephi software. PLOS ONE 2014;9(6):e98679. https://doi.org/10.1371/journal.pone.0098679.
59. Ding J, Regev A. Deep generative model embedding of single-cell RNA-Seq profiles on hyperspheres and hyperbolic spaces. Nature Communications 2021;12(1):2554. https://doi.org/10.1038/s41467-021-22851-4.
60. Gardeux V, David FPA, Shajkofci A, Schwalie PC, Deplancke B. ASAP: a web-based platform for the analysis and interactive visualization of single-cell RNA-seq data. Bioinformatics 2017;33(19):3123–3125. https://doi.org/10.1093/bioinformatics/btx337.
61. Program CCS, Abdulla S, Aevermann B, Assis P, Badajoz S, Bell SM, et al. CZ CELLxGENE Discover: a single-cell data platform for scalable exploration, analysis and modeling of aggregated data. Nucleic Acids Research 2024 11;53(D1):D886–D900. https://doi.org/10.1093/nar/gkae1142.
62. Hyman L, Sbalzarini IF, Quake S, Günther U, Corvo: Visualizing CellxGene Single-Cell Datasets in Virtual Reality; 2022. https://doi.org/10.48550/arXiv.2212.00519.
63. Davie K, Janssens J, Koldere D, De Waegeneer M, Pech U, Kreft L, et al. A Single-Cell Transcriptome Atlas of the Aging Drosophila Brain. Cell 2018;174(4):982–998.e20. https://doi.org/10.

1016/j.cell.2018.05.057.
64. Tabaka M, Gould J, Regev A. scSVA: an interactive tool for big data visualization and exploration in single-cell omics. bioRxiv 2019;https://doi.org/10.1101/512582.
65. Stein DF, Chen H, Vinyard ME, Qin Q, Combs RD, Zhang Q, et al. singlecellVR: Interactive Visualization of Single-Cell Data in Virtual Reality. Frontiers in Genetics 2021;12. https://doi.org/10.3389/fgene.2021.764170.
66. Ma S, Fang X, Yao Y, Li J, Morgan DC, Xia Y, et al. StarmapVis: An interactive and narrative visualisation tool for single-cell and spatial data. Computational and Structural Biotechnology Journal 2023;21:1598–1605. https://doi.org/10.1016/j.csbj.2023.02.023.
67. Speir ML, Bhaduri A, Markov NS, Moreno P, Nowakowski TJ, Papatheodorou I, et al. UCSC Cell Browser: visualize your single-cell data. Bioinformatics 2021 07;37(23):4578–4580. https://doi.org/10.1093/bioinformatics/btab503.
68. Keller MS, Gold I, McCallum C, Manz T, Kharchenko PV, Gehlenborg N. Vitessce: integrative visualization of multimodal and spatially resolved single-cell data. Nature Methods 2025;22(1):63–67. https://doi.org/10.1038/s41592-024-02436-x.
69. Collombat P, Mansouri A, Hecksher-Sørensen J, Serup P, Krull J, Gradwohl G, et al. Opposing actions of Arx and Pax4 in endocrine pancreas development. Genes & Development 2003;17(20):2591–2603. https://doi.org/10.1101/gad.269003.
70. Yu XX, Qiu WL, Yang L, Wang YC, He MY, Wang D, et al. Sequential progenitor states mark the generation of pancreatic endocrine lineages in mice and humans. Cell Research 2021;31(8):886–903. https://doi.org/10.1038/s41422-021-00486-w.
71. Waskom ML. seaborn: statistical data visualization. Journal of Open Source Software 2021;6(60):3021. https://doi.org/10.21105/joss.0302.
72. Wang Y, Li J, Zha H, Liu S, Huang D, Fu L, et al. Paradigms, innovations, and biological applications of RNA velocity: a comprehensive review. Briefings in Bioinformatics 2025;26(4):bbaf339. https://doi.org/10.1093/bib/bbaf339.
73. Gutierrez C, Al'Khafaji A, Brenner E, Johnson K, Gohil S, Lin Z, et al. Multifunctional barcoding with ClonMapper enables high-resolution study of clonal dynamics during tumor evolution and treatment. Nature Cancer 2021;2(7):758–772. https://doi.org/10.1038/s43018-021-00222-8.
74. McKenna A, Findlay GM, Gagnon JA, Horwitz MS, Schier AF, Shendure J. Whole-organism lineage tracing by combinatorial and cumulative genome editing. Science 2016;353(6298):aaf7907. https://doi.org/10.1126/science.aaf7907.
75. Klein D, Palla G, Lange M, Klein M, Piran Z, Gander M, et al. Mapping cells through time and space with moscot. Nature 2025;638(8052):1065–1075. https://doi.org/10.1038/s41586-024-08453-2.
76. Schott M, León-Periñán D, Splendiani E, Strenger L, Licha JR, Pentimalli TM, et al. Open-ST: High-resolution spatial transcriptomics in 3D. Cell 2024;187(15):3953–3972. https://doi.org/10.1016/j.cell.2024.05.055.
77. Abdelaal T, Grossouw LM, Pasterkamp RJ, Lelieveldt BP, Reinders MJ, Mahfouz A. SIRV: spatial inference of RNA velocity at the single-cell resolution. NAR Genomics and Bioinformatics 2024;6(3):lqae100. https://doi.org/10.1093/nargab/lqae100.
78. Zhou P, Bocci F, Li T, Nie Q. Spatial transition tensor of single cells. Nature Methods 2024;21(6):1053–1062. https://doi.org/10.1038/s41592-024-02266-x.
79. Gu Y, Liu J, Lee KH, Li C, Lu L, Moline J, et al. Topological velocity inference from spatial transcriptomic data. Nature Biotechnology 2025;p. 1–12. https://doi.org/10.1038/s41587-025-02688-8.
80. Cell Journey website. https://TabakaLab.github.io/CellJourney; Accessed 20 December, 2025.
81. Panas D, Tabaka M. Supporting data for "Interactive analysis of single-cell trajectories in 3D space with Cell Journey". GigaScience Database; 2026, https://doi.org/10.5524/102805.

Supporting 3D embedding for Reviewer 1

Click here to access/download
Supplementary Material
Cell Journey BMMC 3D.html

Click here to access/download;Personal Cover;Cell Journey responses to the reviewers.pdf

Responses to the Reviewers' comments for manuscript GIGA-D-25-00322

We thank all the Reviewers for their comments and have updated the software, its documentation, and revised the manuscript accordingly. Please note that introducing some of the fixes and improvements required the use of additional packages or packages that had already been used, but in newer versions. For this reason, if you wish to test the updated version of Cell Journey, it is necessary to update the environment according to the requirements.txt file provided in the GitHub repository.

Reviewer #1

The manuscript introduces Cell Journey; an interactive visualization platform designed for three-dimensional (3D) analysis and representation of RNA velocity trajectories from single-cell datasets. The proposed tool appears to address an important gap in the field and could be of significant interest to the single-cell community.

Thank you for the positive reception of our work and for sharing the opinion that Cell Journey fills an important gap in the field and has the potential to be a useful tool in the single-cell community.

This is a promising manuscript that presents a potentially impactful visualization platform for single-cell RNA velocity analysis. However, to fully demonstrate the utility and adoption potential of Cell Journey, the authors should strengthen validation, provide more concrete examples of biological insights gained, and address usability and performance aspects. The manuscript would benefit from more rigorous validation of Cell Journey. For example, how do the 3D velocity trajectories compare to known biological ground truths (e.g., hematopoietic differentiation)? Are there quantitative benchmarks demonstrating that 3D visualization uncovers relationships not visible in 2D?

We agree that providing more examples would give a better overview of the differences between 2D and 3D embeddings. To keep the article concise, we focused on Cell Journey's key functionalities, as it is primarily a visualization and exploratory software, and its output relies on results from other analytical tools such as scanpy, scVelo, or UniTVelo. Additional comparisons would undoubtedly have value on their own; however, we believe that deeper analyses would constitute an evaluation of the aforementioned tools, rather than Cell Journey itself. We followed your suggestion and performed several **performance tests** to provide a clearer picture of the computation time for specific tasks. We present the results of the performance tests in **Figure 4** of the main manuscript.

We have previously investigated the advantages of **3D versus 2D visualizations** for single-cell developmental trajectories in a separate manuscript in which we introduced a new visualization and analysis method (**Ocelli** with **Multimodal Diffusion Maps**) for developmental multimodal single-cell datasets (P. Rutkowski, M. Tabaka, *NAR Genomics and Bioinformatics* (2025), 7(2), lqaf040; see Supplementary Materials). Here, we additionally include **2D and 3D embeddings** of a multimodal (RNA+ATAC) **Bone Marrow Mononuclear Cells (BMMCs)** dataset (Hu, Y et al. *Nature* (2025), 638, 779-786) to illustrate cases in which 3D visualization more clearly separates lineage branches and better preserves continuity along well-established hematopoietic trajectories (an interactive 3D view is provided as "Cell Journey BMMC 3D.html," viewable in any modern web browser). This is particularly important for large-

scale single-cell datasets, where high cell counts increase visual crowding and exacerbate projection-induced overlap. Moreover, when transitional cell populations are rare or sparsely sampled, low-dimensional embeddings are more prone to producing artificial overlaps and discontinuities, which can obscure or fragment developmental trajectories. In particular, the 3D embedding can reduce apparent crossings and overlaps introduced by 2D embeddings, making branching structure easier to interpret during interactive exploration (Supporting Figs. 1–2). The 2D and 3D embeddings were computed using the same method and the same input data; the only difference is the target dimensionality (2 versus 3). We emphasize that the primary goal of **Cell Journey** is to provide a comprehensive, interactive platform for visualization and exploratory analysis of RNA velocity and developmental trajectories. This is especially valuable in biological systems where the "ground-truth" trajectory is not known a priori, and where projection-induced crossings can confound interpretation of developmental relationships and result in misidentification of branching structure.

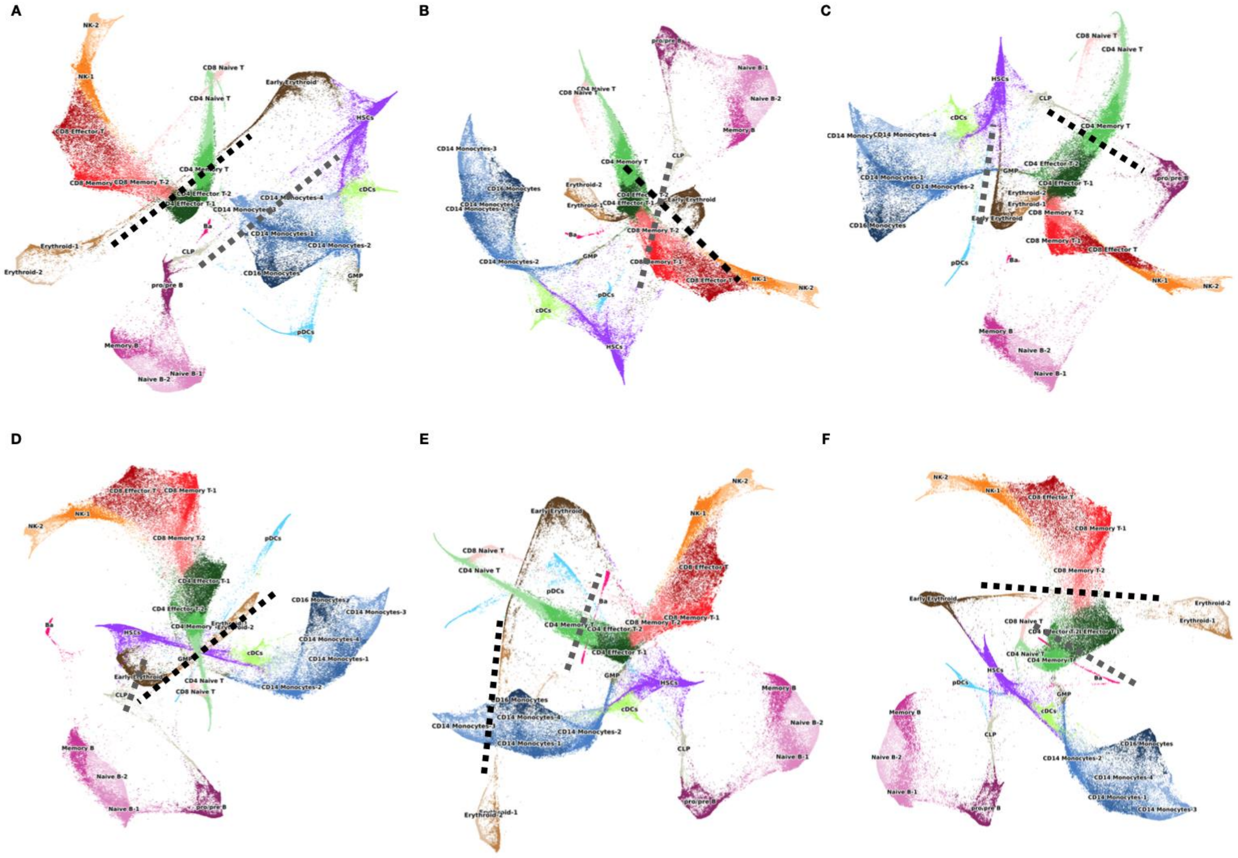

Supporting Fig. 1

Supporting Fig. 1: 2D embeddings of the BMMCs dataset exhibit lineage crossings in complex developmental topologies. Visualization of 2D embeddings generated with different random seeds for the single-cell BMMCs dataset (Hu, Y. et al., *Nature* (2025), 638, 779–786). In developmental systems with complex, multipartite transition topologies, 2D projections can introduce apparent crossings and overlaps between distinct lineages. The BMMC system includes both cells differentiating from hematopoietic stem/progenitor cells and lymphocytes (T and B cells) that mature in other organs. We highlight

projection-induced crossings using dotted black and gray lines in the 2D visualizations: (**A**) black - erythroid lineage with T cells; gray - B-cell lineage with monocytes; (**B**) black - erythroid lineage with T cells; gray - B-cell lineage with T cells and erythroid lineage; (**C**) black - B-cell lineage with T cells; gray - pDCs with monocytes; (**D**) black - erythroid lineage with T cells and HSCs; gray - B-cell lineage with erythroid lineage; (**E**) black - erythroid lineage with monocytes; gray - basophils with monocytes; (**F**) black - erythroid lineage with T cells; gray - basophils with monocytes.

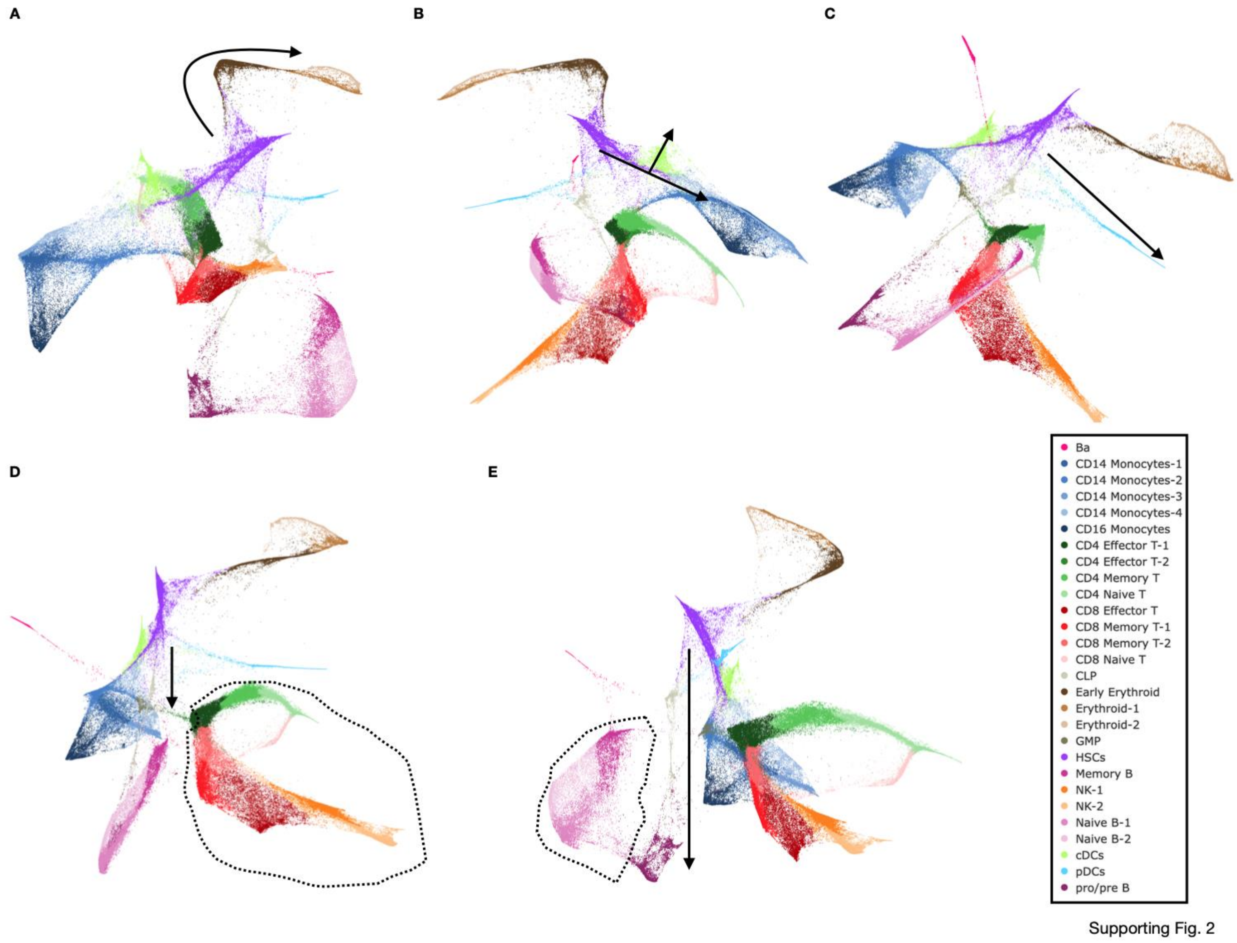

Supporting Fig. 2: 3D embedding resolves projection-induced lineage crossings in the BMMCs dataset. Two-dimensional renderings of the same 3D embedding shown from multiple viewing orientations for the multimodal (RNA+ATAC) BMMCs dataset (Hu, Y. et al., *Nature* (2025), 638, 779–786). In contrast to 2D embeddings (Supporting Fig. 1), the 3D representation substantially reduces projection-induced overlap and minimizes apparent lineage crossings, enabling clearer separation and continuity of major hematopoietic trajectories and facilitating interpretation of branching structure during interactive exploration. Arrows indicate the direction of known differentiation trajectories. Panels show: (**A**) erythroid lineage; (**B**) myeloid lineage, including trajectories toward cDCs and CD14/CD16 monocytes; (**C**) trajectory toward pDCs; (**D**) separation of the T-cell compartment; (**E**) lymphoid (B-cell) lineage with clear separation of mature B cells.

It would be helpful to clarify the distinguishing features of Cell journey, compared to existing visualization paradigms.

Our motivation for creating Cell Journey was to introduce several functionalities that, to the best of our knowledge, were not available in any existing software. We compared Cell Journey with nine popular visualization tools, including CELLxGENE, Vitessce, and the UCSC Cell Browser. This comparison is summarized in **Table 1** of the main manuscript. Notably, **Cell Journey** is the only tool that supports 3D visualization of velocity-based transitions and one-click extraction of a 3D trajectory for a selected cell, together with an interactive heatmap of features changing along that trajectory. Please see Table 1 for the full comparison.

Introduce more recent related packages developed for single-cell data analysis, such as pmid: 38290765, 32415966.

We thank the Reviewer for pointing us to additional recent single-cell analysis packages (PMID: 38290765; PMID: 32415966). These tools (scVAE and scbean) are high-quality, stand-alone frameworks that provide functionality for upstream tasks such as representation learning, denoising, and preprocessing.

We note, however, that the space of single-cell analysis methods is very large and rapidly evolving, and it is not feasible to comprehensively incorporate or benchmark all external packages within the scope of this manuscript. Our primary focus is on **velocity-based, interactive 3D visualization of cellular transitions and trajectory exploration**, rather than on proposing or consolidating new preprocessing or latent-representation methods.

At the current stage of development, Cell Journey therefore relies on widely adopted, standard components (e.g., Scanpy/AnnData-based workflows together with SciPy and scikit-learn) and is intended to operate downstream of a variety of preprocessing and modeling approaches. In practice, users can apply tools such as scVAE/scbean (or other pipelines) for preliminary processing and embedding construction, and then use Cell Journey to visualize inferred transitions in 3D and interactively interrogate trajectories and feature dynamics. For the same reason, we do not aim to replicate or directly integrate specialized upstream tools for RNA splicing quantification (e.g., **velocyto**) or velocity inference and transition estimation (e.g., **scVelo**, **UniTVelo**, **MultiVelo**, and related methods). Instead, Cell Journey is designed to consume their outputs (e.g., velocity vectors, transition graphs, or embeddings) and provide a unified, interactive 3D environment for exploration and interpretation of the resulting cellular dynamics.

We have revised the manuscript to include citations to these packages and to acknowledge them as complementary tools for upstream representation learning and preprocessing. Although tighter integration is feasible, providing and maintaining robust support across multiple third-party frameworks would require substantial modifications to the codebase and user interface and is beyond the scope of this manuscript.

Reviewers #2 and #3

In this study, the authors developed a platform called Cell Journey. It enables three-dimensional visualization of RNA velocity trajectories from uni- and multimodal single cell datasets. The platform has an intuitive graphical interface, allowing users to input from three data types, configure features according to their needs, and export results in a customizable manner. Overall, the work represents a valuable resource for the field, and I believe it should be accepted for publication after minor revisions. The authors provided documentation and a GitHub repository with clear instructions.

We appreciate your recognition of the value of Cell Journey, as well as many functionalities implemented in it. We are pleased with the positive reception, and grateful for pointing out elements which, after some modifications, can significantly improve the user experience and overall quality of the software.

After running celljourney, the platform does not automatically open in a browser. I had to manually copy and paste the provided address into my browser. Please consider including an explicit instruction such as: "copy this link into your browser". This addition would be especially useful for researchers without prior command line experience, as they might otherwise assume the platform is not working. A copy of my code is included below for reference.

This is a great suggestion, which has indeed noticeably improved the convenience of running the program. From now on, Cell Journey automatically opens in the browser. At the same time, we have retained the option to run it in the previous way by adding the --suppressbrowser flag. Information about this functionality has been added to the documentation. In the case of the Docker version, the application startup remains unchanged, due to the limitations of running it within an isolated container environment.

In the interface, please consider modifying the upload instruction to something clearer, as: "Drag and drop or click here to select a file to upload". Initially, I clicked on the blue button "Upload data" expecting a window to open, and it took me a while to realize how to select my file, an error was also triggered in my terminal. Making this instruction more explicit would improve usability for first-time users.

Thank you for pointing out the elements that may not be intuitive for new users and could hinder analysis with the Cell Journey. As the developers of this software, it is very challenging for us to notice such ambiguities ourselves, which is why we greatly appreciate this kind of feedback and were glad to implement the suggested changes exactly as proposed. In addition to renaming the field, we modified the title of the button below to ensure that users have no doubts about which element they should interact with first (see the table below).

Before

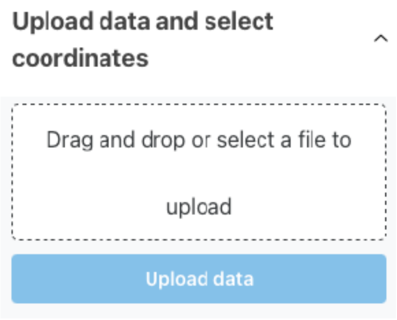

After

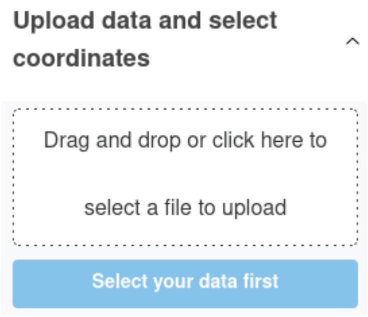

A copy of the output error is included below for reference.

```
[2025-09-23 06:53:16,659] ERROR in app: Exception on /_dash-update-component [POST] Traceback (most recent call last): File "/usr/local/lib/python3.11/site-packages/flask/app.py", line 1463, in wsgi_app response = self.full_dispatch_request() ^^^^^^^^^^^^^^^^^^^^^^^^^^^^ File "/usr/local/lib/python3.11/site-packages/flask/app.py", line 872, in full_dispatch_request rv = self.handle_user_exception(e) ^^^^^^^^^^^^^^^^^^^^^^^^^^^^^ File "/usr/local/lib/python3.11/site-packages/flask/app.py", line 870, in full_dispatch_request rv = self.dispatch_request() ^^^^^^^^^^^^^^^^^^^^^^^ File "/usr/local/lib/python3.11/site-packages/flask/app.py", line 855, in dispatch_request return self.ensure_sync(self.view_functions[rule.endpoint])(**view_args) # type: ignore[no-any-return] ^^^^^^^^^^^^^^^^^^^^^^^^^^^^^^^^^^^^^^^^^^^^^^^^^^^^^^^^^^^^^^^^^ File "/usr/local/lib/python3.11/site-packages/dash/dash.py", line 1310, in dispatch ctx.run( File "/usr/local/lib/python3.11/site-packages/dash/_callback.py", line 442, in add_context output_value = func(*func_args, **func_kwargs) # %% callback invoked %% ^^^^^^^^^^^^^^^^^^^^^^^^^^^^^^^ File "/app/celljourney.py", line 656, in upload_data_update_output _, content_data = contents.split(',') ^^^^^^^^^^^^^^ AttributeError: 'NoneType' object has no attribute 'split'
```

The identified error occurred when the program attempted to process data that the user had not yet uploaded. Thank you for bringing this to our attention – the software should not respond in such situations. We have fixed this issue, and it should no longer occur.

Please consider changing the instruction to something more explicit, such as: "Click to upload name-of-data.h5ad." At first, I assumed the data would be uploaded automatically after selection, and it took me a while to realize that I needed to click again. The process only became clear once I saw the confirmation message indicating that the file was successfully uploaded. Updating the wording would help prevent confusion for new users.

We agree that the suggested solution is more straightforward for the users. We have modified the layout exactly as recommended (see the table below).

Before

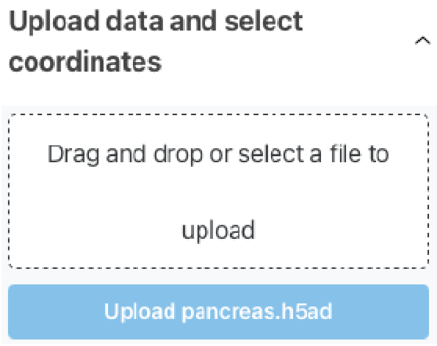

After

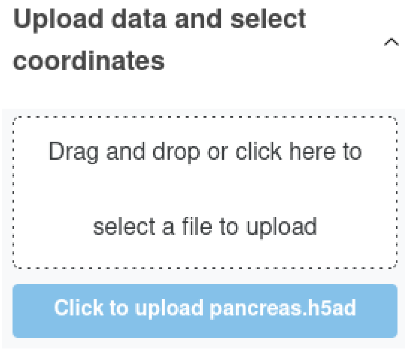

Please consider placing the legend outside the plot area, for example stacked at the top. Depending on how the user positions or rotates the three-dimensional visualization, the legend sometimes overlaps with the data, making it harder to interpret the trajectories. Moving the legend outside the main plotting space would improve clarity.

We agree that an incorrectly positioned legend may obscure the chart. We have implemented the ability to manually adjust the legend's position to generate compact charts that utilize all the available space (see example below on the left). Improper use of this functionality can indeed affect chart readability. However, we expect that users will generally rely on the typical positions, such as the far left or right side of the plotting area. The figure itself can also be moved freely in the case of any overlaps that need to be mitigated. Users unfamiliar with Plotly may be unaware of such functionalities, so we have added a tooltip that, when hovered over, displays a brief message with keyboard shortcuts to facilitate figure manipulation (see example below).

**New tooltip in the global plot configuration section**	**The list of tips expands when clicked**
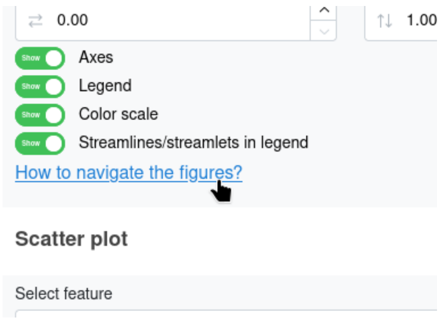	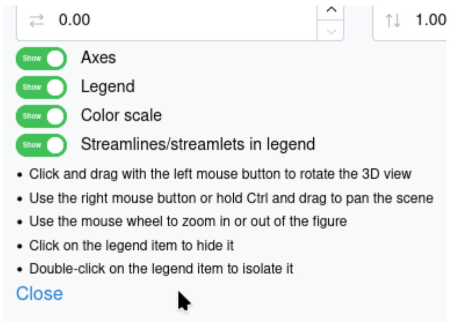

After plotting, if I change the global configuration settings, for example, adjusting the legend's horizontal or vertical position, the display of the plot becomes distorted. It would be helpful to improve the handling of these configuration changes so that the plot remains properly aligned and interpretable after adjustments.

Thank you for bringing this to our attention. We are not entirely sure whether we were able to reproduce the very behavior you depicted; however, in this context, we noticed a potentially problematic issue with the legend in horizontal orientation when it contains many elements – particularly dozens or hundreds of streamlets/streamlines. In such scenarios legend can indeed cover most of the plotting area. We have implemented a new option in the "global plot configuration" section that allows users to control the maximum height of the legend when it is in horizontal orientation. This limitation should prevent unwanted behavior like the one you described and similar ones as well (see the examples below).

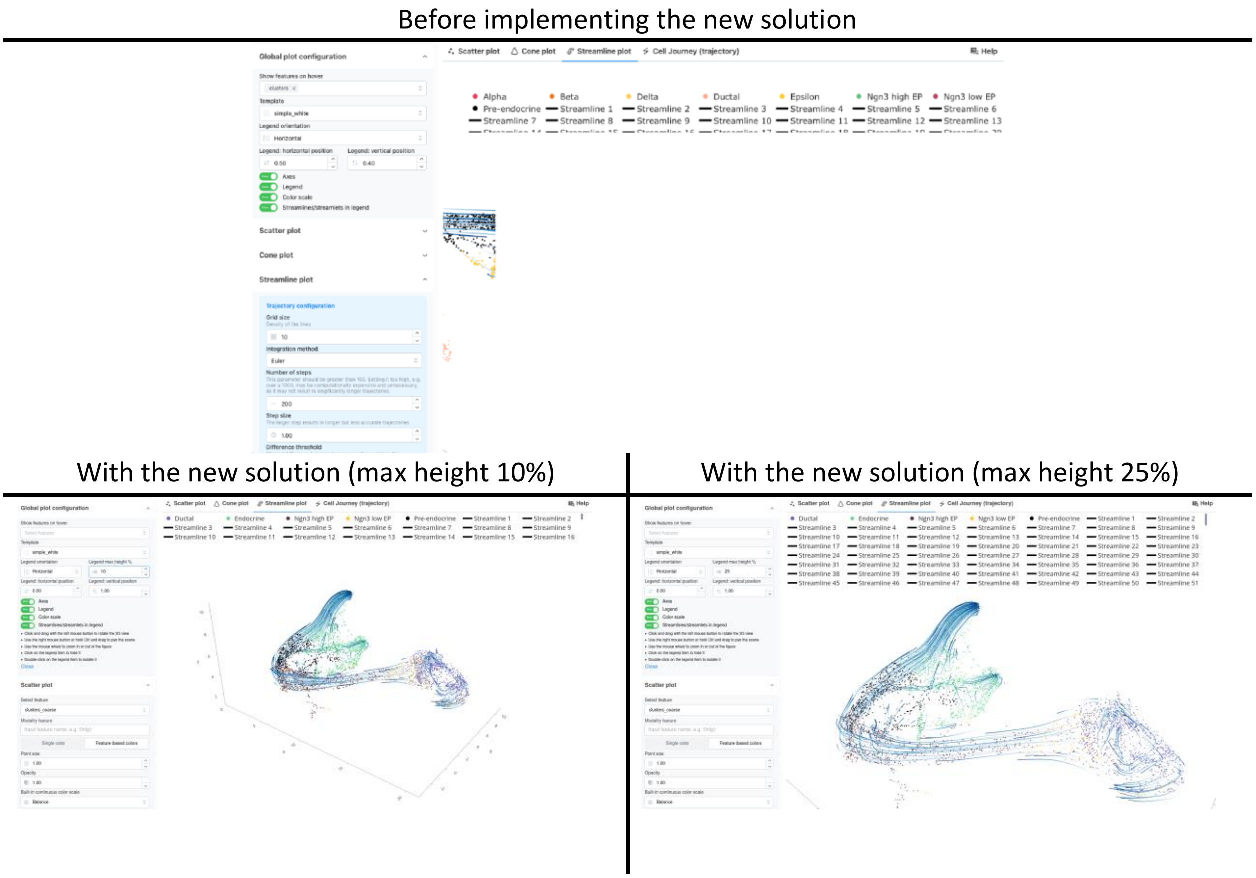

When I accidentally right clicked on the page and selected Reload, I lost all progress and had to start over from the beginning. It would be highly beneficial to implement a safeguard (autosave or session recovery), so users do not lose their work in such cases.

This is a valid point. We are aware of this behavior and acknowledge that it can be potentially problematic. Unfortunately, the fundamental way components are created and managed in Dash does not provide a convenient mechanism for handling memory in the manner described. While it is generally possible to implement such functionality, doing so would involve a non-standard approach to software development in Dash. Adapting Cell Journey accordingly would require a complete redesign of the codebase, which we plan to undertake in the future. We agree that the ability to save the program state is a significant feature that will undoubtedly improve both reproducibility and the overall user experience.

In the modality feature, please consider changing the instruction to: "Insert gene name." This phrasing would make the functionality clearer and more straightforward for users.

We agree that such a modification can improve the interface's readability. We have implemented it almost exactly as suggested. We decided to keep an example feature name to give a user a starting point (see the examples from the multimodal dataset on the bottom right).

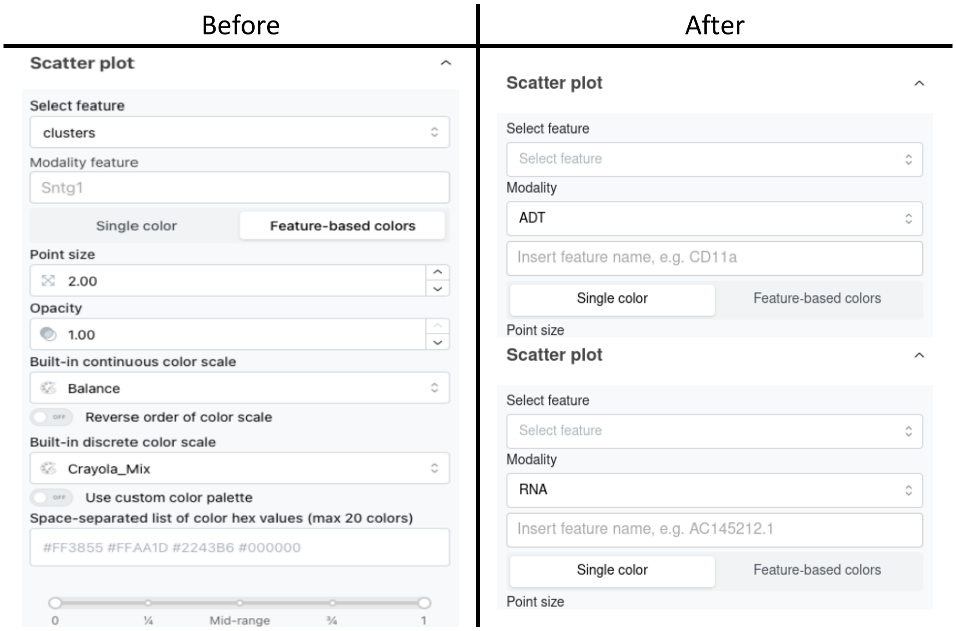

I was unable to export figures or CSV tables. Even after clicking the export option, nothing happened. Ensuring that the export functionality works as expected (or clarifying in the documentation if additional steps are required) would significantly improve the platform's usability.

Thank you for pointing out this critical issue. Saving results is undoubtedly one of the most important functionalities of the program. We thoroughly inspected the "export results" module and discovered that, when using a Docker container, our solution did not save results to the expected location. We decided to rebuild the entire module and base it on a different saving component that allows users to open a dialog window and select the desired location. This approach is likely more familiar and intuitive for users. The new solution works both in the standard and containerized version.

In the manuscript, the authors mention that the tool can be employed to visualize ground-truth cellular transitions obtained from lineage-tracing experiments for example Cas9-based ancestry recording or DNA barcoding. It would be very valuable if the documentation included a short tutorial or example dataset demonstrating these types of applications. This would help users directly connect the platform's features to the broader use cases discussed in the paper.

Initially, we assumed that users could perform clonal analysis by appropriately preparing the dataset and depicting the results through metadata. However, we decided to implement a dedicated module that enables this analysis without requiring any additional steps from the user (see the before-after comparison below).

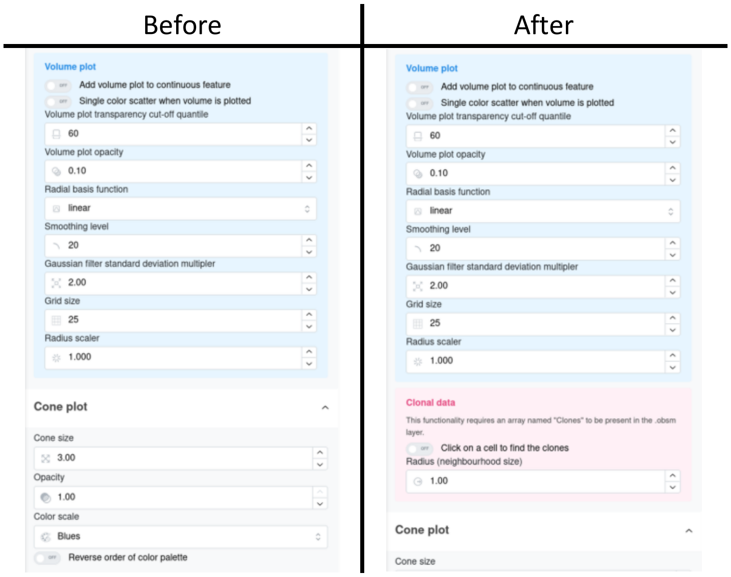

From now on, one can click on any cell to identify its clones or clones within a specified radius. Cell Journey offers two ways to visualize this data: using standard scatter plot with qualitative coloring, or a volume plot (see examples below). The only prerequisite is the presence of a data frame named 'Clones' in the obsm layer. This strict naming convention is a deliberate design choice: during data loading, Cell Journey automatically adds all column names from obsm to its selectors, such as embedding coordinates or visualization metadata. Including thousands of clonal entries in these selectors would unnecessarily overload Cell Journey and complicate the user experience. We have also updated the documentation to provide a step-by-step tutorial on how to prepare the appropriate dataset and how to utilize the new module.

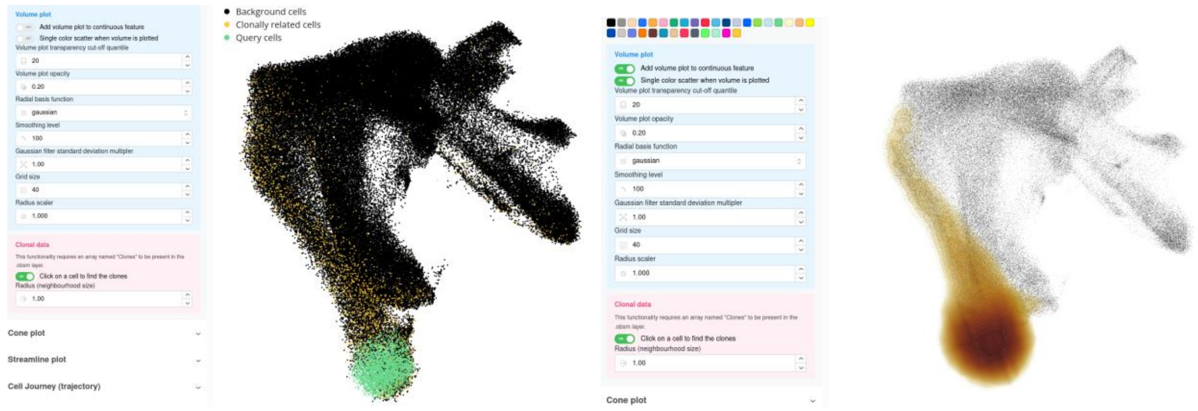

Below I include the errors from the terminal as reference. These may help the authors identify potential issues related to the usability problems described above.

```
[2025-09-23 06:38:29,961] ERROR in app: Exception on /_dash-update-component [POST] Traceback (most recent call last): File "/usr/local/lib/python3.11/site-packages/flask/app.py", line 1463, in wsgi_app response = self.full_dispatch_request() ^^^^^^^^^^^^^^^^^^^^^^^^^^^^ File "/usr/local/lib/python3.11/site-packages/flask/app.py", line 872, in full_dispatch_request rv = self.handle_user_exception(e) ^^^^^^^^^^^^^^^^^^^^^^^^^^^^^ File "/usr/local/lib/python3.11/site-packages/flask/app.py", line 870, in full_dispatch_request rv = self.dispatch_request()

...

AttributeError: 'NoneType' object has no attribute 'split' [2025-09-23 07:40:07,069] ERROR in app: Exception on /_dash-update-component [POST] Traceback (most recent call last): File "/usr/local/lib/python3.11/site-packages/flask/app.py", line 1463, in wsgi_app response = self.full_dispatch_request() ^^^^^^^^^^^^^^^^^^^^^^^^^^^^ File "/usr/local/lib/python3.11/site-packages/flask/app.py", line 872, in full_dispatch_request rv = self.handle_user_exception(e) ^^^^^^^^^^^^^^^^^^^^^^^^^^^^^ File "/usr/local/lib/python3.11/site-packages/flask/app.py", line 870, in full_dispatch_request rv = self.dispatch_request() ^^^^^^^^^^^^^^^^^^^^^^^ File "/usr/local/lib/python3.11/site-packages/flask/app.py", line 855, in dispatch_request return self.ensure_sync(self.view_functions[rule.endpoint])(**view_args) # type: ignore[no-any-return] ^^^^^^^^^^^^^^^^^^^^^^^^^^^^^^^^^^^^^^^^^^^^^^^^^^^^^^^^^^^^^^^^^^^^^ File "/usr/local/lib/python3.11/site-packages/dash/dash.py", line 1310, in dispatch ctx.run( File "/usr/local/lib/python3.11/site-packages/dash/_callback.py", line 442, in add_context output_value = func(*func_args, **func_kwargs) # %% callback invoked %% ^^^^^^^^^^^^^^^^^^^^^^^^^^^^^^^ File "/app/celljourney.py", line 656, in upload_data_update_output _, content_data = contents.split(',') ^^^^^^^^^^^^^^ AttributeError: 'NoneType' object has no attribute 'split'
[======================================================================] 100%
[======================================================================] 100%
[======================================================================] 100%
[======================================================================] 100%
[======================================================================] 100%
[======================================================================] 100%
[======================================================================] 100%
[======================================================================] 100%
```

Thank you for providing the logs that indicate the program's incorrect behavior. We have made an effort to identify all these issues and have implemented safety mechanisms to prevent their recurrence, primarily through improved exception handling. We believe that the updated version of Cell Journey runs more smoothly and is free from the reported issues.

Reviewer #4

Celljourney is a powerful visualization tool for 3d visualization of scRNAseq in 3 dimensions especially for RNA velocity analysis. The paper is well written while being both concise and complete. The documentation and code installation instructions are easy to follow and include a docker container. The figures are both visually appealing and do a good job at showing celljourney's capabilities. Celljourney provides a clear improvement on the current state of the art and is an excellent contribution to the field. I have almost no complaints. I wish I had more to add, but this is good work.

We are pleased that both the software and the article have received positive feedback. We devoted a great deal of time to developing all the Cell Journey functionalities, most of which were implemented in response to real needs and interest in using such solutions for our own analyses. Being acknowledged by experts in the field is very motivating and propels us to further develop the software.

At first I thought there wasn't an example dataset until I went to the documentation page separate from the github. I have now been able to recreate and try out Celljourney. Maybe mention that in the github page?

Thank you for your suggestion. As new users of various bioinformatics tools ourselves, we recognize the importance of being able to quickly and conveniently test a program on an example dataset provided by the authors. Following your advice, we have added a note about the datasets in the GitHub README.md file.

I did find some typos in your documentation. These things were easy to figure out. Just why not make it copy-paste for first time users. You should import scvello as scv and import scanpy as sc; adata_slim.write("pancreas_slim.h5ad) should close the quotes; you should import os before using it.

We apologize for all the errors and inconsistencies found in the documentation. We have carefully reviewed it again and corrected all the identified issues, as well as additional ones we encountered during the revision process.
