# Supplementary material for: Interactive analysis of single-cell trajectories in 3D space with Cell Journey
Source: Gigascience. 2026 Mar 3;15:giag021. doi: 10.1093/gigascience/giag021 (PMC13042281; doi:10.1093/gigascience/giag021)
Supplement: giag021_Reviewer_1_Report_original_submission — Reviewer 1 -- 9/23/2025 [file giag021_reviewer_1_report_original_submission.pdf]

**Reviewer Report**

**Title: Interactive analysis of single-cell trajectories in 3D space with Cell Journey**

**Version: Original Submission** **Date:** 9/23/2025

**Reviewer name: Reviewer 1**

**Reviewer Comments to Author:**

The manuscript introduces Cell Journey, an interactive visualization platform designed for three-dimensional (3D) analysis and representation of RNA velocity trajectories from single-cell datasets. The proposed tool appears to address an important gap in the field and could be of significant interest to the single-cell community. However, it may be still improved by the following suggestions :1 This is a promising manuscript that presents a potentially impactful visualization platform for single-cell RNA velocity analysis. However, to fully demonstrate the utility and adoption potential of Cell Journey, the authors should strengthen validation, provide more concrete examples of biological insights gained, and address usability and performance aspects.2 The manuscript would benefit from more rigorous validation of Cell Journey. For example:How do the 3D velocity trajectories compare to known biological ground truths (e.g., hematopoietic differentiation)?Are there quantitative benchmarks demonstrating that 3D visualization uncovers relationships not visible in 2D?3 It would be helpful to clarify the distinguishing features of Cell journey, comared to existing visualization paradigms.4 introduce more recent related packages developed for single-cell data analysis, such as pmid: 38290765, 32415966

**Level of Interest**

Please indicate how interesting you found the manuscript: Choose an item.

**Quality of Written English**

Please indicate the quality of language in the manuscript: Choose an item.

**Declaration of Competing Interests**

Please complete a declaration of competing interests, considering the following questions:

- Have you in the past five years received reimbursements, fees, funding, or salary from an organisation that may in any way gain or lose financially from the publication of this manuscript, either now or in the future?
- Do you hold any stocks or shares in an organisation that may in any way gain or lose financially from the publication of this manuscript, either now or in the future?
- Do you hold or are you currently applying for any patents relating to the content of the manuscript?

- Have you received reimbursements, fees, funding, or salary from an organization that holds or has applied for patents relating to the content of the manuscript?
- Do you have any other financial competing interests?
- Do you have any non-financial competing interests in relation to this paper?

If you can answer no to all of the above, write 'I declare that I have no competing interests' below. If your reply is yes to any, please give details below.

no. I declare that I have no competing interests

I agree to the open peer review policy of the journal. I understand that my name will be included on my report to the authors and, if the manuscript is accepted for publication, my named report including any attachments I upload will be posted on the website along with the authors' responses. I agree for my report to be made available under an Open Access Creative Commons CC-BY license (http://creativecommons.org/licenses/by/4.0/). I understand that any comments which I do not wish to be included in my named report can be included as confidential comments to the editors, which will not be published.

Choose an item.

To further support our reviewers, we have joined with Publons, where you can gain additional credit to further highlight your hard work (see: https://publons.com/journal/530/gigascience). On publication of this paper, your review will be automatically added to Publons, you can then choose whether or not to claim your Publons credit. I understand this statement.

Yes Choose an item.
