# Supplementary material for: Interactive analysis of single-cell trajectories in 3D space with Cell Journey
Source: Gigascience. 2026 Mar 3;15:giag021. doi: 10.1093/gigascience/giag021 (PMC13042281; doi:10.1093/gigascience/giag021)
Supplement: giag021_Reviewer_3_Report_original_submission — Reviewer 3 -- 10/14/2025 [file giag021_reviewer_3_report_original_submission.pdf]

Reviewer Report

Title: Interactive analysis of single-cell trajectories in 3D space with Cell Journey

Version: Original Submission **Date:** 10/14/2025

Reviewer name: Reviewer 3

Reviewer Comments to Author:

Celljourney is a powerful visualization tool for 3d visualization of scRNAseq in 3 dimensions especially for RNA velocity analysis.The paper is well written while being both concise and complete. The documentation and code installation instructions are easy to follow and include a docker container. The figures are both visually appealing and do a good job at showing celljourney's capabilities. Celljourney provides a clear improvement on the current state of the art and is an excellent contribution to the field.I have almost no complaints. I wish I had more to add, but this is good work.At first I thought there wasn't an example dataset until I went to the documentation page separate from the github. I have now been able to recreate and try out Celljourney. Maybe mention that in the github page?I did find some typos in your documentation. These things were easy to figure out. Just why not make it copy-paste for first time users.you should import scvello as scv and import scanpy as scadata_slim.write("pancreas_slim.h5ad) should close the quotesyou should import os before using it.

Level of Interest

Please indicate how interesting you found the manuscript: Choose an item.

Quality of Written English

Please indicate the quality of language in the manuscript: Choose an item.

Declaration of Competing Interests

Please complete a declaration of competing interests, considering the following questions:

- Have you in the past five years received reimbursements, fees, funding, or salary from an organisation that may in any way gain or lose financially from the publication of this manuscript, either now or in the future?
- Do you hold any stocks or shares in an organisation that may in any way gain or lose financially from the publication of this manuscript, either now or in the future?
- Do you hold or are you currently applying for any patents relating to the content of the manuscript?
- Have you received reimbursements, fees, funding, or salary from an organization that holds or has applied for patents relating to the content of the manuscript?
- Do you have any other financial competing interests?

- Do you have any non-financial competing interests in relation to this paper?

If you can answer no to all of the above, write 'I declare that I have no competing interests' below. If your reply is yes to any, please give details below.

I declare that I have no competing interests

I agree to the open peer review policy of the journal. I understand that my name will be included on my report to the authors and, if the manuscript is accepted for publication, my named report including any attachments I upload will be posted on the website along with the authors' responses. I agree for my report to be made available under an Open Access Creative Commons CC-BY license (http://creativecommons.org/licenses/by/4.0/). I understand that any comments which I do not wish to be included in my named report can be included as confidential comments to the editors, which will not be published.

Choose an item.

To further support our reviewers, we have joined with Publons, where you can gain additional credit to further highlight your hard work (see: https://publons.com/journal/530/gigascience). On publication of this paper, your review will be automatically added to Publons, you can then choose whether or not to claim your Publons credit. I understand this statement.

Yes Choose an item.
